# Extracellular vesicle-packaged miRNA release after short-term exposure to particulate matter is associated with increased coagulation

**DOI:** 10.1186/s12989-017-0214-4

**Published:** 2017-08-24

**Authors:** Laura Pergoli, Laura Cantone, Chiara Favero, Laura Angelici, Simona Iodice, Eva Pinatel, Mirjam Hoxha, Laura Dioni, Marilena Letizia, Benedetta Albetti, Letizia Tarantini, Federica Rota, Pier Alberto Bertazzi, Amedea Silvia Tirelli, Vincenza Dolo, Andrea Cattaneo, Luisella Vigna, Cristina Battaglia, Michele Carugno, Matteo Bonzini, Angela Cecilia Pesatori, Valentina Bollati

**Affiliations:** 1 EPIGET LAB, Department of Clinical Sciences and Community Health, Università degli Studi di Milano, Via san Barnaba 8, 20122 Milan, Italy; 2 Fondazione IRCCS Ca’ Granda Ospedale Maggiore Policlinico, Unit of Occupational Medicine, Milan, Italy; 3 Institute for Biomedical Technologies (ITB), National Research Council (CNR), Segrate, Milan, Italy; 4 Department of Life, Health and Environmental Sciences, University of L’Aquila, L’Aquila, Italy; 5 Department of Science and High Technology, University of Insubria, Como, Italy; 6 Department of Medical Biotechnology and Translational Medicine, Università degli Studi di Milano, 20129 Milan, Italy

**Keywords:** Air pollution, Extracellular vesicles, microRNAs, Fibrinogen, Cardiovascular disease

## Abstract

**Background:**

Exposure to particulate matter (PM) is associated with increased incidence of cardiovascular disease and increased coagulation, but the molecular mechanisms underlying these associations remain unknown. Obesity may increase susceptibility to the adverse effects of PM exposure, exacerbating the effects on cardiovascular diseases. Extracellular vesicles (EVs), which travel in body fluids and transfer microRNAs (miRNAs) between tissues, might play an important role in PM-induced cardiovascular risk. We sought to determine whether the levels of PM with an aerodynamic diameter ≤ 10 μm (PM_10_) are associated with changes in fibrinogen levels, EV release, and the miRNA content of EVs (EV-miRNAs), investigating 1630 overweight/obese subjects from the SPHERE Study.

**Results:**

Short-term exposure to PM_10_ (Day before blood drawing) was associated with an increased release of EVs quantified by nanoparticle tracking analysis, especially EVs derived from monocyte/macrophage components (CD14+) and platelets (CD61+) which were characterized by flow cytometry. We first profiled miRNAs of 883 subjects by the QuantStudio™ 12 K Flex Real Time PCR System and the top 40 EV-miRNAs were validated through custom miRNA plates. Nine EV-miRNAs (let-7c-5p; miR-106a-5p; miR-143-3p; miR-185-5p; miR-218-5p; miR-331-3p; miR-642-5p; miR-652-3p; miR-99b-5p) were downregulated in response to PM_10_ exposure and exhibited putative roles in cardiovascular disease, as highlighted by integrated network analysis. PM_10_ exposure was significantly associated with elevated fibrinogen levels, and five of the nine downregulated EV-miRNAs were mediators between PM_10_ exposure and fibrinogen levels.

**Conclusions:**

Research on EVs opens a new path to the investigation of the adverse health effects of air pollution exposure. EVs have the potential to act both as markers of PM susceptibility and as potential molecular mechanism in the chain of events connecting PM exposure to increased coagulation, which is frequently linked to exposure and CVD development.

**Electronic supplementary material:**

The online version of this article (doi:10.1186/s12989-017-0214-4) contains supplementary material, which is available to authorized users.

## Background

According to the World Health Organization, air pollution poses a severe risk to cardiovascular (CV) health, with ~3% of cardiopulmonary deaths each year being attributable to particulate matter (PM) globally [1]. Acute or chronic PM exposure can trigger myocardial ischemia (MI), stroke, and arrhythmia, particularly in at-risk populations [2, 3].

Obesity is a strong risk factor for cardiovascular disease (CVD). Recent research findings identified obesity as a susceptibility factor to the adverse effects of PM exposure, due in part to increased particle deposition in the lower airways [4]. Obesity modifies the effects of PM exposure on heart rate variability and markers of inflammation, oxidative stress, and acute phase response [5–7]. However, the mechanisms linking PM exposure to CVD development have not been fully elucidated.

Ambient particles produce a strong inflammatory reaction in the lungs, but only a very small fraction of these particles accumulate in extrapulmonary organs, such as liver or spleen [8]. Currently, there is no conclusive evidence that PM physically enter and deposit in blood vessels, although a recent paper by Miller et al. showed a translocation of inhaled gold nanoparticles (<10 nm) into the systemic circulation and accumulation at sites of vascular inflammation, in healthy volunteers [9]. In this context, a cross-talk between the pulmonary and CV systems may underlie the observed peripheral effects of PM exposure [10]. Besides direct cell-cell contact and communication mediated by nonspecific soluble factors, cells communicate through extracellular membrane vesicles that are released in all body fluids. The importance of these extracellular vesicles (EVs) lies in their capacity to transfer specific information to other cells, thereby influencing recipient cell function [10].

EV cargo includes microRNAs (miRNAs): small, endogenous, single-stranded noncoding RNAs of 20–22 nucleotides that post-transcriptionally regulate gene expression by triggering mRNA cleavage or repressing translation [11]. EVs and EV-associated miRNAs (EV-miRNAs) might be ideal candidates for mediators of the CV effects of PM exposure. Specifically, EVs could be produced by the respiratory system [12], reach the systemic circulation, and transfer miRNAs [13] to recipient cells as the EVs travel throughout the body. These miRNAs could modulate target gene expression in recipient cells, thereby leading to CV dysfunction [14].

In the present study, we investigated the effects of exposure to PM with an aerodynamic diameter ≤ 10 μm (PM_10_) on the release and miRNA content of EVs in plasma from overweight/obese individuals [15]. We profiled miRNAs whose levels were altered in response to PM_10_ and estimated the proportion of PM_10_ effects on fibrinogen [16] that were mediated by differential miRNA expression. Bioinformatics analysis was used to investigate the putative mechanisms of action of the differentially expressed EV-miRNAs.

## Methods

### Study design and participants

Overweight/obese subjects were recruited at the Center for Obesity and Work (COW; Department of Preventive Medicine, IRCCS Fondazione Ca’Granda – Ospedale Maggiore Policlinico, Milan, Italy) between September 2010 and March 2015, as part of the cross-sectional study SPHERE.

(“Susceptibility to Particle Health Effects, miRNAs and Exosomes”). The study design and subject recruitment criteria have been extensively described in (16). Briefly, the eligibility criteria for participants are: 1) older than 18 years at enrollment; 2) obese/overweight according to the following definition: overweight is defined as a BMI between 25 and 30 kg/cm^2^, obesity is defined as a BMI of 30 kg/cm^2^ or more; 3) resident in Lombardy at the time of the recruitment; 4) agreement to sign an informed consent and donate blood and urine samples. Exclusion criteria include: previous diagnosis of cancer, heart disease or stroke in the last year or other chronic diseases such as multiple sclerosis, Alzheimer’s disease, Parkinson’s disease, depression, bipolar disorder, schizophrenia and epilepsy.

Each participant signed an informed consent form, which had been approved by the ethics committee of the institution (approval number 1425), in accordance with principles of the Helsinki Declaration. The participation rate of the overall recruitment was 92%. Body mass index (BMI) was calculated and categorized according to the current definition. [17] We followed a two-stage, split-sample study design for miRNA analysis. A diagram describing the study design is reported in Additional file 1.

Quantitative determination of fibrinogen in citrate plasma samples was obtained on automated I.L. Coagulation System (Instrumentation Laboratory S.p.A. Milan, Italy).

### PM exposure assessment

Under the hypothesis that a short-term mechanism underlies variations in EV and EV-miRNA levels, we chose to investigate a 1-week lag exposure time window before the day of recruitment (defined as Day −1). We collected daily PM_10_ data from the available fixed monitoring stations of the Air Quality Monitoring Network of the Regional Environmental Protection Agency (ARPA Lombardia). PM_10_ exposure data for years 2010–2012 were assessed by the ARPA chemical transport model (CTM) [18], which provided daily PM_10_ concentration estimates with a spatial resolution of 4 × 4 km. Using ArcGIS® software (Esri), we assigned to each subject the daily PM_10_ concentration from: (1) the nearest monitor to their home address (“subject residence”) for the 7 days preceding recruitment; (2) the nearest monitor to the COW (defined as “Policlinico”) for the day of recruitment; and (3) the 4 × 4 km cell of the CTM grid containing the subject’s residence.

Meteorological data were obtained from ARPA monitoring stations, which measured temperature (233 monitors) and relative humidity (163 monitors). Apparent temperature was calculated as previously reported [19]. Detailed explanation of the exposure assessment methods is reported elsewhere [20].

### Isolation and purification of EVs and miRNA-EVs from plasma

Blood was collected into EDTA tubes at the COW on the morning of recruitment (8 to 10 a.m.) and transported to the EPIGET Lab (University of Milan) within 2 h of phlebotomy. Isolation, extraction, and purification of EVs and EV-miRNAs from plasma were performed as described in the Additional file 2.-Supplemental Methods S1. For quality control, purified EVs were analyzed by transmission electron microscopy, following standard preparation protocols [21]. Examples of purified EV preparations are reported in Additional file 3.

### Nanoparticle tracking analysis (NTA)

Numbers and dimensions of EVs were assessed by NTA, a technique that measures the Brownian motion of vesicles suspended in fluid and displays them in real time through a CCD camera with high sensitivity. Using a NanoSight LM10-HS system (Amesbury, UK), EVs were visualized by laser light scattering. Five 30-s recordings were made for each sample. Collected data were analyzed with NTA software, which provided high-resolution particle-size distribution profiles and concentration measurements of the EVs.

### Flow cytometry

EVs were characterized by flow cytometry (MACSQuant, Miltenyi Biotec) according to a protocol for characterization of EVs [22]. Fluoresbrite® Carboxylate Size Range Kit I (0.2, 0.5, 0.75, and 1 μm) was used to set the calibration gate on the analyzer. EVs were stained before analysis as described in the Additional file 2- Supplemental Methods S2. Quantitative multiparameter analysis of flow cytometry data was carried out by using FlowJo Software (Tree Star, Inc.).

### Screening of miRNA expression

MiRNAs were prepared by standard reverse transcription (RT) and preamplification procedures (see Additional file 2- Supplemental Methods S3), followed by real-time RT-PCR analysis with the QuantStudio™ 12 K Flex OpenArray® Platform (QS12KFlex). Gene Expression Suite Software (Life Technologies) was used to process miRNA expression data from the miRNA panel.

We obtained 758 Crt values for each subject, which included 754 unique miRNAs and four internal controls (ath-miR159a, RNU48, RNU44 and U6). For each amplification curve, we obtained an AmpScore value, a quality measurement that indicates the low signal in the amplification curve linear phase (range: 0–2). MiRNAs with Crt value > 28 or AmpScore <1.1 or missing were considered unamplified, and the Crt value was set to 29. MiRNAs that were not amplified in all subjects (*n* = 209) were excluded, resulting in 545 miRNAs being included in the analysis. NormFinder [23] and geNorm [24] algorithms were used to select the best normalization strategy among global mean, RNU48, RNU6, or the average of the four miRNAs with the lowest standard deviation (SD) among subjects (hsa-miR-526b-5p, hsa-miR-186-5p, hsa-miR-496, and hsa-miR-541-3p). Global mean was selected as the best normalization method. MiRNA expression was determined using the relative quantification 2^-ΔCrt^ [25].

### Validation of miRNA expression

We identified the top 40 miRNAs that were the most associated with PM_10_ exposure on the day before recruitment and were expressed in at least 50% of subjects. These miRNAs were validated in triplicate by using similar preamplification, loading, and analyzing conditions as the screening phase, with minor modifications (see Additional file 2- Supplemental Methods S4).

### Statistical analysis

Quantitative data were expressed as the mean ± SD or as the median and interquartile range (Q1-Q3) as appropriate. Categorical data were presented as frequencies and percentages. Continuous variables were tested for normality and linearity. All multivariable models were adjusted for some a priori covariates: age, sex, BMI, smoking habits, regardless of the *p* value returned by respective univariate models. Other variables, such as apparent temperature, season of enrollment, education, occupation, alcohol consumption, blood pressure, heart rate, uric acid, cholesterol, triglyceride, creatinine, glycaemia, homocysteine, glycated hemoglobin, insulin, were additionally considered as possible confounders, but only apparent temperature at Day −1 resulted significantly associated with the outcomes and was therefore added into the models. Multiple linear regression analysis was used to evaluate the association between EVs (number and characterization) and PM exposure. EV counts and characterizations showed skewed distributions and were naturally log-transformed to approximately normal distributions of residuals. Effects were expressed as ∆%, which represents the percentage increase in EVs for each 10-μg/m^3^ increase in PM_10_ concentration. To analyze the possible effects of BMI, an interaction term between a two-category BMI variable (overweight and obese) and PM exposure was added in the multivariable regression model. Using the same two BMI categories, stratified linear regression models (adjusted for age, sex, smoking status, and apparent temperature) were run.

Given the results of the previous step, we used multivariable linear regression models to verify the association between PM_10_ exposure on the day before recruitment (Day −1–PM_10_) and miRNA expression in the screening and validation phases. MiRNA expression values were log2-transformed to achieve a normal distribution. Due to the high number of comparisons, we applied a multiple comparison correction method based on the Benjamini-Hochberg False Discovery Rate (FDR) to calculate the FDR *P*-value. In the screening phase, the criterion used to identify the top miRNAs was a FDR *P*-value <0.10 for miRNAs expressed in at least 50% of subjects. In the validation phase, miRNAs with raw *P* < 0.05 were considered differentially expressed. A volcano plot of Δ% vs. log_10_ *P*-values was used to display results of the screening and validation analyses.

Multivariable linear regression models were used to test the association between Day −1–PM_10_ exposure and fibrinogen levels (naturally log-transformed to approximate normality of residuals), which we hypothesized could be mediated through changes in miRNA expression. Each validated miRNA was separately considered as a potential mediator (M) in a simple mediation model. Two multivariable linear regression models were used for the association between (Eq. 1) PM_10_ exposure and M, and (Eq. 2) M and outcome/fibrinogen concentration (Y) [26, 27]:

1 $$ {M}_i={\beta}_0+{\beta}_1{X}_{1i}+\cdots +{\beta}_p{X}_{pi}+{\beta}_{PM10}{PM}_{10i}+{\varepsilon}_i. $$

2 $$ {Y}_i={\gamma}_0+{\gamma}_1{X}_{1i}+\cdots +{\gamma}_p{X}_{pi}+{\gamma}_{PM10}{PM}_{10i}+{\gamma}_M{M}_i+{\varepsilon}_i. $$

In these models, *γ*_*PM*10_ is the estimate of the direct effect, and the product *β*_*PM*10_^∗^ *γ*_*M*_ is the estimate of the indirect (mediated) effect. Bias-corrected bootstrap confidence intervals were provided, with the number of bootstrap samples equal to 10,000 [28]. Estimates correspond to 10 μg/m^3^ increase in PM_10_ concentration.

All statistical analyses were performed by using SAS 9.4 statistical software (SAS Institute Inc., Cary, NC) and STATA 13 (StataCorp. 2013, College Station, TX). Simple mediation analyses were executed on the SAS v9.4 macro, using the PROCESS program (model = 4) provided by Hayes (2013) [26].

### miRNA targets related to CVD and biological network analysis

To elucidate the possible biological mechanisms connecting EVs-miRNA content and the predisposition to CVD we performed an integrated network analysis. DisGeNET database (v4.0) [29] was used as source for the CVD related genes network: we manually selected among the diseases containing at least 20 associated genes (only expert-curated associations were evaluated) those related to CVD. Namely we considered: Myocardial Ischemia, Hypertensive Diseases, Inflammation, Heart Failure, Myocardial Infarction, Asthma, Thrombosis, Heart Diseases, Cardiovascular Diseases, Ischemia, Cerebral Hemorrhage, Atrial Fibrillation, Coronary Heart Disease, Cardiac Arrhythmia and their related genes. Next we built a miRNA-target interaction network containing the genes whose 3’UTR was predicted to interact with each of the miRNAs differentially expressed in response to PM. We considered as *bona-fide* miRNA-target interactions only those predicted by at least 2 algorithms among DIANA-microTv4.0, miRanda-rel2010, PicTar2, PITA, RNA22v2 and Targetscan6.2 (in the version provided by mirWalk 2.0 database) [30]. Integrating these two networks we identified significantly enriched EV-miRNAs (FDR < 0.1, Fisher test) among targets of specific CVDs and hub genes related to multiple CVDs and putatively affected by EVs-miRNAs alteration as relevant elements of the miRNA mediated risk of CVD.

Finally, given the correlation of five EV-miRNAs expression with fibrinogen levels, we integrated the miRNA-target subnetwork relative to these five miRNAs with the genes involved in the coagulation cascade according to KEGG, to identify the putative effectors of miRNA alteration.

Network integration and the relative images were elaborated with Cytoscape V3.4 [31].

## Results

### Characteristics and PM_10_ exposure of study participants

The study population included 1630 subjects (26.9% men, 73.1% women), with the first (discovery) and second (validation) stages of the study involving 883 and 747 consecutively recruited subjects, respectively. Main characteristics of the study subjects are reported in Table 1. The mean age of patients was 52.4 years. Participants were categorized according to their BMI as being overweight (26.9%; BMI: ≥25 to <30 kg/m^2^), obese (38.7%; BMI: ≥30 to <35 kg/m^2^), or severely obese (34.4%; BMI: ≥35 kg/m^2^). Nearly half of participants (48.7%) were never smokers, 35.3% were former smokers, and 15.8% were current smokers. Most participants (60.6%) were enrolled during the autumn and winter seasons, and most lived in the Milan city area (59.8%). The median fibrinogen concentration was 325 mg/dl. Screening and validation subsets were slightly different in terms of subject’s age, season of enrolment distribution and fibrinogen concentration (Table 1).

**Table 1:** Characteristics of study participants

Characteristic	Total population	Discovery subset	Validation subset	*P*
	*N* = 1630	*n* = 883	*n* = 747	
Sex				
Males	438 (26.9%)	237 (26.8%)	201 (26.9%)	0.9756
Females	1192 (73.1%)	646 (73.2%)	546 (73.1%)	
Age, years (mean ± SD)	52.4 ± 13.8	51.5 ± 13.5	53.5 ± 14.1	0.0050
BMI*, kg/m^2^ (mean ± SD)	33.6 ± 5.4	33.7 ± 5.6	33.5 ± 5.3	0.4758
BMI* categorical				
Overweight (25–30 kg/m^2^)	438 (26.9%)	238 (27.0%)	200 (26.8%)	0.7290
Obese (≥30 kg/m^2^)	1191 (73.0%)	645 (73.0%)	546 (73.1%)	
Missing	1 (0.1%)	_	1 (0.1%)	
Smoking status				
Never smoker	794 (48.7%)	447 (50.6%)	347 (46.5%)	0.3425
Former smoker	575 (35.3%)	303 (34.3%)	272 (36.4%)	
Current smoker	258 (15.8%)	131 (14.8%)	127 (17%)	
Missing	3 (0.2%)	2 (0.2%)	1 (0.1%)	
Year of enrollment				
2010	90 (5.5%)	90 (10.2%)	_	_
2011	409 (25.1%)	409 (46.3%)	_	
2012	384 (23.6%)	384 (43.5%)	_	
2013	304 (18.7%)	_	304 (40.7%)	
2014	324 (19.9%)	_	324 (43.4%)	
2015	119 (7.3%)	_	119 (15.9%)	
Season of enrollment				
Winter	487 (29.9%)	223 (25.3%)	264 (35.3%)	< 0.0001
Spring	430 (26.4%)	227 (25.7%)	203 (27.2%)	
Summer	213 (13.1%)	106 (12%)	107 (14.3%)	
Autumn	500 (30.7%)	327 (37%)	173 (23.2%)	
Living area				
City of Milan	975 (59.8%)	493 (55.8%)	482 (64.5%)	0.0004
Outside Milan	655 (40.2%)	390 (44.2%)	265 (35.5%)	
Fibrinogen, mg/dl (median [Q1-Q3])	325 [290–366]	328 [295–368]	322 [281–365]	0.0056

*BMI = Body mass index

The PM_10_ exposure during each time lag is reported in Additional file 4. The highest values measured in the discovery phase most likely resemble the PM_10_ time trend. All values measured in the discovery phase were higher than values measured in the validation phase (*P* < 0.0001).

### Association between ambient PM_10_ levels and EV quantification by NTA

To define the PM_10_ exposure window that was most effective in modifying EV release, we investigated the association between different time lags and EV count by NTA. The mean plasma EV was 207.5 nm, and the mode was 148.5 nm. A histogram showing the size distribution across samples is reported in Additional file 5. EV count by NTA descriptive statistics are reported in Additional file 6.

**Fig. 1:**
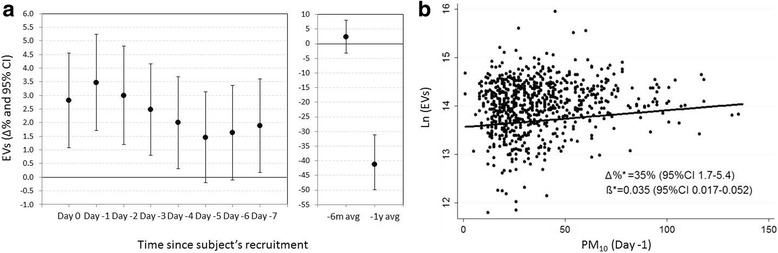
**a** Association of PM_10_ levels measured at different time lags (from day of blood drawing to the previous 7 days) with EV count by NTA. Δ% = (exp (β)-1)*100. **b** Association of individual Day −1–PM_10_ exposure with number of EVs measured by NTA. Models reported in both panels were adjusted for age, sex, BMI, smoking status, and apparent temperature

The positive effect of PM_10_ exposure on EV count was maximal on the day before recruitment (Day −1, Fig. 1a). On Day −1, for each 10-μg/m^3^ increase in PM_10_ concentration, we observed an increase in EV count (Δ% = 3.5%, *P* = 0.0001). Figure 1b reports the association between PM_10_ exposure on Day −1 and the number of EVs measured by NTA. Given this result, we focused all further analysis on Day −1. As we also considered long-term exposures (i.e. 6-months and yearly averages), we observed a negative significant association between PM_10_ yearly average and EV count (Δ% = −41.25%, *P* < 0.0001).

### Association between ambient PM_10_ levels and EV characterization by flow cytometry

According to our hypothesis, we chose a panel of EV markers that are characteristic of EV-releasing cells and possibly related to PM_10_ effects. We characterized five EV types (Additional file 6): CD61+ EVs (released from platelets), CD66+ EVs (released from neutrophils), EpCAM+ EVs (released from epithelial cells), CD105+ EVs (released from endothelium), and CD14+ EVs (released from monocytes). Table 2 reports the associations between Day −1–PM_10_ exposure and EV types released. A positive association was observed between PM_10_ and CD61+ EVs (Δ% = 5.27%, *P* = 0.0020), EpCAM+ EVs (Δ% = 2.97%, *P* = 0.0430), and CD14+ EVs (Δ% = 4.68%, *P* = 0.0030).

**Table 2:** Flow cytometry analysis of the association between PM10 exposure (Day −1) and cell-specific EV count, after adjustments for age, sex, BMI, smoking status, and apparent temperature

EV type	Δ%^a^	95% CI	*P*
CD61+ (platelets)	5.27	1.91; 8.73	0.0020
CD66+ (neutrophils)	1.94	−0.59; 4.54	0.1351
EpCAM+ (epithelium)	2.97	0.10; 5.93	0.0430
CD105+ (endothelium)	2.05	−0.22; 4.38	0.0776
CD14+ (macrophages/monocytes)	4.68	1.58; 7.87	0.0030

^a^Δ% = (exp (β*10)-1)*100, percentage increase in EV count for each 10-μg/m^3^ increase in PM_10_ concentration

Overweight subjects showed a greater effect of PM exposure in all cellular subtypes, whereas for obese subjects, the effect was significant only for CD61+ EVs (Δ% = 4.65%, *P* = 0.0195; Table 3).

**Table 3:** Flow cytometry analysis of association between PM_10_ exposure (Day −1) and cell-specific EV count, after BMI stratification and adjustment for age, sex, smoking status, and apparent temperature

	Overweight (25 ≤ BMI < 30 kg/m^2^)			Obese (BMI ≥ 30 kg/m^2^)			*P for Interaction*
EV type	Δ%^a^	95% CI	*P*	Δ%	95% CI	*P*	
CD61+ (platelets)	7.58	0.96; 14.62	0.0249	4.65	0.74; 8.70	0.0195	0.4531
CD66+ (neutrophils)	5.48	0.68; 10.51	0.0257	0.53	−2.45; 3.60	0.7305	0.0881
EpCAM+ (epithelium)	8.11	2.22; 14.34	0.0068	0.93	−2.34; 4.31	0.5833	0.0263
CD105+ (endothelium)	5.09	0.51; 9.88	0.0298	0.70	−1.91; 3.38	0.6012	0.0826
CD14+ (macrophages/monocytes)	12.04	5.93; 18.51	0.0001	1.51	−2.05; 5.19	0.4119	0.0032

^a^Δ% = (exp (β*10)-1)*100, percentage increase in EV count for each 10-μg/m^3^ increase in PM_10_ concentration

### Association between ambient PM_10_ levels and EV-miRNAs

#### Profiling

**Fig. 2:**
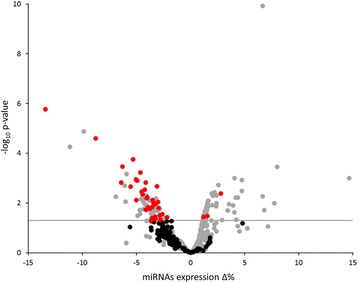
Volcano plot reporting univariate association of Day −1–PM_10_ exposure and all measured miRNAs in EVs. Red dots represent miRNAs chosen for validation. Gray dots represent miRNAs expressed in <50% of subjects. Black dots represent miRNAs with *P* < 0.05 that were expressed in ≥50% of subjects

To assess the occurrence of a signature EV-miRNA response to ambient PM_10_, we used OpenArray technology to screen for EV-miRNA expression among 883 consecutive subjects. After data cleaning (see Additional file 2-Supplemental Methods S3), we examined 545 miRNAs for their association with Day −1–PM_10_ exposure values using multivariable analysis adjusted for age, sex, BMI, smoking status, and apparent temperature. We identified 146 miRNAs that were altered (typically downregulated) in response to Day −1–PM_10_ exposure (Fig. 2).

After FDR adjustment for multiple comparisons (FDR *P* < 0.1), 46 miRNAs were significantly associated with the Day −1–PM_10_ exposure (Additional file 7). The presented results were normalized by the global mean miRNA expression.

As a sensitivity analysis, we applied additional methods for normalization (four more stable miRNAs, U6). This step did not significantly affect our findings. We repeated the analysis of the association between miRNAs and Day −1–PM_10_ by considering exposure estimates from the ARPA CTM (see Methods). This method is characterized by a better spatial resolution than the monitoring stations, but was available only for subjects involved in the screening step. This ancillary analysis did not change our main findings.

#### Validation

Among the miRNAs that were expressed in at least 50% of subjects in the screening subset, we validated the expression of the top 40 differentially expressed miRNAs in 747 consecutive subjects (Additional file 8), using an OpenArray custom panel. Nine of the candidate miRNAs were negatively associated with Day −1–PM_10_ exposure in the validation set (Table 4). Each 10-μg/m^3^ increase in PM_10_ concentration was associated with a 4.2% decrease in hsa-miR-218-5p expression and a 3.51% decrease in hsa-miR-642-5p expression. The remaining seven miRNAs (hsa-miR-99b-5p, hsa-let-7c-5p, hsa-miR-331-3p, hsa-miR-185-5p, hsa-miR-106a-5p, hsa-miR-143-3p, and hsa-miR-652-3p) had decreases in expression ranging from 2.59% to 3.14% (Table 4).

**Table 4:** Association between PM_10_ exposure (Day −1) and levels of validated miRNAs in EVs, after adjustment for age, sex, BMI, smoking status, and apparent temperature

miRNA name	Δ%^a^	95% CI	*P*
hsa-miR-218-5p	−4.20	−1.87; −6.47	0.0005
hsa-miR-99b-5p	−3.14	−0.56; −5.65	0.0173
hsa-let-7c-5p	−2.72	−0.31; −5.08	0.0270
hsa-miR-331-3p	−3.07	−0.26; −5.80	0.0328
hsa-miR-185-5p	−2.78	−0.19; −5.31	0.0359
hsa-miR-642-5p	−3.51	−0.17; −6.74	0.0397
hsa-miR-106a-5p	−2.59	[−0.11; −5.01]	0.0409
hsa-miR-143-3p	−2.75	[−0.04; −5.39]	0.0467
hsa-miR-652-3p	−2.69	[−0.03; −5.29]	0.0478

^a^Δ% = (2 ^(β*10)^-1)*100, percentage increase in EV count for each 10 μg/m^3^ increase in PM_10_ concentration

### Functional analysis of validated miRNAs

To elucidate the mechanisms through which the nine validated EV-miRNAs could mediate the increase of CVD risk produced by PM_10_ exposure, we built and analyzed their miRNA-target prediction networks. For each of the nine miRNAs (hsa-let-7c-5p, hsa-miR-106a-5p, hsa-miR-143-3p, hsa-miR-185-5p, hsa-miR-218-5p, hsa-miR-331-3p, hsa-miR-642-5p, hsa-miR-652-3p, and hsa-miR-99b-5p), we considered miRNAs predicted by at least two of the six evaluated prediction algorithms to be bona fide target genes. In this way, we obtained a network of nine miRNAs and 14,089 predicted target genes. To identify the targets involved in cardiac pathologies, we selected expert-curated gene associations of the main CVDs from the DisGeNET database (v4.0), thereby obtaining a network with 741 genes. The integration of these two networks highlighted the presence of 561 CVD-related genes that were the predicted targets of at least one EV-miRNA (Additional file 9).

**Fig. 3:**
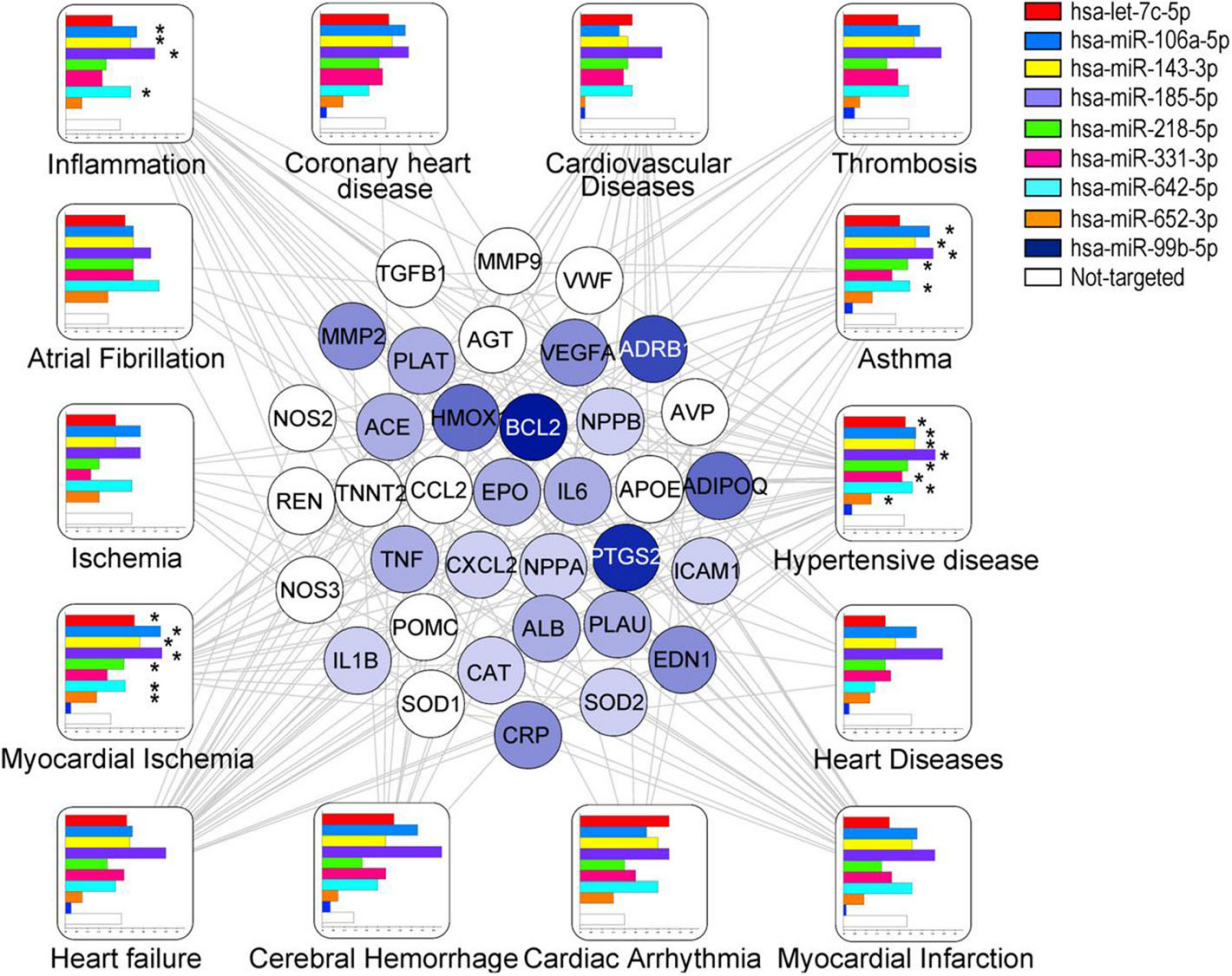
Functional analysis of validated miRNAs. Disease nodes are represented as squares. Bar graph indicates the percentage of predicted targets for each of the nine EV-miRNAs among genes connected to the disease. Rounded nodes show genes involved in at least four pathologies. Increasing blue tone reflects an increase in the number of EV-miRNAs predicted to target each gene. * Significantly enriched EV-miRNAs (FDR < 0.1, Fisher test) among targets of specific CVDs

All of the EV-miRNAs were enriched in targets among the whole list of CVD-related genes with respect to the database (FDR < 0.1, Fisher test), whereas only some EV-miRNAs were significantly enriched among the targets of specific CVDs (FDR < 0.1, Fisher test, Fig. 3). Hsa-miR-99b-5p was not enriched in targets in any of the selected pathologies. Four miRNAs, hsa-miR-106a-5p, hsa-miR-143-3p, hsa-miR-185-5p, and hsa-miR-642-3p, were enriched in targets among genes related to asthma, inflammation, MI, and hypertensive disease. Figure 3 (center) reports the results of network analysis showing genes involved in at least four pathologies. Among them, *BLC2* and *PTGS2* (*COX2*) were the putative targets of six or more EV-miRNAs and, therefore, could be the most relevant mediators for the effects of EV-miRNAs on the CV system.

### Mediation analysis of the influence of PM_10_ exposure on coagulation

**Fig. 4:**
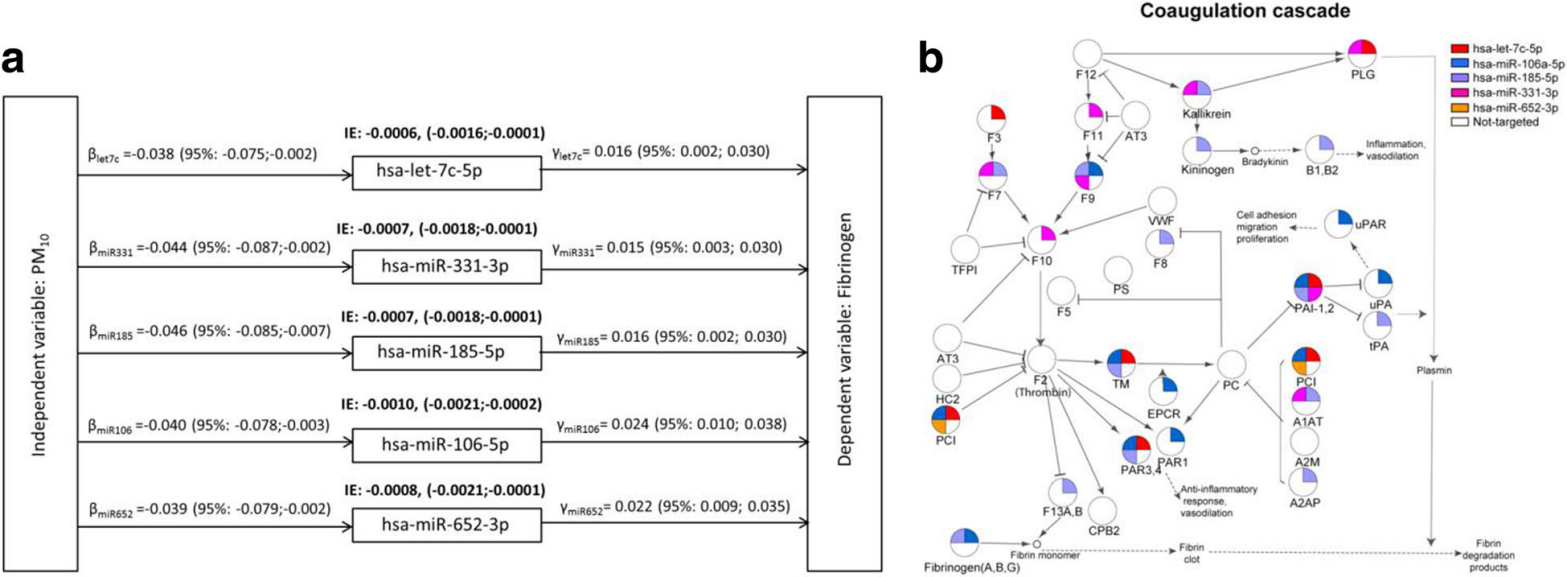
Influence of PM_10_ exposure on coagulation. **a** Investigation of whether the five validated miRNAs mediate the association between PM_10_ concentration (y-axis) and fibrinogen level (x-axis). β, coefficient of the independent variable when regressing the mediator on the independent variable; γ, coefficient of the mediator when regressing the dependent variable on both the independent variable and the mediator. An indirect effect (IE) represents the “mediated effect” through the five validated miRNAs. Estimates correspond to 10-μg/m^3^ increase in PM_10_ concentration. Results from regression models were adjusted for age, sex, BMI, smoking status, and apparent temperature. **b** Coagulation cascade. For each gene reported in the pathway, the possible regulatory role of each miRNA is reported

A 10-μg/m^3^ increase in Day −1–PM_10_ exposure was associated with an average increase in fibrinogen concentration of 1.16% (*P* < 0.001). We performed a mediation analysis to estimate the extent to which the nine validated miRNAs mediated the effects of Day −1–PM_10_ exposure on fibrinogen levels. Mediation analysis suggested that the association between fibrinogen levels and Day −1–PM_10_ exposure may be mediated by five of the nine miRNAs: hsa-let-7c-5p, hsa-miR-331-3p, hsa-miR-185-5p, hsa-miR-106a-5p, and hsa-miR-652-3p (Fig. 4a).

Guided by these measurements, we integrated the information contained in the EV-miRNA target network with the coagulation cascade described in KEGG (Fig. 4b). We found that 27 of the 36 genes included in this cascade could be putative targets of at least one of the five miRNAs whose expression is down-modulated after PM exposure and correlates with fibrinogen increase. In particular, *F10* is predicted to be the target of miR-331-3p, whereas *PAI* is targeted by hsa-let-7c-5p, hsa-miR-331-3p, hsa-miR-185-5p, and hsa-miR-106a-5p. Overall, the pathway seems to be tightly controlled by these miRNAs. Moreover, fibrinogen itself was a predicted target of hsa-miR-106a-5p on its beta chain and hsa-miR-185-5p on its alpha chain.

## Discussion

In the present study, we show that short-term exposure to PM_10_ is associated with an increased release of EVs in a population of overweight/obese subjects [20]. This effect was mainly due to EVs derived from monocyte/macrophage components (CD14+) and from platelets (CD61+). EV miRNA content was examined, and we identified a nine-miRNA signature that was downregulated in response to PM_10_ exposure. Results of an integrated network analysis highlighted the putative role of these miRNAs in CVD. PM_10_ exposure was also significantly associated with elevated fibrinogen levels. Through mediation analysis, we identified five of the nine miRNAs as mediators between PM exposure and fibrinogen levels. Our findings support the hypothesis that EVs play an important role in mediating the health effects of PM exposure, possibly through their miRNA cargo, providing a powerful tool of intercellular communication at the pulmonary and systemic levels.

Cells continuously release EVs, including both exosomes and microvesicles, through which the cells transfer their protein and RNA contents. This process represents an important mechanism for cell-to-cell communication [10]. Exosomes are thought to be constitutively generated, stored, and released from the endosomal system, whereas microvesicles are shed from the plasma membrane in response to specific stimuli, [32] such as PM exposure.

Our investigation focused on EV trafficking and related outcomes in a very large group of overweight or obese subjects. Increased BMI has been reported to cause chronic low-grade inflammation [33]. Surprisingly, the association between PM_10_ exposure and the release of endothelial EVs (as well as most other EV types) was limited to overweight subjects (BMI < 30). Only platelet EVs maintained their association with PM_10_ in obese subjects. These findings are consistent with our preliminary investigation in 25 normal-weight subjects (BMI < 25) and 25 overweight subjects (25 ≤ BMI < 30) who were exposed to PM_10_ and PM_2.5_. The effect of PM exposure on EV release was visible in overweight subjects only [34] and was mainly focused on EV of endothelial origin (CD105+).

As studies of EVs are still in their infancy, it is impossible to draw any definitive conclusion on this complex relationship between PM exposure, EVs, and BMI. One possible scenario is that normal-weight subjects show a low reactivity to PM exposure, as shown in our previous work [34], whereas overweight subjects acquire higher reactivity to PM. All of the EV types investigated are positively modulated by PM exposure, which is suggestive of a complex interplay and cross-talk between different cell types. As BMI further increases, only platelet EVs respond to PM exposure, possibly generating a hypercoagulability status, which is no longer balanced by an efficient response from other cell types. This speculation needs to be further investigated to understand why subjects with increasing BMI behave differently.

In addition to the positive short-term PM_10_ exposure effects we described, we also observed a negative long-term effect of the yearly average of PM_10_ exposure. This negative effect is suggestive of an adaptation of EV response, as the total number of EVs is lower in those subjects who had an higher chronic (i.e. 1 year average) exposure.

To the best of our knowledge, only one study previously reported the effects of PM exposure on EV-packaged miRNAs, in 22 elderly residents of the Boston area [35]. Rodosthenous and colleagues found an association between long-term ambient PM_2.5_ levels and increased levels of EV-miRNAs circulating in serum. Our study complements and supports this first evidence, investigating the role of short-term exposure and finding an overall increase in EV count in response to PM exposure.

We identified nine miRNAs that were downregulated in response to PM_10_ exposure, five of which (let-7c, miR-331, miR-185, miR-106a, and miR-652) had a mediator role in the association between PM exposure and increased fibrinogen levels. Some of these miRNAs have previously been reported to have specific roles in CV processes and diseases [36–42]. In particular, miR-185 may serve as a key regulator of lipid metabolism by targeting the hepatic scavenger receptor class B type I, which plays an important role in selective high-density lipoprotein cholesterol uptake reversing cholesterol transport [36]. MiR-185 is also a posttranscriptional regulator of low-density lipoprotein receptor (LDLR), which mediates endocytosis of LDL particles and helps maintain plasma cholesterol levels [37]. Together with the observed decreased expression of miR-106a, downregulation of miR-106a was found to predict the future risk of fatal MI in healthy individuals [38]. Low serum levels of miR-106a were also reported in patients with acute heart failure [39]. MiR-331 was previously found to regulate the expression of both fibrinogen receptor subunits (α_IIb_ and β_3_) in platelets [40]. Given that we observed a considerable decrease in miR-331 levels, we could speculate that this process might result in enhanced sensitivity to fibrinogen, which, in turn, might determine increased platelet reactivity and play an important role in the hemostatic and thrombotic functions of platelets. The miRNA expression patterns in platelets influence their reactivity, impacting blood coagulation [41]. MiR-218 was shown previously to downregulate fibrinogen synthesis [42]; therefore, the lower levels of expression that we observed in response to PM exposure might partly explain the increased fibrinogen concentration. However, this finding was not supported by the results of our mediation analysis.

This study has several strengths, including a large sample size and careful attention to methodological issues, including all of the steps from sample collection to biomarker measurements. EV analysis itself is very challenging. In the past decade, increasing numbers of researchers have begun investigating EVs; however, those studies have had many limitations related to small sample size, the technical challenges of measuring and characterizing EVs, and the risk of contamination by other nonvesicular entities (e.g., protein complexes). The International Society for Extracellular Vesicles (ISEV) recently published the minimal technical requirements that should be used in EV isolation [43] and biochemical, biophysical, and functional studies [44]. The present study, which includes the largest study population to date, takes all of the ISEV technical requirements into account [43, 44]. NTA and flow cytometry analyses were performed on fresh blood, to avoid hemolysis of the sample and to limit EV modifications [45].

The two exposure assessment methods applied to perform miRNA discovery (monitoring stations vs. CTM) yielded very similar results. This result indicates that neither the potential measurement error of the monitoring stations nor the uncertainty of the CTM estimates consistently contributed to our results. Thus, we are confident that the association we identified between PM_10_ and miRNAs was independent of the selected exposure assessment method.

We used the OpenArray technology, a relatively new method combining the precision of real-time quantitative PCR (RT-qPCR) with the high throughput of microarray analysis. A recent study compared the reproducibility, specificity, sensitivity, and accuracy of the different available methods for miRNA analysis (hybridization methods, RT-qPCR, and miRNAseq), across 12 platforms from nine different vendors [46]. That study found higher overall detection rates and better sensitivity for RT-qPCR versus hybridization platforms. Our use of a screening set of samples and an independent set of validation samples lowered the possibility that our findings were due to chance.

Although normalization is often advocated as a critical step in miRNA analysis, especially when a fixed volume of sample rather than a fixed quantity of RNA is managed, there is no general consensus for the best method of normalization [47]. To address this problem, we applied multiple strategies of normalization in the discovery analysis (global mean, RNU48, RNU6, and the average of the four miRNAs with the lowest SD among subjects), which gave very consistent results. We report in detail the results obtained by global mean normalization [48], because the global mean exhibited a more stable expression pattern according to geNorm and NormFinder. As the global mean approach was intrinsically not suitable for validation analysis, we applied an alternative normalization strategy, combining an endogenous (RNU6) and an exogenous (ath-miR159a) control miRNA, as suggested by Schwarzenbach and colleagues [49]. According to the authors, this approach compensates for each difference in miRNA recovery, cDNA synthesis, and quality of samples.

The present study has some limitations. First, although we were able to characterize EV origin by flow cytometry, no reliable technical procedure is available to separate EVs produced by different cell types, limiting the study of miRNAs in EVs as a whole. Second, in the discovery step, 349 miRNAs were associated with PM_10_ exposure. We had to perform a selection process to validate only 40 of them based on statistical criteria. This procedure might have prevented us from identifying other relevant miRNAs related to PM_10_ exposure and coagulation. Third, we used PM_10_ instead of PM_2.5_ as the air pollutant of choice because the PM_10_ dataset was more complete and characterized by a better spatial resolution. However, in the study area, PM_10_ is mainly constituted by fine particles, and PM_2.5_ represents 58–94% of PM_10_ [50]. Another possible constraint is the lack of personal exposure monitoring, due to the large study sample. This limitation also hampered the possibility of accounting for indoor air pollution [51]. Nonetheless, in the abovementioned paper by Bonzini et al., we compared PM_10_ and PM_2.5_ levels measured by ambient monitoring stations with those measured through personal samplers in 51 healthy volunteers, and found the two measures to be significantly correlated (*r* = 0.59, *P* < 0.001). Furthermore, the two pollutants showed a highly significant correlation (*r* = 0.97; *P* < 0.001) [34].

Unfortunately, no repeated measurements have been obtained, which might have been preferential to study acute changes.

## Conclusions

In conclusion, research on EVs opens a new path to the investigation of the adverse health effects of air pollution exposure. EVs have the potential to act both as markers of PM susceptibility and as potential molecular mechanism in the chain of events connecting PM exposure to increased coagulation, which is frequently linked to exposure and CVD development.

## Additional files

Additional file 1: Figure S1. Diagram describing the two-stage, split-sample study design for miRNA analysis. (PDF 307 kb)

Additional file 2: Methods S1. Isolation and Purification of EVs and EV-miRNAs. **Methods S2.** Analysis of EV Integrity for Flow Cytometry. **Methods S3.** Preparation of miRNAs for Screening. **Methods S4.** Preparation of miRNAs for Validation. (PDF 455 kb)

Additional file 3: Figure S2. Transmission electron microscopy analysis, showing examples of MVs isolated from the plasma of a subject enrolled in the SPHERE Study. (PDF 339 kb)

Additional file 4: Figure S3. Descriptive analysis of PM_10_ concentrations registered on different days from recruitment. (PDF 405 kb)

Additional file 5: Figure S4. NTA analysis. Histograms report EV mode and mean size distributions across samples from subjects in the SPHERE study. (PDF 357 kb)

Additional file 6: Table S1. EVs count and characterization by NTA and Flow cytometry. Variables are expressed as minimum, first quartile, median, third quartile, maximum. (PDF 343 kb)

Additional file 7: Table S2. Screening of miRNA expression levels. Association between Day −1–PM10 exposure levels and EV-miRNA levels measured by OpenArray. (PDF 1099 kb)

Additional file 8: Table S3. Validation of the top-40 differentially expressed miRNAs. Association between Day −1–PM10 exposure levels and EV-miRNAs measured by OpenArray. (PDF 554 kb)

Additional file 9: Table S4. List of the genes involved in CVDs according to DisGeNET database. For each gene all the related diseases and the putative EV-MiRNAs targeting it are indicated both as list and as number of occurrences. (PDF 1206 kb)

## Abbreviations

COW: Center for Obesity and Work
CTM: Chemical transport model
CVD: Cardiovascular disease
EV-miRNAs: EV-associated miRNAs
EVs: Extracellular vesicles
FDR: Benjamini-Hochberg False Discovery Rate
MI: Myocardial ischemia
miRNAs: microRNAs
NTA: Nanoparticle tracking analysis
PM: Particulate matter
