# Supplementary material for: Extracellular vesicle-packaged miRNA release after short-term exposure to particulate matter is associated with increased coagulation
Source: Part Fibre Toxicol. 2017 Aug 24;14:32. doi: 10.1186/s12989-017-0214-4 (PMC5594543; doi:10.1186/s12989-017-0214-4)

Additional file 1. Supplemental figure 1

Diagram describing the two-stage, split-sample study design for miRNA analysis.

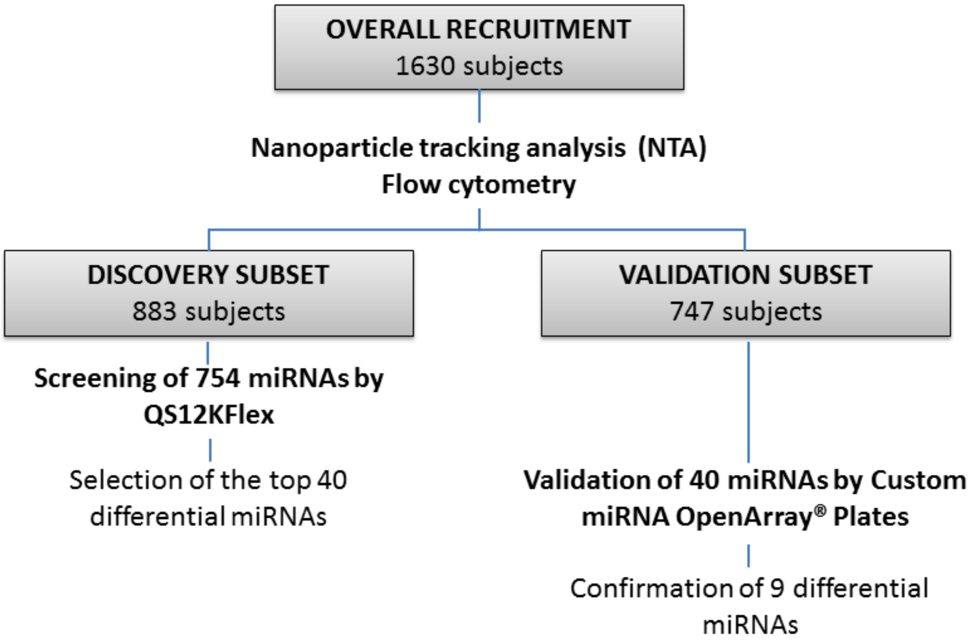

Supplement: Supplementary file 1 — Diagram describing the two-stage, split-sample study design for miRNA analysis. (PDF 307 kb) [file 12989_2017_214_MOESM1_ESM.pdf]
