# Supplementary material for: Extracellular vesicle-packaged miRNA release after short-term exposure to particulate matter is associated with increased coagulation
Source: Part Fibre Toxicol. 2017 Aug 24;14:32. doi: 10.1186/s12989-017-0214-4 (PMC5594543; doi:10.1186/s12989-017-0214-4)
Supplement: Supplementary file 2 — Isolation and Purification of EVs and EV-miRNAs. Methods S2. Analysis of EV Integrity for Flow Cytometry. Methods S3. Preparation of miRNAs for Screening. Methods S4. Preparation of miRNAs for Validation. (PDF 455 kb) [file 12989_2017_214_MOESM2_ESM.pdf]

Additional file 2. Supplemental Methods

Supplemental Methods S1: Isolation and Purification of EVs and EV-miRNAs

Blood was centrifuged at 1200 × *g* for 15 min at room temperature to obtain platelet-free plasma, which was subsequently centrifuged at 1000, 2000, and 3000 × *g* for 15 min at 4 °C. The pellet was discarded to remove cell debris.

To prepare EV pellet, 1.5 ml of fresh plasma was transferred to a 13.5 ml polypropylene ultracentrifuge tube (Beckman Coulter), which was filled with PBS. PBS was filtered through a 0.10 µm pore-size polyethersulfone filter (StericupRVP, Merck Millipore) to minimize the background contribution of interfering particles. Plasma was ultracentrifuged (Beckman Coulter Optima-MAX-XP) at 110,000 × *g* for 75 min at 4 °C, to obtain an EV-rich pellet. The pellet was resuspended in 500 µl of triple-filtered PBS (0.10 µm pore-size).

To prepare EV pellet for miRNA extraction, 1.5 ml of fresh plasma was transferred to a 10.4 ml polycarbonate ultracentrifuge tube (Beckman Coulter), which was filled with PBS. Plasma was ultracentrifuged (BeckmanCoulter Optima-MAX-XP) at 110,000 × *g* for 75 min at 4 °C and decanted. The EV pellet was kept at -80 °C until use.

MiRNAs were isolated from frozen EV pellets by using the miRNeasy Kit and RNeasy CleanUp Kit (Qiagen). Briefly, 700 µl of QIAzol Lysis Reagent was added to each ultracentrifuge tube to lyse membranized particles. MiRNA extraction was performed according to the manufacturer's protocol. The final purified miRNA-enriched RNA was eluted in 20 µl of RNAase-free water and stored at -80 °C until further use.

Supplemental Methods S2: Analysis of EV Integrity for Flow Cytometry

To analyze EV integrity, 60 µl aliquots were stained with 0.02 µM 5(6)-carboxyfluorescein diacetate N-succinimidyl ester (CFSE) at 37 °C for 20 min in the dark. Each aliquot of CFSE-stained sample was incubated with a specific antibody (Miltenyi Biotec): CD14-APC (clone TÜK4), CD105-

APC (clone 43A4E1), CD326 (EpCAM)-APC (clone HEA-125), CD66abce-FITC (clone TET2), and CD61-APC (clone Y2/51). Before use, each antibody was centrifuged at 17,000 × *g* for 30 min at 4 °C to eliminate aggregates. The stained PBS control sample was used to detect the autofluorescence of the antibody.

Supplemental Methods S3: Preparation of miRNAs for Screening

Reverse transcription (RT) was performed by using Megaplex™ RT Primers, Pool A v2.1 and Pool B v3.0, with the TaqMan® Micro RNA Reverse Transcriptase Kit (Life Technologies, Foster City, CA). Two distinct reactions were performed, to cover RT of 754 target miRNAs (16 replicates of four internal controls: ath-miR159a, RNU48, RNU44, and U6). Each reaction included: 0.75 µl of Megaplex RT Primers Pool A or Pool B, 0.15 µl of dNTPs (100 mM), 0.75 µl of 10× RT Buffer, 0.90 µl of $MgCl_2$ (25 mM), 0.1 µl of RNase Inhibitor (20 U/µl), 1.5 µl of MultiScribe™ Reverse Transcriptase (50 U/µl), and 3.3 µl of miRNAs. After incubation on ice for 5 min, the mixture was subjected to the following thermal protocol in a C1000 Thermal Cycler (Biorad, Hercules, CA): 40 cycles of 16 °C for 2 min, 42 °C for 1 min, and 50 °C for 1 s, plus one cycle at 85 °C for 5 min. The cDNA samples were stored at -20 °C until use.

Each cDNA requiring preamplification was loaded onto a 96-well plate in accordance with the protocol provided by the manufacturer (Application Note 2011, Life Technologies). 7.5 µl of each reverse-transcribed miRNA was combined with the following reaction mix: 20 µl of TaqMan® PreAmp Master Mix (2×), 8.5 µl of nuclease-free water, and 4 µl of Megaplex™ PreAmp Primers Pool A or B (10×). Thermal conditions for the preamplification reaction were as follows: 95 °C for 10 min, 55 °C for 2 min, 72 °C for 2 min, 16 cycles of 95 °C for 15 s and 60 °C for 4 min, and 99.9 °C for 10 min. Preamplified samples were stored at 4 °C until expression analysis with the OpenArray® System.

Each preamplified cDNA was diluted 1:20 with nuclease-free water. TaqMan Open Array® Real Time PCR Master Mix (2×) was added in a 1:1 volume ratio. Then, 7 µl of the reaction RT-PCR mix was aliquoted with the MicroLab STAR Let instrument (Hamilton Robotics, Birmingham, UK) into eight wells of a 384-well OpenArray® plate. The reaction mix was loaded from the 384-well plate into a TaqMan™ OpenArray® Human miRNA Panel, with QuantStudio™ AccuFill System Robot (Life Technologies, Foster City, CA). The mixture was analyzed with the QuantStudio™ 12K Flex Real-Time PCR System with the OpenArray® Platform (QS12KFlex), according to the manufacturer's instructions.

Supplemental Methods S4: Preparation of miRNAs for Validation

The top-40 miRNAs were validated in Custom miRNA OpenArray® Plates in triplicate. RT was performed by using the Custom Primers Pool and TaqMan® Micro RNA Reverse Transcriptase Kit (Life Technologies). Each reaction included: 3 µl of RT Primers Pool, 0.15 µl of dNTPs (100 mM), 0.75 µl 10× RT Buffer, 0.09 µl of RNases Inhibitor (20 U/µl), 1.5 µl of MultiScribe™ Reverse Transcriptase (50 U/µl), and 2.01 µl of miRNA. After incubation on ice for 5 min, the mixture was subjected to the following thermal protocol in a C1000 Thermal Cycler (Biorad): 16 °C for 30 min, 42 °C for 30 min, and 85 °C for 5 min. The cDNA samples were stored at -20 °C until further use.

Each cDNA was preamplified in a 96-well plate, in a reaction mix that included: 12.5 µl of TaqMan® PreAmp Master Mix (2×), 10 µl of nuclease-free water, 10 µl of PreAmp Custom Primers Pool, and 7.5 µl of reverse-transcribed miRNA. Preamplification, loading, and analyzing conditions were the same as those of the screening phase, with the exception that 6 µl of reaction RT-PCR mix was aliquoted with the MicroLab STAR Let robot (Hamilton Robotics, Birmingham, UK) into three wells of a 384-well OpenArray® plate.
