# Supplementary material for: Extracellular vesicle-packaged miRNA release after short-term exposure to particulate matter is associated with increased coagulation
Source: Part Fibre Toxicol. 2017 Aug 24;14:32. doi: 10.1186/s12989-017-0214-4 (PMC5594543; doi:10.1186/s12989-017-0214-4)

Additional file 3. Supplementary Figure S2: Transmission electron microscopy analysis, showing examples of MVs isolated from the plasma of a subject enrolled in the SPHERE Study.

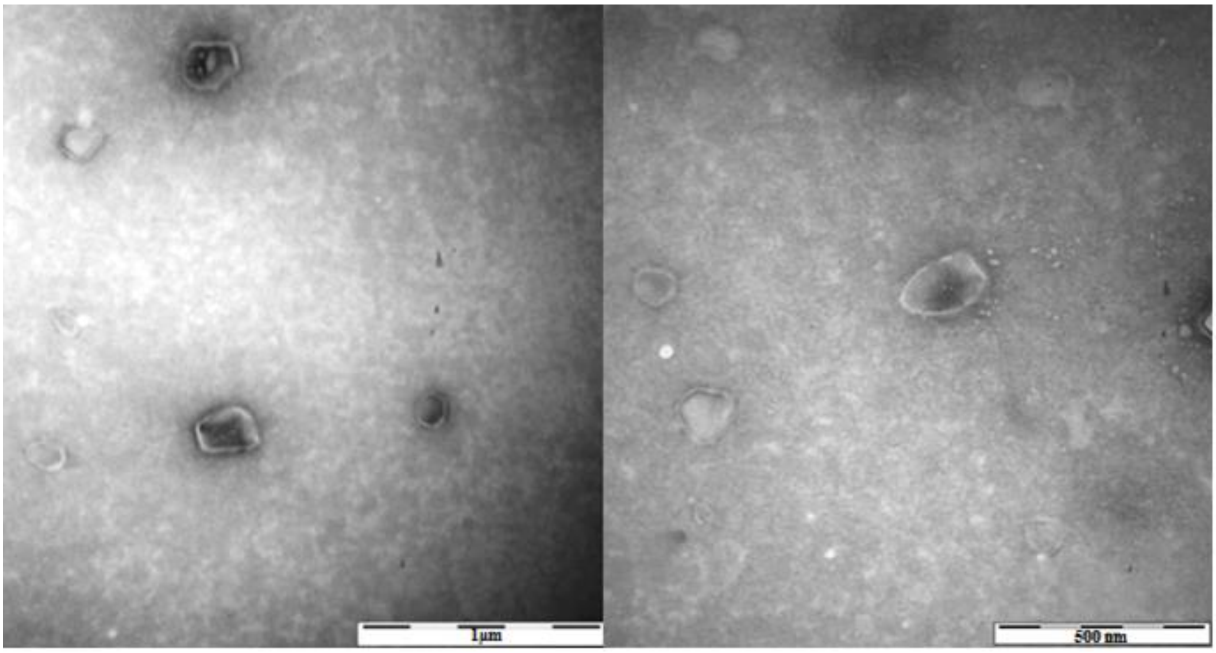

Supplement: Supplementary file 3 — Transmission electron microscopy analysis, showing examples of MVs isolated from the plasma of a subject enrolled in the SPHERE Study. (PDF 339 kb) [file 12989_2017_214_MOESM3_ESM.pdf]
