# Supplementary material for: Extracellular vesicle-packaged miRNA release after short-term exposure to particulate matter is associated with increased coagulation
Source: Part Fibre Toxicol. 2017 Aug 24;14:32. doi: 10.1186/s12989-017-0214-4 (PMC5594543; doi:10.1186/s12989-017-0214-4)

Additional file 4. Supplementary Figure S3:Descriptive analysis of PM_{10} concentrations registered on different days from recruitment.

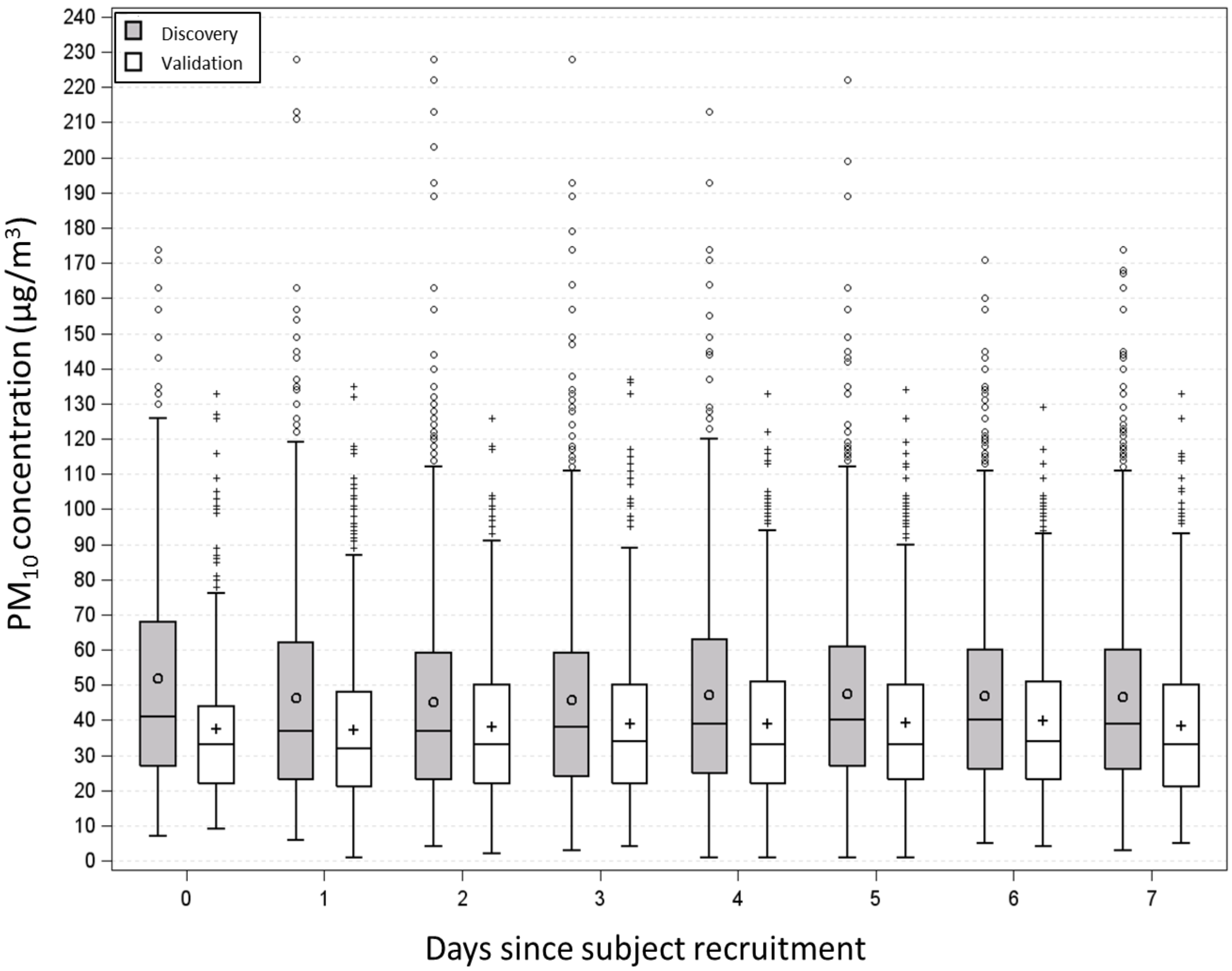

Supplement: Supplementary file 4 — Descriptive analysis of PM10 concentrations registered on different days from recruitment. (PDF 405 kb) [file 12989_2017_214_MOESM4_ESM.pdf]
