# Supplementary material for: Extracellular vesicle-packaged miRNA release after short-term exposure to particulate matter is associated with increased coagulation
Source: Part Fibre Toxicol. 2017 Aug 24;14:32. doi: 10.1186/s12989-017-0214-4 (PMC5594543; doi:10.1186/s12989-017-0214-4)

Additional file 5. Supplementary Figure S4: NTA analysis. Histograms report EV mode and mean size distributions across samples from subjects in the SPHERE study.

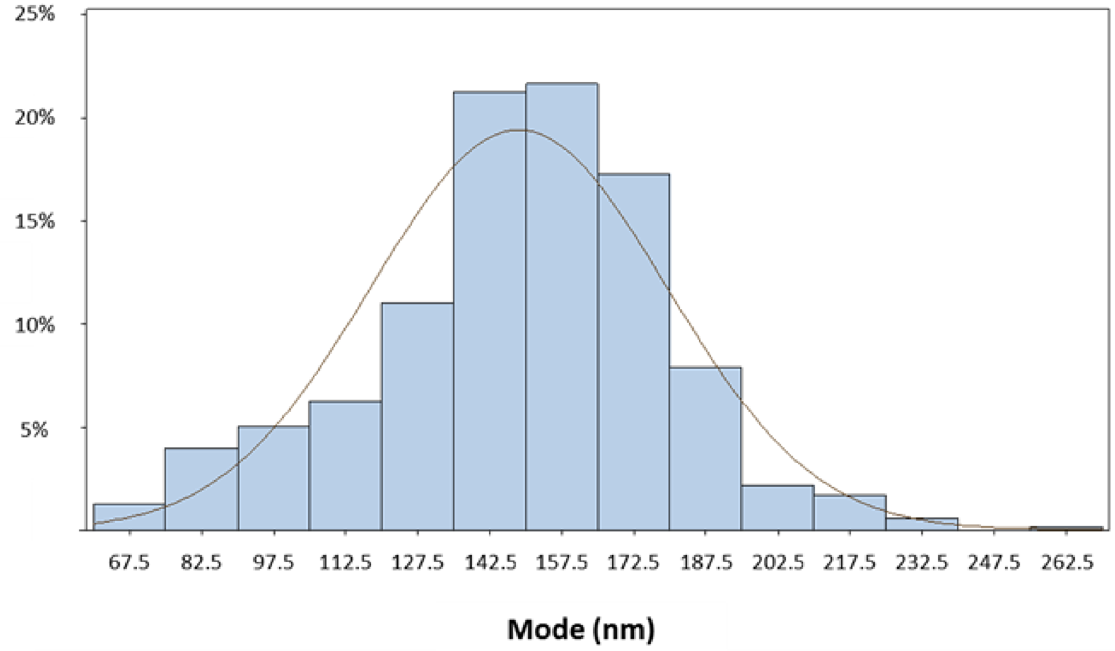

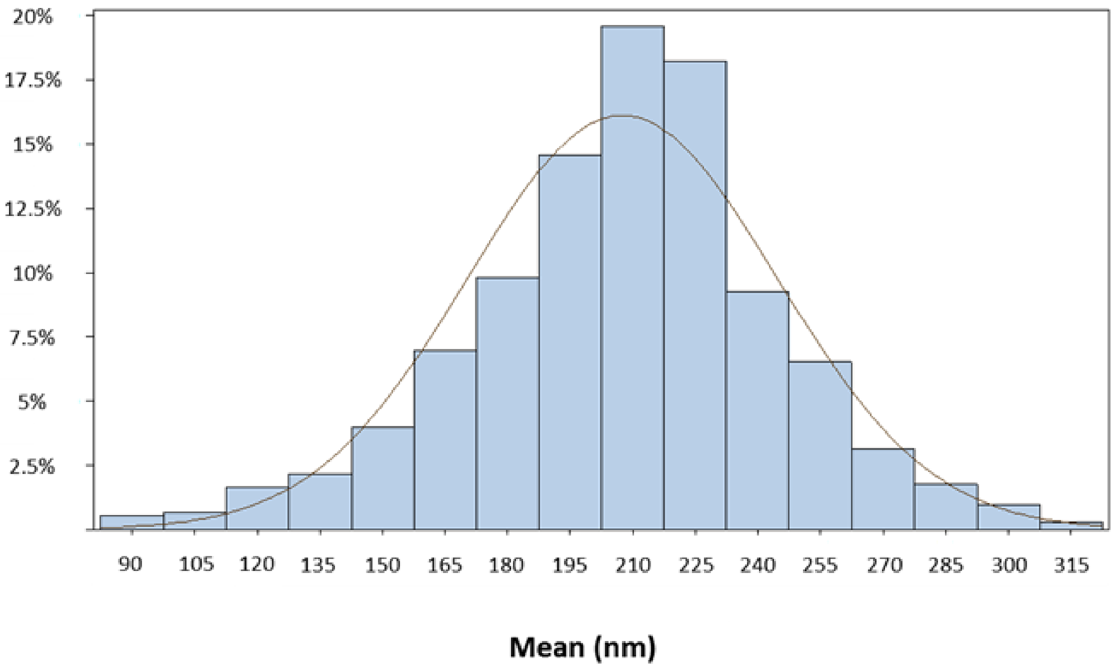

Supplement: Supplementary file 5 — NTA analysis. Histograms report EV mode and mean size distributions across samples from subjects in the SPHERE study. (PDF 357 kb) [file 12989_2017_214_MOESM5_ESM.pdf]
