# Supplementary material for: Extracellular vesicle-packaged miRNA release after short-term exposure to particulate matter is associated with increased coagulation
Source: Part Fibre Toxicol. 2017 Aug 24;14:32. doi: 10.1186/s12989-017-0214-4 (PMC5594543; doi:10.1186/s12989-017-0214-4)
Supplement: Supplementary file 6 — EVs count and characterization by NTA and Flow cytometry. Variables are expressed as minimum, first quartile, median, third quartile, maximum. (PDF 343 kb) [file 12989_2017_214_MOESM6_ESM.pdf]

Additional file 6. Supplementary Table S1: EVs count and characterization by NTA and Flow cytometry. Variables are expressed as minimum, first quartile, median, third quartile, maximum.

		Min	Q1	Median	Q3	Max
**NTA analysis**	**Total EVs, 10^3/µl**	247	1642	2360	3213	15672
**Flow cytometry analysis**	**CD61+ (platelets), 1/µl**	1	38	82	157	2351
	**CD66+ (neutrophils), 1/µl**	1	9	16	28	224
	**EpCAM+ (epithelium), 1/µl**	1	12	12	23	147
	**CD105+ (endothelium), 1/µl**	1	9	9	15	53
	**CD14+ (macrophages/monocytes) , 1/µl**	1	15	15	29	821
