# Supplementary material for: Extracellular vesicle-packaged miRNA release after short-term exposure to particulate matter is associated with increased coagulation
Source: Part Fibre Toxicol. 2017 Aug 24;14:32. doi: 10.1186/s12989-017-0214-4 (PMC5594543; doi:10.1186/s12989-017-0214-4)
Supplement: Supplementary file 7 — Screening of miRNA expression levels. Association between Day −1–PM10 exposure levels and EV-miRNA levels measured by OpenArray. (PDF 1099 kb) [file 12989_2017_214_MOESM7_ESM.pdf]

**Additional file 7.** Supplementary Table S2: Screening of miRNA expression levels. Association between Day -1–$PM_{10}$ exposure levels and EV-miRNA levels measured by OpenArray.

| **miRNA name** | **Quant Studio Assay ID** | **Δ (%) (95% CI)** | **Raw *P*** | **FDR *P*** | **>50%** | **Chosen for validation (Y/N)** |
|---|---|---|---|---|---|---|
| hsa-let-7a-5p | 000377 | -1.40 (5.63; -7.96) | 0.6882 | 0.7963 | 1 | N |
| hsa-let-7b-3p | 002404 | 0.83 (2.53; -0.85) | 0.3355 | 0.5606 | 0 | N |
| hsa-let-7b-5p | 002619 | 3.56 (6.40; 0.80) | 0.0111 | 0.1081 | 0 | N |
| hsa-let-7c#* | 002405 | 0.75 (2.46; -0.94) | 0.3883 | 0.6052 | 0 | N |
| hsa-let-7c-5p | 000379 | -5.05 (-2.05; -7.95) | 0.0011 | 0.0417 | 1 | Y |
| hsa-let-7d-3p | 001178 | 0.60 (2.39; -1.16) | 0.5078 | 0.6748 | 0 | N |
| hsa-let-7d-5p | 002283 | -5.01 (-1.35; -8.52) | 0.0077 | 0.0949 | 1 | Y |
| hsa-let-7e-5p | 002406 | -1.33 (3.71; -6.13) | 0.5969 | 0.7344 | 1 | N |
| hsa-let-7f-5p | 000382 | 0.95 (7.31; -5.03) | 0.7619 | 0.8428 | 0 | N |
| hsa-let-7f-1-3p | 002417 | 0.86 (2.43; -0.68) | 0.2752 | 0.5207 | 0 | N |
| hsa-let-7f-2-3p | 002418 | 1.21 (2.35; 0.08) | 0.0361 | 0.1659 | 0 | N |
| hsa-let-7g-3p | 002118 | 0.44 (2.04; -1.15) | 0.5907 | 0.7333 | 0 | N |
| hsa-let-7g-5p | 002282 | -3.52 (-0.71; -6.25) | 0.0144 | 0.1187 | 1 | Y |
| hsa-let-7i-3p | 002172 | 0.11 (2.11; -1.85) | 0.9116 | 0.9451 | 0 | N |
| hsa-miR-1-3p | 002222 | 1.30 (3.88; -1.23) | 0.3165 | 0.5500 | 0 | N |
| hsa-miR-100-5p | 000437 | 0.63 (5.78; -4.26) | 0.8033 | 0.8662 | 1 | N |
| hsa-miR-101-3p | 002253 | -3.37 (-0.31; -6.34) | 0.0312 | 0.1659 | 0 | N |
| hsa-miR-103a-3p | 000439 | -2.19 (1.19; -5.46) | 0.2008 | 0.4226 | 1 | N |
| hsa-miR-106a-3p | 002170 | 0.80 (2.56; -0.92) | 0.3634 | 0.5912 | 0 | N |
| hsa-miR-106a-5p | 002169 | -3.08 (-1.13; -4.99) | 0.0021 | 0.0527 | 1 | Y |
| hsa-miR-106b-3p | 002380 | 4.39 (7.64; 1.24) | 0.0061 | 0.0877 | 0 | N |
| hsa-miR-106b-5p | 000442 | -2.19 (0.05; -4.39) | 0.0556 | 0.1955 | 1 | N |
| hsa-miR-107 | 000443 | -2.79 (1.03; -6.47) | 0.1501 | 0.3636 | 1 | N |

| miRNA name | Quant Studio Assay ID | Δ (%) (95% CI) | Raw *P* | FDR *P* | >50% | Chosen for validation (Y/N) |
|---|---|---|---|---|---|---|
| hsa-miR-10a-5p | 000387 | -2.58 (0.32; -5.40) | 0.0801 | 0.2413 | 1 | N |
| hsa-miR-10b-3p | 002315 | -1.76 (1.56; -4.96) | 0.2945 | 0.5408 | 1 | N |
| hsa-miR-10b-5p | 002218 | -0.41 (3.85; -4.49) | 0.8476 | 0.8978 | 1 | N |
| hsa-miR-1178-3p | 002777 | 0.74 (2.57; -1.07) | 0.4267 | 0.6076 | 0 | N |
| hsa-miR-1179 | 002776 | 0.45 (2.04; -1.11) | 0.5705 | 0.7198 | 0 | N |
| hsa-miR-1180-3p | 002847 | 1.08 (3.05; -0.86) | 0.2765 | 0.5214 | 0 | N |
| hsa-miR-1183 | 002841 | 6.66 (8.75; 4.61) | 0.0000 | 0.0000 | 0 | N |
| hsa-miR-1184 | 002842 | 0.76 (2.45; -0.91) | 0.3731 | 0.5981 | 0 | N |
| hsa-miR-1200 | 002829 | 0.88 (3.01; -1.22) | 0.4144 | 0.6074 | 0 | N |
| hsa-miR-1204 | 002872 | 0.74 (2.40; -0.88) | 0.3718 | 0.5978 | 0 | N |
| hsa-miR-1208 | 002880 | 2.00 (3.59; 0.44) | 0.0120 | 0.1093 | 0 | N |
| hsa-miR-122-3p | 002130 | 0.59 (2.14; -0.93) | 0.4488 | 0.6256 | 0 | N |
| hsa-miR-122-5p | 002245 | -5.91 (-2.55; -9.15) | 0.0007 | 0.0372 | 0 | N |
| hsa-miR-1226-5p | 002758 | 1.16 (3.01; -0.65) | 0.2094 | 0.4290 | 0 | N |
| hsa-miR-1227-3p | 002769 | -0.93 (1.56; -3.35) | 0.4618 | 0.6388 | 0 | N |
| hsa-miR-1228-5p | 002763 | 0.55 (2.64; -1.49) | 0.5970 | 0.7344 | 0 | N |
| hsa-miR-1233-3p | 002768 | 1.05 (2.23; -0.12) | 0.0778 | 0.2356 | 0 | N |
| hsa-miR-1243 | 002854 | -5.95 (8.73; -18.65) | 0.4070 | 0.6074 | 0 | N |
| hsa-miR-1244 | 002791 | 0.60 (2.40; -1.16) | 0.5046 | 0.6741 | 0 | N |
| hsa-miR-1247-5p | 002893 | 1.20 (2.35; 0.07) | 0.0377 | 0.1659 | 0 | N |
| hsa-miR-1248 | 002870 | 0.75 (2.46; -0.94) | 0.3885 | 0.6052 | 0 | N |
| hsa-miR-1249-3p | 002868 | 0.82 (2.06; -0.42) | 0.1952 | 0.4155 | 0 | N |
| hsa-miR-124-3p | 001182 | 0.34 (2.11; -1.40) | 0.7025 | 0.8095 | 0 | N |
| hsa-miR-1252-5p | 002860 | 0.15 (2.62; -2.25) | 0.9008 | 0.9387 | 0 | N |

| miRNA name | Quant Studio<br><br>Assay ID | Δ (%) (95% CI) | Raw *P* | FDR *P* | >50% | Chosen for validation (Y/N) |
|---|---|---|---|---|---|---|
| hsa-miR-1254 | 002818 | 0.61 (2.14; -0.89) | 0.4271 | 0.6076 | 0 | N |
| hsa-miR-1255b-5p | 002801 | -0.73 (1.43; -2.83) | 0.5059 | 0.6741 | 0 | N |
| hsa-miR-125a-3p | 002199 | -0.29 (1.70; -2.25) | 0.7711 | 0.8473 | 0 | N |
| hsa-miR-125a-5p | 002198 | -1.27 (2.82; -5.20) | 0.5365 | 0.6912 | 1 | N |
| hsa-miR-125b-5p | 000449 | -4.64 (-2.02; -7.18) | 0.0006 | 0.0365 | 1 | Y |
| hsa-miR-125b-1-3p | 002378 | -1.23 (1.27; -3.67) | 0.3313 | 0.5561 | 0 | N |
| hsa-miR-125b-2-3p | 002158 | 1.29 (2.47; 0.11) | 0.0314 | 0.1659 | 0 | N |
| hsa-miR-126-5p | 000451 | -2.36 (1.53; -6.10) | 0.2301 | 0.4610 | 1 | N |
| hsa-miR-126-3p | 002228 | -2.19 (1.69; -5.92) | 0.2641 | 0.5069 | 1 | N |
| hsa-miR-1260a | 002896 | 0.71 (5.10; -3.49) | 0.7438 | 0.8306 | 0 | N |
| hsa-miR-1262 | 002852 | 0.96 (2.48; -0.53) | 0.2079 | 0.4275 | 0 | N |
| hsa-miR-1265 | 002790 | 1.19 (2.34; 0.06) | 0.0390 | 0.1659 | 0 | N |
| hsa-miR-1267 | 002885 | 1.03 (2.21; -0.13) | 0.0830 | 0.2472 | 0 | N |
| hsa-miR-1269a | 002789 | 1.30 (2.47; 0.14) | 0.0284 | 0.1612 | 0 | N |
| hsa-miR-127-3p | 000452 | -2.83 (0.86; -6.38) | 0.1308 | 0.3379 | 1 | N |
| hsa-miR-1270 | 002807 | 1.22 (2.36; 0.08) | 0.0355 | 0.1659 | 0 | N |
| hsa-miR-1271-5p | 002779 | -0.58 (1.70; -2.81) | 0.6158 | 0.7525 | 0 | N |
| hsa-miR-1274A* | 002883 | -0.49 (4.69; -5.42) | 0.8484 | 0.8978 | 1 | N |
| hsa-miR-1274B* | 002884 | -3.01 (-0.49; -5.46) | 0.0198 | 0.1310 | 0 | N |
| hsa-miR-1275 | 002840 | 1.16 (2.89; -0.54) | 0.1802 | 0.4029 | 0 | N |
| hsa-miR-1276 | 002843 | -0.14 (2.64; -2.84) | 0.9215 | 0.9451 | 0 | N |
| hsa-miR-1278 | 002851 | 1.16 (2.93; -0.57) | 0.1899 | 0.4124 | 0 | N |
| hsa-miR-1285-3p | 002822 | 1.19 (2.34; 0.06) | 0.0394 | 0.1659 | 0 | N |
| hsa-miR-128-3p | 002216 | -1.86 (1.58; -5.19) | 0.2850 | 0.5320 | 1 | N |

| miRNA name | Quant Studio Assay ID | Δ (%) (95% CI) | Raw *P* | FDR *P* | >50% | Chosen for validation (Y/N) |
|---|---|---|---|---|---|---|
| hsa-miR-129-1-3p | 002298 | 0.44 (2.04; -1.15) | 0.5907 | 0.7333 | 0 | N |
| hsa-miR-129-2-3p | 001184 | 0.43 (2.67; -1.77) | 0.7047 | 0.8102 | 0 | N |
| hsa-miR-1290 | 002863 | 0.89 (2.42; -0.62) | 0.2492 | 0.4851 | 0 | N |
| hsa-miR-1291 | 002838 | -2.75 (0.09; -5.52) | 0.0577 | 0.1989 | 1 | N |
| hsa-miR-1292-5p | 002824 | 0.84 (2.45; -0.74) | 0.2974 | 0.5420 | 0 | N |
| hsa-miR-1296-5p | 002908 | 1.21 (2.36; 0.07) | 0.0367 | 0.1659 | 0 | N |
| hsa-miR-1300* | 002902 | 1.52 (2.81; 0.25) | 0.0189 | 0.1308 | 0 | N |
| hsa-miR-1301-3p | 002827 | 1.07 (2.22; -0.07) | 0.0663 | 0.2138 | 0 | N |
| hsa-miR-1302 | 002901 | 1.57 (2.79; 0.36) | 0.0111 | 0.1081 | 0 | N |
| hsa-miR-1303 | 002792 | 0.61 (2.14; -0.90) | 0.4314 | 0.6076 | 0 | N |
| hsa-miR-1304-5p | 002874 | 0.79 (2.58; -0.97) | 0.3813 | 0.6040 | 0 | N |
| hsa-miR-1305 | 002867 | 1.16 (2.31; 0.02) | 0.0456 | 0.1775 | 0 | N |
| hsa-miR-130a-3p | 000454 | -4.04 (-1.25; -6.75) | 0.0049 | 0.0837 | 0 | N |
| hsa-miR-130b-5p | 002114 | 1.15 (3.17; -0.84) | 0.2591 | 0.5007 | 0 | N |
| hsa-miR-130b-3p | 000456 | -4.21 (-0.97; -7.35) | 0.0113 | 0.1081 | 0 | N |
| hsa-miR-132-5p | 002132 | 0.49 (2.44; -1.43) | 0.6224 | 0.7572 | 0 | N |
| hsa-miR-132-3p | 000457 | 0.28 (5.28; -4.47) | 0.9091 | 0.9451 | 1 | N |
| hsa-miR-133a-3p | 002246 | 0.22 (4.47; -3.85) | 0.9158 | 0.9451 | 1 | N |
| hsa-miR-133b | 002247 | 1.51 (3.21; -0.16) | 0.0762 | 0.2334 | 0 | N |
| hsa-miR-134-5p | 001186 | -1.16 (2.26; -4.48) | 0.5003 | 0.6716 | 1 | N |
| hsa-miR-135a-5p | 000460 | -4.35 (-1.10; -7.49) | 0.0091 | 0.1013 | 0 | N |
| hsa-miR-135b-3p | 002159 | 0.73 (2.16; -0.69) | 0.3172 | 0.5500 | 0 | N |
| hsa-miR-135b-5p | 002261 | -1.36 (1.05; -3.71) | 0.2656 | 0.5079 | 0 | N |
| hsa-miR-136-3p | 002100 | -2.49 (0.83; -5.69) | 0.1394 | 0.3472 | 1 | N |

| miRNA name | Quant Studio Assay ID | Δ (%) (95% CI) | Raw *P* | FDR *P* | >50% | Chosen for validation (Y/N) |
|---|---|---|---|---|---|---|
| hsa-miR-136-5p | 000592 | -2.46 (2.86; -7.51) | 0.3575 | 0.5869 | 0 | N |
| hsa-miR-137 | 001129 | 1.26 (2.41; 0.11) | 0.0313 | 0.1659 | 0 | N |
| hsa-miR-138-5p | 002284 | -1.59 (1.68; -4.75) | 0.3363 | 0.5606 | 0 | N |
| hsa-miR-139-3p | 002313 | 4.51 (10.78; -1.40) | 0.1372 | 0.3468 | 0 | N |
| hsa-miR-139-5p | 002289 | -1.94 (0.83; -4.63) | 0.1676 | 0.3838 | 1 | N |
| hsa-miR-140-5p | 001187 | -2.50 (0.41; -5.33) | 0.0914 | 0.2589 | 1 | N |
| hsa-miR-140-3p | 002234 | -2.63 (0.69; -5.85) | 0.1194 | 0.3143 | 1 | N |
| hsa-miR-141-3p | 000463 | 2.36 (4.89; -0.11) | 0.0617 | 0.2060 | 0 | N |
| hsa-miR-142-3p | 000464 | -2.89 (-0.17; -5.53) | 0.0378 | 0.1659 | 0 | N |
| hsa-miR-142-5p | 002248 | -1.97 (1.29; -5.12) | 0.2327 | 0.4628 | 1 | N |
| hsa-miR-143-3p | 002249 | -4.43 (-1.40; -7.37) | 0.0045 | 0.0814 | 1 | Y |
| hsa-miR-144-5p | 002148 | -1.41 (2.88; -5.52) | 0.5133 | 0.6764 | 1 | N |
| hsa-miR-144-3p | 002676 | 1.40 (2.78; 0.03) | 0.0451 | 0.1768 | 0 | N |
| hsa-miR-145-3p | 002149 | -0.10 (1.82; -2.00) | 0.9151 | 0.9451 | 0 | N |
| hsa-miR-145-5p | 002278 | 0.41 (5.36; -4.30) | 0.8661 | 0.9113 | 1 | N |
| hsa-miR-146a-5p | 000468 | -0.12 (3.76; -3.85) | 0.9512 | 0.9690 | 1 | N |
| hsa-miR-146b-5p | 001097 | -0.56 (2.08; -3.15) | 0.6725 | 0.7898 | 1 | N |
| hsa-miR-146b-3p | 002361 | -0.07 (2.44; -2.51) | 0.9580 | 0.9722 | 0 | N |
| hsa-miR-147a | 000469 | 0.31 (2.04; -1.40) | 0.7238 | 0.8201 | 0 | N |
| hsa-miR-147b | 002262 | 0.94 (2.64; -0.73) | 0.2731 | 0.5205 | 0 | N |
| hsa-miR-148a-3p | 000470 | -13.40 (-8.17; -18.33) | 0.0000 | 0.0005 | 1 | Y |
| hsa-miR-148b-5p | 002160 | -2.33 (0.23; -4.82) | 0.0737 | 0.2306 | 0 | N |
| hsa-miR-148b-3p | 000471 | -4.15 (-0.71; -7.46) | 0.0184 | 0.1308 | 1 | Y |
| hsa-miR-149-5p | 002255 | 1.12 (2.31; -0.05) | 0.0609 | 0.2050 | 0 | N |

| miRNA name | Quant Studio<br><br>Assay ID | Δ (%) (95% CI) | Raw *P* | FDR *P* | >50% | Chosen for validation (Y/N) |
|---|---|---|---|---|---|---|
| hsa-miR-150-5p | 000473 | 0.59 (4.82; -3.47) | 0.7786 | 0.8512 | 1 | N |
| hsa-miR-151a-3p | 002254 | -2.86 (3.01; -8.40) | 0.3316 | 0.5561 | 1 | N |
| hsa-miR-151a-5p | 002642 | -0.71 (2.52; -3.85) | 0.6616 | 0.7821 | 1 | N |
| hsa-miR-152-3p | 000475 | -4.13 (-1.60; -6.59) | 0.0015 | 0.0434 | 1 | Y |
| hsa-miR-153-3p | 001191 | 0.67 (2.18; -0.82) | 0.3792 | 0.6026 | 0 | N |
| hsa-miR-154-3p | 000478 | 0.38 (2.57; -1.76) | 0.7268 | 0.8201 | 0 | N |
| hsa-miR-154-5p | 000477 | 0.69 (2.11; -0.71) | 0.3374 | 0.5606 | 0 | N |
| hsa-miR-155-5p | 002623 | 0.93 (6.84; -4.65) | 0.7485 | 0.8325 | 1 | N |
| hsa-miR-15a-3p | 002419 | 0.68 (3.14; -1.71) | 0.5781 | 0.7277 | 0 | N |
| hsa-miR-15a-5p | 000389 | -2.82 (1.89; -7.31) | 0.2361 | 0.4656 | 1 | N |
| hsa-miR-15b-3p | 002173 | 2.91 (5.89; 0.02) | 0.0484 | 0.1830 | 0 | N |
| hsa-miR-15b-5p | 000390 | -3.37 (0.13; -6.74) | 0.0590 | 0.2021 | 1 | N |
| hsa-miR-16-5p | 000391 | -1.32 (1.15; -3.73) | 0.2927 | 0.5407 | 1 | N |
| hsa-miR-16-1-3p | 002420 | -1.09 (1.55; -3.67) | 0.4133 | 0.6074 | 1 | N |
| hsa-miR-17-3p | 002421 | 1.29 (2.56; 0.04) | 0.0429 | 0.1720 | 0 | N |
| hsa-miR-17-5p | 002308 | -1.94 (0.63; -4.44) | 0.1374 | 0.3468 | 1 | N |
| hsa-miR-181a-5p | 000480 | -3.64 (0.70; -7.80) | 0.0991 | 0.2714 | 1 | N |
| hsa-miR-181a-2-3p | 002317 | -3.28 (-0.20; -6.27) | 0.0370 | 0.1659 | 1 | Y |
| hsa-miR-181c-3p | 002333 | 1.15 (2.93; -0.60) | 0.1978 | 0.4178 | 0 | N |
| hsa-miR-181c-5p | 000482 | -1.42 (1.97; -4.70) | 0.4068 | 0.6074 | 1 | N |
| hsa-miR-182-5p | 002334 | -1.65 (1.42; -4.64) | 0.2882 | 0.5361 | 0 | N |
| hsa-miR-183-3p | 002270 | 3.46 (6.47; 0.54) | 0.0200 | 0.1310 | 0 | N |
| hsa-miR-183-5p | 002269 | 1.62 (2.93; 0.32) | 0.0141 | 0.1187 | 0 | N |
| hsa-miR-184 | 000485 | -5.53 (-2.03; -8.90) | 0.0022 | 0.0527 | 1 | Y |

| miRNA name | Quant Studio<br><br>Assay ID | Δ (%) (95% CI) | Raw *P* | FDR *P* | >50% | Chosen for validation (Y/N) |
|---|---|---|---|---|---|---|
| hsa-miR-185-5p | 002271 | -2.84 (-0.27; -5.35) | 0.0309 | 0.1659 | 1 | Y |
| hsa-miR-186-3p | 002105 | 1.21 (2.35; 0.08) | 0.0361 | 0.1659 | 1 | Y |
| hsa-miR-186-5p | 002285 | -2.57 (0.41; -5.45) | 0.0901 | 0.2585 | 1 | N |
| hsa-miR-18a-3p | 002423 | -0.54 (1.46; -2.50) | 0.5938 | 0.7344 | 0 | N |
| hsa-miR-18a-5p | 002422 | -3.99 (-1.17; -6.72) | 0.0058 | 0.0877 | 1 | Y |
| hsa-miR-18b-3p | 002310 | 0.75 (2.46; -0.94) | 0.3876 | 0.6052 | 0 | N |
| hsa-miR-18b-5p | 002217 | 1.61 (4.01; -0.74) | 0.1804 | 0.4029 | 0 | N |
| hsa-miR-190a-5p | 000489 | -2.78 (0.21; -5.68) | 0.0684 | 0.2179 | 1 | N |
| hsa-miR-190b | 002263 | 0.58 (3.03; -1.81) | 0.6363 | 0.7685 | 0 | N |
| hsa-miR-191-3p | 002678 | -0.56 (1.36; -2.44) | 0.5649 | 0.7193 | 0 | N |
| hsa-miR-191-5p | 002299 | -1.25 (1.11; -3.56) | 0.2957 | 0.5408 | 1 | N |
| hsa-miR-192-3p | 002272 | 0.28 (2.03; -1.44) | 0.7481 | 0.8325 | 0 | N |
| hsa-miR-192-5p | 000491 | -3.62 (0.05; -7.15) | 0.0531 | 0.1917 | 1 | N |
| hsa-miR-193a-3p | 002250 | 0.42 (2.06; -1.21) | 0.6173 | 0.7526 | 0 | N |
| hsa-miR-193a-5p | 002281 | -3.49 (-0.42; -6.47) | 0.0261 | 0.1582 | 0 | N |
| hsa-miR-193b-5p | 002366 | 1.21 (2.35; 0.08) | 0.0365 | 0.1659 | 0 | N |
| hsa-miR-193b-3p | 002367 | -2.06 (0.84; -4.87) | 0.1618 | 0.3758 | 1 | N |
| hsa-miR-194-5p | 000493 | -2.81 (1.06; -6.54) | 0.1520 | 0.3658 | 1 | N |
| hsa-miR-195-3p | 002107 | 1.16 (2.98; -0.62) | 0.2029 | 0.4238 | 0 | N |
| hsa-miR-195-5p | 000494 | -2.33 (0.59; -5.17) | 0.1166 | 0.3086 | 1 | N |
| hsa-miR-196a-3p | 002336 | 0.87 (3.04; -1.25) | 0.4216 | 0.6076 | 0 | N |
| hsa-miR-196b-5p | 002215 | -1.41 (1.44; -4.17) | 0.3286 | 0.5551 | 0 | N |
| hsa-miR-197-3p | 000497 | 14.67 (24.38; 5.71) | 0.0010 | 0.0417 | 0 | N |
| hsa-miR-198 | 002273 | 0.91 (2.72; -0.88) | 0.3219 | 0.5539 | 0 | N |

| miRNA name | QuantStudio Assay ID | Δ (%) (95% CI) | Raw *P* | FDR *P* | >50% | Chosen for validation (Y/N) |
|---|---|---|---|---|---|---|
| hsa-miR-199a-5p | 000498 | -1.32 (2.33; -4.84) | 0.4715 | 0.6456 | 1 | N |
| hsa-miR-199a-3p; hsa-miR-199b-3p | 002304 | -2.37 (0.94; -5.58) | 0.1579 | 0.3710 | 1 | N |
| hsa-miR-199b-5p | 000500 | 1.82 (4.97; -1.24) | 0.2457 | 0.4818 | 1 | N |
| hsa-miR-19a-3p | 000395 | -1.15 (1.56; -3.79) | 0.4023 | 0.6074 | 1 | N |
| hsa-miR-19b-3p | 000396 | -1.76 (0.30; -3.79) | 0.0940 | 0.2614 | 1 | N |
| hsa-miR-19b-1-5p | 002425 | 1.55 (3.00; 0.12) | 0.0333 | 0.1659 | 1 | Y |
| hsa-miR-200a-3p | 000502 | 0.92 (2.22; -0.37) | 0.1629 | 0.3762 | 0 | N |
| hsa-miR-200b-3p | 002251 | -0.24 (2.08; -2.51) | 0.8367 | 0.8889 | 0 | N |
| hsa-miR-200c-3p | 002300 | 3.93 (7.12; 0.83) | 0.0126 | 0.1124 | 0 | N |
| hsa-miR-202-5p | 002362 | 0.92 (2.69; -0.82) | 0.3016 | 0.5448 | 0 | N |
| hsa-miR-202-3p | 002363 | -3.20 (-0.56; -5.78) | 0.0180 | 0.1308 | 0 | N |
| hsa-miR-203a-3p | 000507 | -4.59 (-1.28; -7.79) | 0.0070 | 0.0908 | 0 | N |
| hsa-miR-204-5p | 000508 | 0.56 (4.61; -3.32) | 0.7793 | 0.8512 | 1 | N |
| hsa-miR-205-5p | 000509 | -0.48 (2.15; -3.05) | 0.7172 | 0.8178 | 0 | N |
| hsa-miR-206 | 000510 | -1.55 (1.57; -4.58) | 0.3255 | 0.5539 | 1 | N |
| hsa-miR-208a-3p | 000511 | 1.46 (2.74; 0.19) | 0.0244 | 0.1491 | 0 | N |
| hsa-miR-20a-3p | 002437 | -2.48 (0.55; -5.42) | 0.1079 | 0.2902 | 1 | N |
| hsa-miR-20a-5p | 000580 | -2.59 (-0.28; -4.85) | 0.0283 | 0.1612 | 1 | Y |
| hsa-miR-20b-5p | 001014 | -0.63 (2.90; -4.04) | 0.7220 | 0.8201 | 1 | N |
| hsa-miR-21-3p | 002438 | 1.29 (2.49; 0.11) | 0.0319 | 0.1659 | 0 | N |
| hsa-miR-21-5p | 000397 | 0.06 (4.83; -4.50) | 0.9804 | 0.9877 | 1 | N |
| hsa-miR-210-3p | 000512 | -0.13 (6.67; -6.50) | 0.9685 | 0.9793 | 0 | N |
| hsa-miR-211-5p | 000514 | 1.14 (4.70; -2.30) | 0.5205 | 0.6764 | 0 | N |
| hsa-miR-212-3p | 000515 | -6.08 (-2.27; -9.74) | 0.0020 | 0.0523 | 0 | N |

| miRNA name | Quant Studio<br><br>Assay ID | Δ (%) (95% CI) | Raw *P* | FDR *P* | >50% | Chosen for validation (Y/N) |
|---|---|---|---|---|---|---|
| hsa-miR-181a-3p | 000516 | 0.06 (2.37; -2.20) | 0.9603 | 0.9728 | 0 | N |
| hsa-miR-214-3p | 002306 | -5.99 (-1.54; -10.24) | 0.0089 | 0.1013 | 0 | N |
| hsa-miR-215-5p | 000518 | 6.66 (11.61; 1.93) | 0.0054 | 0.0859 | 0 | N |
| hsa-miR-216b-5p | 002326 | 0.73 (2.52; -1.02) | 0.4163 | 0.6074 | 0 | N |
| hsa-miR-218-5p | 000521 | -5.30 (-2.57; -7.95) | 0.0002 | 0.0162 | 1 | Y |
| hsa-miR-218-1-3p | 002094 | 1.17 (2.82; -0.46) | 0.1603 | 0.3748 | 0 | N |
| hsa-miR-219a-5p | 000522 | 0.55 (2.22; -1.10) | 0.5178 | 0.6764 | 0 | N |
| hsa-miR-219a-2-3p | 002390 | 1.17 (2.81; -0.45) | 0.1566 | 0.3695 | 0 | N |
| hsa-miR-22-5p | 002301 | -3.25 (-0.08; -6.32) | 0.0447 | 0.1768 | 1 | Y |
| hsa-miR-22-3p | 000398 | -1.87 (2.89; -6.41) | 0.4341 | 0.6097 | 0 | N |
| hsa-miR-220c | 002211 | 0.47 (2.61; -1.62) | 0.6593 | 0.7812 | 0 | N |
| hsa-miR-221-5p | 002096 | 0.61 (2.14; -0.89) | 0.4274 | 0.6076 | 0 | N |
| hsa-miR-221-3p | 000524 | -2.37 (0.83; -5.48) | 0.1450 | 0.3553 | 1 | N |
| hsa-miR-222-5p | 002097 | 1.00 (2.56; -0.54) | 0.2052 | 0.4252 | 0 | N |
| hsa-miR-222-3p | 002276 | -1.11 (2.38; -4.47) | 0.5285 | 0.6842 | 1 | N |
| hsa-miR-223-5p | 002098 | -1.58 (1.40; -4.47) | 0.2955 | 0.5408 | 1 | N |
| hsa-miR-223-3p | 002295 | -1.75 (0.03; -3.49) | 0.0535 | 0.1919 | 1 | N |
| hsa-miR-224-5p | 002099 | -3.64 (0.00; -7.15) | 0.0500 | 0.1872 | 1 | Y |
| hsa-miR-23a-5p | 002439 | 0.16 (1.97; -1.63) | 0.8650 | 0.9113 | 0 | N |
| hsa-miR-23a-3p | 000399 | 4.37 (9.59; -0.59) | 0.0853 | 0.2520 | 0 | N |
| hsa-miR-23b-3p | 000400 | 4.07 (6.56; 1.63) | 0.0010 | 0.0417 | 0 | N |
| hsa-miR-24-3p | 000402 | -2.15 (-0.12; -4.13) | 0.0376 | 0.1659 | 1 | Y |
| hsa-miR-24-2-5p | 002441 | 0.46 (3.04; -2.05) | 0.7233 | 0.8201 | 0 | N |
| hsa-miR-25-5p | 002442 | 1.40 (2.56; 0.24) | 0.0178 | 0.1308 | 0 | N |

| miRNA name | Quant Studio<br><br>Assay ID | Δ (%) (95% CI) | Raw *P* | FDR *P* | >50% | Chosen for validation (Y/N) |
|---|---|---|---|---|---|---|
| hsa-miR-25-3p | 000403 | -3.25 (-0.75; -5.69) | 0.0111 | 0.1081 | 1 | Y |
| hsa-miR-26a-5p | 000405 | -1.86 (0.71; -4.36) | 0.1544 | 0.3658 | 1 | N |
| hsa-miR-26a-1-3p | 002443 | -1.05 (1.50; -3.54) | 0.4143 | 0.6074 | 0 | N |
| hsa-miR-26a-2-3p | 002115 | -0.22 (2.13; -2.52) | 0.8516 | 0.8995 | 0 | N |
| hsa-miR-26b-3p | 002444 | -2.59 (0.12; -5.22) | 0.0606 | 0.2050 | 0 | N |
| hsa-miR-26b-5p | 000407 | -2.94 (-0.56; -5.25) | 0.0158 | 0.1265 | 1 | Y |
| hsa-miR-27a-5p | 002445 | -0.79 (2.07; -3.58) | 0.5830 | 0.7299 | 0 | N |
| hsa-miR-27a-3p | 000408 | -4.23 (-1.47; -6.91) | 0.0029 | 0.0659 | 1 | Y |
| hsa-miR-27b-5p | 002174 | -0.29 (1.92; -2.44) | 0.7966 | 0.8631 | 0 | N |
| hsa-miR-27b-3p | 000409 | -5.01 (-2.01; -7.91) | 0.0012 | 0.0417 | 1 | Y |
| hsa-miR-28-5p | 000411 | -3.92 (-0.78; -6.97) | 0.0148 | 0.1204 | 1 | Y |
| hsa-miR-28-3p | 002446 | -1.21 (2.88; -5.13) | 0.5570 | 0.7110 | 1 | N |
| hsa-miR-296-5p | 000527 | 4.82 (10.19; -0.29) | 0.0648 | 0.2118 | 1 | N |
| hsa-miR-299-5p | 000600 | 1.39 (2.83; -0.03) | 0.0548 | 0.1952 | 0 | N |
| hsa-miR-29a-5p | 002447 | -2.83 (0.21; -5.79) | 0.0678 | 0.2174 | 1 | N |
| hsa-miR-29a-3p | 002112 | -2.59 (0.93; -5.98) | 0.1479 | 0.3598 | 1 | N |
| hsa-miR-29b-3p | 000413 | -4.12 (-0.93; -7.20) | 0.0117 | 0.1093 | 0 | N |
| hsa-miR-29b-2-5p | 002166 | -0.12 (2.19; -2.37) | 0.9208 | 0.9451 | 0 | N |
| hsa-miR-29c-5p | 001818 | -2.12 (0.51; -4.68) | 0.1138 | 0.3025 | 0 | N |
| hsa-miR-29c-3p | 000587 | -2.73 (0.03; -5.41) | 0.0527 | 0.1915 | 1 | N |
| hsa-miR-301a-3p | 000528 | -2.76 (0.07; -5.51) | 0.0555 | 0.1955 | 1 | N |
| hsa-miR-301b-3p | 002392 | -4.94 (-1.98; -7.82) | 0.0012 | 0.0417 | 1 | Y |
| hsa-miR-302a-5p | 002381 | 0.92 (2.69; -0.83) | 0.3043 | 0.5448 | 0 | N |
| hsa-miR-302a-3p | 000529 | 0.41 (3.81; -2.88) | 0.8090 | 0.8662 | 0 | N |

| miRNA name | Quant Studio Assay ID | Δ (%) (95% CI) | Raw *P* | FDR *P* | >50% | Chosen for validation (Y/N) |
|---|---|---|---|---|---|---|
| hsa-miR-302b-3p | 000531 | 0.85 (2.11; -0.40) | 0.1847 | 0.4074 | 0 | N |
| hsa-miR-302c-5p | 000534 | -0.98 (3.33; -5.11) | 0.6497 | 0.7748 | 0 | N |
| hsa-miR-302c-3p | 000533 | 1.60 (2.82; 0.39) | 0.0094 | 0.1013 | 0 | N |
| hsa-miR-302d-3p | 000535 | 1.26 (2.41; 0.13) | 0.0293 | 0.1646 | 0 | N |
| hsa-miR-30a-3p | 000416 | -2.45 (1.63; -6.37) | 0.2354 | 0.4656 | 1 | N |
| hsa-miR-30a-5p | 000417 | -3.05 (0.87; -6.82) | 0.1259 | 0.3268 | 1 | N |
| hsa-miR-30b-3p | 002129 | 0.38 (2.43; -1.63) | 0.7108 | 0.8156 | 0 | N |
| hsa-miR-30b-5p | 000602 | -3.07 (1.35; -7.29) | 0.1702 | 0.3866 | 1 | N |
| hsa-miR-30c-5p | 000419 | -3.04 (2.48; -8.26) | 0.2747 | 0.5207 | 1 | N |
| hsa-miR-30c-1-3p | 002108 | 0.74 (2.47; -0.96) | 0.3934 | 0.6074 | 0 | N |
| hsa-miR-30d-3p | 002305 | -1.35 (1.24; -3.86) | 0.3038 | 0.5448 | 1 | N |
| hsa-miR-30d-5p | 000420 | -2.50 (0.43; -5.34) | 0.0938 | 0.2614 | 1 | N |
| hsa-miR-30e-3p | 000422 | -4.70 (-0.79; -8.46) | 0.0191 | 0.1308 | 0 | N |
| hsa-miR-31-3p | 002113 | -1.45 (1.40; -4.21) | 0.3156 | 0.5500 | 0 | N |
| hsa-miR-31-5p | 002279 | 1.64 (5.91; -2.45) | 0.4368 | 0.6120 | 1 | N |
| hsa-miR-32-3p | 002111 | 0.92 (2.69; -0.83) | 0.3044 | 0.5448 | 0 | N |
| hsa-miR-32-5p | 002109 | -2.14 (0.93; -5.11) | 0.1698 | 0.3866 | 1 | N |
| hsa-miR-320a | 002277 | -1.58 (0.96; -4.05) | 0.2198 | 0.4438 | 1 | N |
| hsa-miR-320b | 002844 | -2.22 (0.70; -5.05) | 0.1343 | 0.3453 | 1 | N |
| hsa-miR-323a-3p | 002227 | -1.41 (1.68; -4.42) | 0.3668 | 0.5933 | 1 | N |
| hsa-miR-324-3p | 002161 | -3.66 (-0.44; -6.78) | 0.0264 | 0.1582 | 0 | N |
| hsa-miR-324-5p | 000539 | -4.07 (-0.68; -7.34) | 0.0190 | 0.1308 | 0 | N |
| hsa-miR-325 | 000540 | 1.06 (2.25; -0.11) | 0.0758 | 0.2334 | 0 | N |
| hsa-miR-326 | 000542 | -0.43 (2.83; -3.59) | 0.7933 | 0.8612 | 0 | N |

| miRNA name | Quant Studio<br><br>Assay ID | Δ (%) (95% CI) | Raw *P* | FDR *P* | >50% | Chosen for validation (Y/N) |
|---|---|---|---|---|---|---|
| hsa-miR-328-3p | 000543 | -2.58 (0.16; -5.24) | 0.0644 | 0.2118 | 1 | N |
| hsa-miR-329-3p | 001101 | 0.27 (2.26; -1.67) | 0.7836 | 0.8524 | 0 | N |
| hsa-miR-330-3p | 000544 | -4.89 (-0.42; -9.15) | 0.0324 | 0.1659 | 0 | N |
| hsa-miR-331-3p | 000545 | -2.84 (-0.51; -5.12) | 0.0172 | 0.1308 | 0 | N |
| hsa-miR-331-5p | 002233 | 2.82 (4.79; 0.89) | 0.0041 | 0.0777 | 1 | Y |
| hsa-miR-335-3p | 002185 | -0.44 (2.65; -3.44) | 0.7760 | 0.8509 | 0 | N |
| hsa-miR-335-5p | 000546 | -4.37 (-0.77; -7.83) | 0.0177 | 0.1308 | 0 | N |
| hsa-miR-337-3p | 002157 | 0.00 (1.96; -1.92) | 0.9995 | 0.9995 | 0 | N |
| hsa-miR-337-5p | 002156 | -0.73 (1.89; -3.28) | 0.5807 | 0.7292 | 0 | N |
| hsa-miR-338-3p | 002252 | 0.38 (2.22; -1.43) | 0.6819 | 0.7931 | 0 | N |
| hsa-miR-338-5P* | 002658 | 1.93 (4.86; -0.92) | 0.1862 | 0.4092 | 0 | N |
| hsa-miR-339-3p | 002184 | -1.03 (2.15; -4.10) | 0.5208 | 0.6764 | 1 | N |
| hsa-miR-339-5p | 002257 | -5.58 (0.94; -11.67) | 0.0917 | 0.2589 | 1 | N |
| hsa-miR-33a-3p | 002136 | 0.01 (1.88; -1.83) | 0.9909 | 0.9946 | 0 | N |
| hsa-miR-340-3p | 002259 | -2.92 (0.18; -5.93) | 0.0649 | 0.2118 | 1 | N |
| hsa-miR-340-5p | 002258 | -3.55 (-0.18; -6.81) | 0.0394 | 0.1659 | 1 | Y |
| hsa-miR-342-3p | 002260 | -2.49 (4.58; -9.08) | 0.4799 | 0.6523 | 0 | N |
| hsa-miR-342-5p | 002147 | 1.31 (2.49; 0.15) | 0.0271 | 0.1590 | 0 | N |
| hsa-miR-345-5p | 002186 | -2.18 (0.83; -5.10) | 0.1543 | 0.3658 | 1 | N |
| hsa-miR-346 | 000553 | 0.91 (2.70; -0.85) | 0.3113 | 0.5459 | 0 | N |
| hsa-miR-34a-3p | 002316 | -0.18 (3.35; -3.59) | 0.9199 | 0.9451 | 1 | N |
| hsa-miR-34a-5p | 000426 | 1.39 (9.50; -6.13) | 0.7257 | 0.8201 | 0 | N |
| hsa-miR-34b-3p | 002102 | 0.94 (2.21; -0.32) | 0.1430 | 0.3528 | 0 | N |
| hsa-miR-34c-5p | 000428 | -0.55 (4.19; -5.08) | 0.8148 | 0.8691 | 0 | N |

| miRNA name | Quant Studio<br><br>Assay ID | Δ (%) (95% CI) | Raw *P* | FDR *P* | >50% | Chosen for validation (Y/N) |
|---|---|---|---|---|---|---|
| hsa-miR-361-5p | 000554 | -4.06 (-0.66; -7.35) | 0.0198 | 0.1310 | 0 | N |
| hsa-miR-361-3p | 002116 | 1.19 (2.52; -0.12) | 0.0758 | 0.2334 | 0 | N |
| hsa-miR-362-5p | 001273 | -0.08 (2.88; -2.96) | 0.9572 | 0.9722 | 1 | N |
| hsa-miR-362-3p | 002117 | -2.71 (0.08; -5.42) | 0.0569 | 0.1986 | 0 | N |
| hsa-miR-363-3p | 001271 | 1.44 (6.53; -3.41) | 0.5676 | 0.7198 | 1 | N |
| hsa-miR-365a-3p; hsa-miR-365b-3p | 001020 | -1.20 (2.28; -4.56) | 0.4937 | 0.6676 | 1 | N |
| hsa-miR-367-3p | 000555 | -1.56 (4.52; -7.30) | 0.6061 | 0.7423 | 0 | N |
| hsa-miR-369-3p | 000557 | -1.82 (1.57; -5.09) | 0.2897 | 0.5371 | 1 | N |
| hsa-miR-370-3p | 002275 | 7.76 (14.08; 1.80) | 0.0102 | 0.1045 | 0 | N |
| hsa-miR-372-3p | 000560 | 0.13 (2.64; -2.32) | 0.9185 | 0.9451 | 0 | N |
| hsa-miR-373-3p | 000561 | -1.66 (0.56; -3.83) | 0.1409 | 0.3491 | 0 | N |
| hsa-miR-374a-5p | 000563 | -3.40 (-0.76; -5.97) | 0.0119 | 0.1093 | 0 | N |
| hsa-miR-374b-5p | 001319 | -1.93 (2.93; -6.55) | 0.4298 | 0.6076 | 1 | N |
| hsa-miR-374a-3p | 002125 | 0.96 (2.15; -0.21) | 0.1087 | 0.2905 | 0 | N |
| hsa-miR-375 | 000564 | -6.30 (-2.92; -9.57) | 0.0003 | 0.0243 | 1 | Y |
| hsa-miR-376a-3p | 000565 | -11.14 (-5.91; -16.08) | 0.0001 | 0.0060 | 0 | N |
| hsa-miR-376b-3p | 001102 | 4.75 (7.73; 1.86) | 0.0012 | 0.0417 | 0 | N |
| hsa-miR-376c-3p | 002122 | 0.47 (4.41; -3.31) | 0.8088 | 0.8662 | 1 | N |
| hsa-miR-377-5p | 002128 | 0.91 (2.70; -0.84) | 0.3082 | 0.5453 | 0 | N |
| hsa-miR-377-3p | 000566 | 1.20 (2.35; 0.05) | 0.0401 | 0.1659 | 0 | N |
| hsa-miR-378a-5p | 000567 | 4.23 (8.61; 0.03) | 0.0481 | 0.1830 | 0 | N |
| hsa-miR-378* | 002243 | -0.86 (2.85; -4.44) | 0.6433 | 0.7723 | 0 | N |
| hsa-miR-379-5p | 001138 | 1.78 (5.42; -1.74) | 0.3252 | 0.5539 | 1 | N |
| hsa-miR-380-3p | 000569 | 1.20 (2.34; 0.07) | 0.0380 | 0.1659 | 0 | N |

| miRNA name | Quant Studio Assay ID | Δ (%) (95% CI) | Raw *P* | FDR *P* | >50% | Chosen for validation (Y/N) |
|---|---|---|---|---|---|---|
| hsa-miR-380-5p | 000570 | 0.88 (3.01; -1.21) | 0.4130 | 0.6074 | 0 | N |
| hsa-miR-381-3p | 000571 | 1.21 (6.71; -4.01) | 0.6557 | 0.7803 | 0 | N |
| hsa-miR-382-5p | 000572 | 5.26 (11.97; -1.05) | 0.1042 | 0.2840 | 0 | N |
| hsa-miR-383-5p | 000573 | 1.17 (2.34; 0.02) | 0.0463 | 0.1788 | 0 | N |
| hsa-miR-384 | 000574 | 0.45 (2.65; -1.69) | 0.6812 | 0.7931 | 0 | N |
| hsa-miR-409-3p | 002332 | -1.41 (3.18; -5.79) | 0.5413 | 0.6942 | 1 | N |
| hsa-miR-409-5p | 002331 | 0.64 (2.38; -1.07) | 0.4654 | 0.6408 | 0 | N |
| hsa-miR-410-3p | 001274 | -2.53 (0.81; -5.75) | 0.1357 | 0.3468 | 1 | N |
| hsa-miR-411-3p | 002238 | 1.05 (2.26; -0.14) | 0.0827 | 0.2472 | 0 | N |
| hsa-miR-411-5p | 001610 | -2.64 (1.38; -6.49) | 0.1950 | 0.4155 | 1 | N |
| hsa-miR-422a | 002297 | -0.93 (1.69; -3.49) | 0.4821 | 0.6537 | 0 | N |
| hsa-miR-423-5p | 002340 | -4.04 (-1.15; -6.84) | 0.0065 | 0.0895 | 1 | Y |
| hsa-miR-424-3p | 002309 | 2.38 (4.32; 0.48) | 0.0138 | 0.1187 | 0 | N |
| hsa-miR-424-5p | 000604 | -3.37 (-0.52; -6.13) | 0.0208 | 0.1336 | 0 | N |
| hsa-miR-425-3p | 002302 | -4.40 (-1.23; -7.48) | 0.0070 | 0.0908 | 0 | N |
| hsa-miR-425-5p | 001516 | 6.81 (12.86; 1.08) | 0.0192 | 0.1308 | 0 | N |
| hsa-miR-429 | 001024 | 0.58 (2.13; -0.95) | 0.4592 | 0.6368 | 0 | N |
| hsa-miR-431-5p | 001979 | 1.92 (3.88; 0.00) | 0.0502 | 0.1872 | 0 | N |
| hsa-miR-432-3p | 001027 | 0.52 (2.71; -1.62) | 0.6340 | 0.7685 | 0 | N |
| hsa-miR-432-5p | 001026 | 4.21 (7.32; 1.19) | 0.0060 | 0.0877 | 0 | N |
| hsa-miR-433-3p | 001028 | 2.19 (4.90; -0.45) | 0.1051 | 0.2851 | 0 | N |
| hsa-miR-449b-5p | 001608 | 0.94 (2.20; -0.31) | 0.1395 | 0.3472 | 0 | N |
| hsa-miR-450a-5p | 002303 | 1.12 (2.32; -0.07) | 0.0654 | 0.2121 | 0 | N |
| hsa-miR-450b-3p | 002208 | 0.79 (2.58; -0.97) | 0.3790 | 0.6026 | 0 | N |

| miRNA name | Quant Studio Assay ID | Δ (%) (95% CI) | Raw *P* | FDR *P* | >50% | Chosen for validation (Y/N) |
|---|---|---|---|---|---|---|
| hsa-miR-451a | 001141 | -1.71 (3.11; -6.30) | 0.4796 | 0.6523 | 1 | N |
| hsa-miR-452-3p | 002330 | 0.45 (2.65; -1.69) | 0.6816 | 0.7931 | 0 | N |
| hsa-miR-452-5p | 002329 | -0.49 (1.54; -2.48) | 0.6354 | 0.7685 | 0 | N |
| hsa-miR-454-5p | 001996 | -1.04 (1.37; -3.39) | 0.3945 | 0.6074 | 0 | N |
| hsa-miR-454-3p | 002323 | -0.25 (3.46; -3.82) | 0.8946 | 0.9340 | 1 | N |
| hsa-miR-455-5p | 001280 | 0.60 (2.12; -0.89) | 0.4312 | 0.6076 | 0 | N |
| hsa-miR-455-3p | 002244 | 0.81 (2.19; -0.56) | 0.2477 | 0.4838 | 0 | N |
| hsa-miR-483-3p | 002339 | 1.00 (2.62; -0.59) | 0.2167 | 0.4391 | 0 | N |
| hsa-miR-483-5p | 002338 | 0.23 (6.08; -5.30) | 0.9369 | 0.9579 | 1 | N |
| hsa-miR-484 | 001821 | -3.62 (1.02; -8.05) | 0.1240 | 0.3248 | 1 | N |
| hsa-miR-485-3p | 001277 | 1.81 (5.85; -2.08) | 0.3669 | 0.5933 | 1 | N |
| hsa-miR-486-5p | 001278 | 4.75 (8.04; 1.57) | 0.0033 | 0.0711 | 0 | N |
| hsa-miR-486-3p | 002093 | 1.95 (3.31; 0.62) | 0.0041 | 0.0777 | 0 | N |
| hsa-miR-487a-3p | 001279 | 1.16 (2.78; -0.43) | 0.1527 | 0.3658 | 0 | N |
| hsa-miR-487b-3p | 001285 | -1.49 (1.91; -4.78) | 0.3860 | 0.6052 | 1 | N |
| hsa-miR-488-5p | 001106 | 0.63 (2.15; -0.87) | 0.4103 | 0.6074 | 0 | N |
| hsa-miR-488-3p | 002357 | 0.73 (2.48; -0.99) | 0.4051 | 0.6074 | 0 | N |
| hsa-miR-489-3p | 002358 | 2.56 (4.20; 0.94) | 0.0019 | 0.0512 | 0 | N |
| hsa-miR-491-5p | 001630 | 1.67 (5.58; -2.10) | 0.3909 | 0.6052 | 1 | N |
| hsa-miR-491-3p | 002360 | 0.89 (2.63; -0.82) | 0.3075 | 0.5453 | 0 | N |
| hsa-miR-493-3p | 002364 | 0.73 (2.60; -1.11) | 0.4389 | 0.6133 | 0 | N |
| hsa-miR-494-3p | 002365 | 0.67 (3.90; -2.47) | 0.6802 | 0.7931 | 1 | N |
| hsa-miR-495-3p | 001663 | -1.21 (3.22; -5.46) | 0.5853 | 0.7299 | 1 | N |
| hsa-miR-496 | 001953 | 1.17 (2.32; 0.04) | 0.0416 | 0.1692 | 0 | N |

| miRNA name | Quant Studio<br><br>Assay ID | Δ (%) (95% CI) | Raw *P* | FDR *P* | >50% | Chosen for validation (Y/N) |
|---|---|---|---|---|---|---|
| hsa-miR-497-5p | 001043 | 0.64 (2.62; -1.30) | 0.5213 | 0.6764 | 0 | N |
| hsa-miR-499a-3p | 002427 | 0.72 (2.50; -1.03) | 0.4222 | 0.6076 | 0 | N |
| hsa-miR-500a-3p | 001046 | 0.64 (2.41; -1.09) | 0.4692 | 0.6441 | 0 | N |
| hsa-miR-500a-5p | 002428 | 1.52 (4.04; -0.94) | 0.2281 | 0.4586 | 0 | N |
| hsa-miR-501-3p | 002435 | 2.40 (4.11; 0.72) | 0.0050 | 0.0837 | 0 | N |
| hsa-miR-502-5p | 001109 | -4.86 (-1.93; -7.70) | 0.0013 | 0.0417 | 0 | N |
| hsa-miR-502-3p | 002083 | -1.45 (1.34; -4.16) | 0.3059 | 0.5448 | 0 | N |
| hsa-miR-503-5p | 001048 | -0.80 (1.64; -3.17) | 0.5177 | 0.6764 | 0 | N |
| hsa-miR-504-5p | 002084 | 0.92 (2.69; -0.83) | 0.3049 | 0.5448 | 0 | N |
| hsa-miR-505-5p | 002087 | -2.59 (0.48; -5.56) | 0.0974 | 0.2682 | 1 | N |
| hsa-miR-505-3p | 002089 | -1.75 (1.45; -4.85) | 0.2789 | 0.5223 | 0 | N |
| hsa-miR-508-3p | 001052 | 0.73 (2.95; -1.45) | 0.5143 | 0.6764 | 0 | N |
| hsa-miR-508-5p | 002092 | 1.16 (2.94; -0.58) | 0.1929 | 0.4155 | 0 | N |
| hsa-miR-509-5p | 002235 | 0.73 (2.57; -1.08) | 0.4304 | 0.6076 | 0 | N |
| hsa-miR-511-5p | 001111 | -1.29 (1.30; -3.81) | 0.3263 | 0.5539 | 0 | N |
| hsa-miR-512-3p | 001823 | 2.39 (5.66; -0.77) | 0.1395 | 0.3472 | 0 | N |
| hsa-miR-513a-5p | 002090 | 0.76 (2.45; -0.89) | 0.3691 | 0.5951 | 0 | N |
| hsa-miR-515-3p | 002369 | 0.62 (3.26; -1.95) | 0.6387 | 0.7685 | 0 | N |
| hsa-miR-516-3p* | 001149 | 0.62 (2.17; -0.91) | 0.4310 | 0.6076 | 0 | N |
| hsa-miR-516b-5p | 001150 | 1.42 (2.71; 0.15) | 0.0281 | 0.1612 | 0 | N |
| hsa-miR-517-5p | 001113 | 0.73 (2.49; -1.01) | 0.4141 | 0.6074 | 0 | N |
| hsa-miR-517a-3p;<br>hsa-miR-517b-3p | 002402 | 2.22 (4.71; -0.20) | 0.0725 | 0.2297 | 0 | N |
| hsa-miR-517c-3p | 001153 | 0.04 (2.17; -2.06) | 0.9740 | 0.9830 | 0 | N |
| hsa-miR-518a-3p | 002397 | 0.69 (2.90; -1.46) | 0.5300 | 0.6844 | 0 | N |

| miRNA name | Quant Studio Assay ID | Δ (%) (95% CI) | Raw *P* | FDR *P* | >50% | Chosen for validation (Y/N) |
|---|---|---|---|---|---|---|
| hsa-miR-518b | 001156 | 0.59 (3.14; -1.90) | 0.6466 | 0.7733 | 0 | N |
| hsa-miR-518d-3p | 001159 | 1.23 (3.15; -0.66) | 0.2023 | 0.4238 | 0 | N |
| hsa-miR-518f-5p | 002387 | 1.16 (2.93; -0.57) | 0.1891 | 0.4124 | 0 | N |
| hsa-miR-518f-3p | 002388 | -0.46 (3.13; -3.92) | 0.8004 | 0.8655 | 0 | N |
| hsa-miR-519a-3p | 002415 | 1.33 (2.47; 0.19) | 0.0219 | 0.1387 | 0 | N |
| hsa-miR-519b-3p | 002384 | -0.23 (1.66; -2.09) | 0.8062 | 0.8662 | 0 | N |
| hsa-miR-519d-3p | 002403 | 0.43 (2.04; -1.15) | 0.5942 | 0.7344 | 0 | N |
| hsa-miR-519e-3p | 002370 | 0.74 (2.20; -0.71) | 0.3179 | 0.5500 | 0 | N |
| hsa-miR-520b | 001116 | -4.06 (0.02; -7.98) | 0.0511 | 0.1894 | 0 | N |
| hsa-miR-520c-3p | 002400 | -4.08 (2.42; -10.17) | 0.2126 | 0.4339 | 0 | N |
| hsa-miR-520d-3p | 002743 | 1.20 (5.20; -2.64) | 0.5447 | 0.6968 | 0 | N |
| hsa-miR-520d-5p | 002393 | 1.23 (2.38; 0.09) | 0.0337 | 0.1659 | 0 | N |
| hsa-miR-520e | 001119 | 0.03 (7.46; -6.89) | 0.9938 | 0.9956 | 0 | N |
| hsa-miR-520f-3p | 001120 | 1.55 (3.64; -0.49) | 0.1367 | 0.3468 | 0 | N |
| hsa-miR-522-3p | 002413 | 1.50 (2.72; 0.30) | 0.0142 | 0.1187 | 0 | N |
| hsa-miR-523-3p | 002386 | 1.58 (6.30; -2.92) | 0.4973 | 0.6709 | 0 | N |
| hsa-miR-524-3p | 001173 | 1.16 (2.93; -0.57) | 0.1896 | 0.4124 | 0 | N |
| hsa-miR-524-5p | 001982 | 0.73 (2.49; -1.01) | 0.4142 | 0.6074 | 0 | N |
| hsa-miR-525-5p | 001174 | 2.07 (3.55; 0.62) | 0.0051 | 0.0837 | 0 | N |
| hsa-miR-526b-5p | 002382 | 1.25 (2.40; 0.12) | 0.0299 | 0.1659 | 0 | N |
| hsa-miR-532-5p | 001518 | 0.74 (4.46; -2.85) | 0.6917 | 0.7987 | 1 | N |
| hsa-miR-532-3p | 002355 | -1.95 (0.99; -4.80) | 0.1910 | 0.4132 | 1 | N |
| hsa-miR-539-5p | 001286 | 4.05 (7.69; 0.53) | 0.0238 | 0.1474 | 0 | N |
| hsa-miR-541-3p | 002201 | 1.21 (2.35; 0.08) | 0.0361 | 0.1659 | 0 | N |

| miRNA name | Quant Studio<br><br>Assay ID | Δ (%) (95% CI) | Raw *P* | FDR *P* | >50% | Chosen for validation (Y/N) |
|---|---|---|---|---|---|---|
| hsa-miR-542-3p | 001284 | -3.46 (-0.73; -6.10) | 0.0132 | 0.1162 | 0 | N |
| hsa-miR-542-5p | 002240 | 1.07 (2.59; -0.43) | 0.1621 | 0.3758 | 0 | N |
| hsa-miR-543 | 002376 | -0.32 (2.62; -3.17) | 0.8303 | 0.8838 | 1 | N |
| hsa-miR-544a | 002265 | 1.19 (2.34; 0.05) | 0.0402 | 0.1659 | 0 | N |
| hsa-miR-545-5p | 002266 | -0.99 (1.01; -2.96) | 0.3290 | 0.5551 | 0 | N |
| hsa-miR-545-3p | 002267 | -2.74 (-0.11; -5.30) | 0.0413 | 0.1692 | 0 | N |
| hsa-miR-548a-3p | 001538 | -1.98 (1.09; -4.97) | 0.2038 | 0.4239 | 0 | N |
| hsa-miR-548b-5p | 002408 | -1.75 (1.17; -4.58) | 0.2366 | 0.4656 | 0 | N |
| hsa-miR-548c-3p | 001590 | -3.14 (-0.22; -5.98) | 0.0353 | 0.1659 | 0 | N |
| hsa-miR-548am-5p;<br>hsa-miR-548c-5p;<br>hsa-miR-548o-5p | 002429 | -0.90 (1.03; -2.78) | 0.3587 | 0.5870 | 0 | N |
| hsa-miR-548d-3p | 001605 | -3.19 (-0.90; -5.43) | 0.0066 | 0.0895 | 0 | N |
| hsa-miR-548d-5p | 002237 | -0.78 (1.08; -2.61) | 0.4064 | 0.6074 | 0 | N |
| hsa-miR-548e-3p | 002881 | 0.92 (2.29; -0.43) | 0.1830 | 0.4066 | 0 | N |
| hsa-miR-548j-5p | 002783 | 0.41 (1.95; -1.12) | 0.6024 | 0.7395 | 0 | N |
| hsa-miR-548l | 002904 | 0.33 (2.52; -1.83) | 0.7685 | 0.8462 | 0 | N |
| hsa-miR-548p | 002798 | 0.18 (2.60; -2.19) | 0.8844 | 0.9270 | 0 | N |
| hsa-miR-550a-5p | 002410 | -0.36 (1.91; -2.58) | 0.7535 | 0.8363 | 0 | N |
| hsa-miR-551b-5p | 002346 | 1.58 (4.40; -1.16) | 0.2619 | 0.5044 | 0 | N |
| hsa-miR-551b-3p | 001535 | -4.66 (-0.54; -8.61) | 0.0270 | 0.1590 | 0 | N |
| hsa-miR-553 | 001521 | 0.36 (2.45; -1.70) | 0.7365 | 0.8242 | 0 | N |
| hsa-miR-555 | 001523 | 0.91 (2.70; -0.84) | 0.3096 | 0.5459 | 0 | N |
| hsa-miR-561-3p | 001528 | -0.60 (2.10; -3.22) | 0.6594 | 0.7812 | 0 | N |
| hsa-miR-564 | 001531 | 1.13 (2.29; -0.01) | 0.0520 | 0.1905 | 0 | N |

| miRNA name | Quant Studio Assay ID | Δ (%) (95% CI) | Raw *P* | FDR *P* | >50% | Chosen for validation (Y/N) |
|---|---|---|---|---|---|---|
| hsa-miR-567 | 001534 | 0.81 (2.56; -0.92) | 0.3619 | 0.5906 | 0 | N |
| hsa-miR-570-3p | 002347 | 0.09 (1.90; -1.69) | 0.9226 | 0.9451 | 0 | N |
| hsa-miR-571 | 001613 | 0.82 (2.54; -0.87) | 0.3430 | 0.5682 | 0 | N |
| hsa-miR-572 | 001614 | -2.90 (1.54; -7.14) | 0.1967 | 0.4171 | 1 | N |
| hsa-miR-574-3p | 002349 | -2.01 (1.19; -5.11) | 0.2158 | 0.4389 | 1 | N |
| hsa-miR-576-3p | 002351 | -3.52 (-0.59; -6.36) | 0.0190 | 0.1308 | 0 | N |
| hsa-miR-576-5p | 002350 | 1.19 (2.35; 0.05) | 0.0400 | 0.1659 | 0 | N |
| hsa-miR-577 | 002675 | -0.13 (1.82; -2.04) | 0.8944 | 0.9340 | 0 | N |
| hsa-miR-578 | 001619 | 0.74 (2.47; -0.95) | 0.3902 | 0.6052 | 0 | N |
| hsa-miR-579-3p | 002398 | -3.82 (-1.02; -6.54) | 0.0079 | 0.0949 | 0 | N |
| hsa-miR-580-3p | 001621 | 0.91 (2.09; -0.25) | 0.1258 | 0.3268 | 0 | N |
| hsa-miR-581 | 001622 | 0.92 (2.69; -0.83) | 0.3048 | 0.5448 | 0 | N |
| hsa-miR-584-5p | 001624 | 1.12 (2.39; -0.12) | 0.0770 | 0.2344 | 0 | N |
| hsa-miR-585-3p | 001625 | 0.33 (2.53; -1.82) | 0.7624 | 0.8428 | 0 | N |
| hsa-miR-586 | 001539 | 0.31 (2.54; -1.87) | 0.7811 | 0.8514 | 0 | N |
| hsa-miR-589-3p | 001543 | 2.23 (3.75; 0.72) | 0.0037 | 0.0750 | 0 | N |
| hsa-miR-589-5p | 002409 | 1.18 (2.33; 0.06) | 0.0397 | 0.1659 | 0 | N |
| hsa-miR-590-3p | 002677 | -3.22 (0.11; -6.44) | 0.0577 | 0.1989 | 1 | Y |
| hsa-miR-590-5p | 001984 | -3.29 (-0.58; -5.93) | 0.0177 | 0.1308 | 0 | N |
| hsa-miR-591 | 001545 | 1.65 (2.89; 0.43) | 0.0080 | 0.0949 | 0 | N |
| hsa-miR-592 | 001546 | 0.60 (2.38; -1.14) | 0.4990 | 0.6715 | 0 | N |
| hsa-miR-597-5p | 001551 | -1.54 (1.47; -4.45) | 0.3115 | 0.5459 | 1 | N |
| hsa-miR-598-3p | 001988 | -3.77 (-0.68; -6.76) | 0.0172 | 0.1308 | 1 | Y |
| hsa-miR-601 | 001558 | -6.89 (-2.08; -11.47) | 0.0055 | 0.0859 | 0 | N |

| miRNA name | Quant Studio<br><br>Assay ID | Δ (%) (95% CI) | Raw *P* | FDR *P* | >50% | Chosen for validation (Y/N) |
|---|---|---|---|---|---|---|
| hsa-miR-603 | 001566 | -2.35 (0.39; -5.01) | 0.0916 | 0.2589 | 0 | N |
| hsa-miR-604 | 001567 | 0.58 (2.12; -0.94) | 0.4554 | 0.6332 | 0 | N |
| hsa-miR-605-5p | 001568 | 1.22 (2.37; 0.08) | 0.0359 | 0.1659 | 0 | N |
| hsa-miR-613 | 001586 | 0.73 (2.49; -1.01) | 0.4141 | 0.6074 | 0 | N |
| hsa-miR-616-5p | 001589 | 0.22 (2.04; -1.57) | 0.8129 | 0.8687 | 0 | N |
| hsa-miR-616-3p | 002414 | 1.22 (2.46; -0.01) | 0.0521 | 0.1905 | 0 | N |
| hsa-miR-617 | 001591 | 0.72 (2.49; -1.01) | 0.4160 | 0.6074 | 0 | N |
| hsa-miR-618 | 001593 | -3.77 (2.49; -9.65) | 0.2315 | 0.4622 | 0 | N |
| hsa-miR-622 | 001553 | 0.30 (2.06; -1.43) | 0.7358 | 0.8242 | 0 | N |
| hsa-miR-623 | 001555 | -1.45 (2.89; -5.61) | 0.5054 | 0.6741 | 0 | N |
| hsa-miR-624-5p | 001557 | 1.84 (4.69; -0.93) | 0.1943 | 0.4155 | 0 | N |
| hsa-miR-625-3p | 002432 | -8.77 (-4.80; -12.57) | 0.0000 | 0.0035 | 1 | Y |
| hsa-miR-625-5p | 002431 | -2.87 (0.15; -5.79) | 0.0620 | 0.2060 | 0 | N |
| hsa-miR-626 | 001559 | 0.73 (2.49; -1.01) | 0.4141 | 0.6074 | 0 | N |
| hsa-miR-627-5p | 001560 | -1.69 (1.24; -4.54) | 0.2537 | 0.4920 | 0 | N |
| hsa-miR-628-3p | 002434 | -1.08 (1.48; -3.57) | 0.4048 | 0.6074 | 0 | N |
| hsa-miR-628-5p | 002433 | 1.17 (11.09; -7.86) | 0.8068 | 0.8662 | 1 | N |
| hsa-miR-629-3p | 001562 | -0.24 (2.86; -3.25) | 0.8772 | 0.9212 | 1 | N |
| hsa-miR-629-5p | 002436 | -0.79 (1.96; -3.47) | 0.5684 | 0.7198 | 0 | N |
| hsa-miR-630 | 001563 | 0.64 (2.89; -1.56) | 0.5703 | 0.7198 | 0 | N |
| hsa-miR-635 | 001578 | 0.72 (2.87; -1.39) | 0.5089 | 0.6748 | 0 | N |
| hsa-miR-636 | 002088 | 7.18 (16.06; -1.01) | 0.0873 | 0.2530 | 0 | N |
| hsa-miR-638 | 001582 | -1.84 (2.79; -6.27) | 0.4287 | 0.6076 | 1 | N |
| hsa-miR-640 | 001584 | 1.17 (2.32; 0.04) | 0.0429 | 0.1720 | 0 | N |

| miRNA name | Quant Studio Assay ID | Δ (%) (95% CI) | Raw *P* | FDR *P* | >50% | Chosen for validation (Y/N) |
|---|---|---|---|---|---|---|
| hsa-miR-641 | 001585 | 1.03 (2.17; -0.10) | 0.0735 | 0.2306 | 0 | N |
| hsa-miR-642a-5p | 001592 | -2.90 (0.43; -6.12) | 0.0473 | 0.1530 | 1 | Y |
| hsa-miR-643 | 001594 | 1.05 (2.22; -0.10) | 0.0740 | 0.2306 | 0 | N |
| hsa-miR-645 | 001597 | -1.54 (1.68; -4.66) | 0.3446 | 0.5692 | 1 | N |
| hsa-miR-646 | 001599 | 0.54 (3.77; -2.58) | 0.7358 | 0.8242 | 0 | N |
| hsa-miR-648 | 001601 | 1.26 (2.59; -0.05) | 0.0596 | 0.2031 | 0 | N |
| hsa-miR-649 | 001602 | 0.36 (2.44; -1.68) | 0.7290 | 0.8209 | 0 | N |
| hsa-miR-650 | 001603 | 1.21 (2.37; 0.06) | 0.0386 | 0.1659 | 0 | N |
| hsa-miR-651-5p | 001604 | 1.16 (3.29; -0.93) | 0.2786 | 0.5223 | 0 | N |
| hsa-miR-652-3p | 002352 | -6.42 (-2.51; -10.17) | 0.0015 | 0.0434 | 1 | Y |
| hsa-miR-654-5p | 001611 | 0.44 (2.84; -1.91) | 0.7172 | 0.8178 | 0 | N |
| hsa-miR-654-3p | 002239 | -0.62 (1.29; -2.50) | 0.5204 | 0.6764 | 0 | N |
| hsa-miR-655-3p | 001612 | 0.62 (2.87; -1.58) | 0.5852 | 0.7299 | 0 | N |
| hsa-miR-656-3p | 001510 | 0.68 (2.35; -0.96) | 0.4186 | 0.6076 | 0 | N |
| hsa-miR-659-3p | 001514 | 0.77 (3.15; -1.56) | 0.5195 | 0.6764 | 0 | N |
| hsa-miR-660-5p | 001515 | -2.10 (0.33; -4.47) | 0.0890 | 0.2567 | 1 | N |
| hsa-miR-661 | 001606 | 1.12 (2.45; -0.19) | 0.0943 | 0.2614 | 0 | N |
| hsa-miR-662 | 001607 | 1.26 (2.43; 0.10) | 0.0326 | 0.1659 | 0 | N |
| hsa-miR-663b | 002857 | 0.77 (2.50; -0.94) | 0.3792 | 0.6026 | 0 | N |
| hsa-miR-664a-3p | 002897 | 8.01 (12.66; 3.55) | 0.0004 | 0.0243 | 0 | N |
| hsa-miR-665 | 002681 | 1.17 (2.32; 0.03) | 0.0448 | 0.1768 | 0 | N |
| hsa-miR-668-3p | 001992 | 1.51 (3.25; -0.21) | 0.0856 | 0.2520 | 0 | N |
| hsa-miR-671-3p | 002322 | -1.37 (1.39; -4.05) | 0.3262 | 0.5539 | 1 | N |
| hsa-miR-674 | 002021 | 2.39 (4.24; 0.56) | 0.0101 | 0.1045 | 0 | N |

| miRNA name | Quant Studio Assay ID | Δ (%) (95% CI) | Raw *P* | FDR *P* | >50% | Chosen for validation (Y/N) |
|---|---|---|---|---|---|---|
| hsa-miR-7-1-3p | 001338 | -1.95 (2.17; -5.91) | 0.3480 | 0.5730 | 1 | N |
| hsa-miR-7-5p | 000268 | -2.36 (0.41; -5.06) | 0.0945 | 0.2614 | 0 | N |
| hsa-miR-7-2-3p | 002314 | 1.19 (2.34; 0.06) | 0.0390 | 0.1659 | 0 | N |
| hsa-miR-708-5p | 002341 | 1.02 (4.37; -2.22) | 0.5409 | 0.6942 | 0 | N |
| hsa-miR-720* | 002895 | -2.35 (-0.11; -4.54) | 0.0397 | 0.1659 | 0 | N |
| hsa-miR-744-3p | 002325 | -3.45 (-0.86; -5.97) | 0.0095 | 0.1013 | 1 | Y |
| hsa-miR-744-5p | 002324 | -3.48 (-0.50; -6.38) | 0.0227 | 0.1423 | 0 | N |
| hsa-miR-758-3p | 001990 | -0.96 (3.81; -5.52) | 0.6866 | 0.7962 | 0 | N |
| hsa-miR-766-3p | 001986 | -2.78 (-0.04; -5.45) | 0.0470 | 0.1805 | 1 | Y |
| hsa-miR-767-3p | 001995 | 0.93 (2.28; -0.40) | 0.1719 | 0.3886 | 0 | N |
| hsa-miR-769-5p | 001998 | -0.64 (2.12; -3.32) | 0.6470 | 0.7733 | 1 | N |
| hsa-miR-770-5p | 002002 | 0.33 (2.53; -1.82) | 0.7657 | 0.8448 | 0 | N |
| hsa-miR-802 | 002004 | 0.42 (2.47; -1.58) | 0.6825 | 0.7931 | 0 | N |
| mmu-miR-871-5p | 002354 | 0.88 (3.06; -1.26) | 0.4218 | 0.6076 | 0 | N |
| hsa-miR-874-3p | 002268 | 0.86 (2.59; -0.84) | 0.3235 | 0.5539 | 0 | N |
| hsa-miR-875-5p | 002203 | 1.24 (3.09; -0.58) | 0.1835 | 0.4066 | 0 | N |
| hsa-miR-876-5p | 002205 | 0.74 (2.47; -0.95) | 0.3902 | 0.6052 | 0 | N |
| hsa-miR-885-5p | 002296 | 0.83 (5.37; -3.51) | 0.7133 | 0.8167 | 1 | N |
| hsa-miR-886-3p | 002194 | -2.32 (0.97; -5.50) | 0.1648 | 0.3789 | 1 | N |
| hsa-miR-886-5p | 002193 | -2.55 (0.57; -5.57) | 0.1081 | 0.2902 | 1 | N |
| hsa-miR-888-5p | 002212 | 1.03 (5.83; -3.56) | 0.6667 | 0.7865 | 0 | N |
| hsa-miR-889-3p | 002202 | -1.42 (1.82; -4.56) | 0.3860 | 0.6052 | 1 | N |
| hsa-miR-891b | 002210 | 0.90 (2.33; -0.50) | 0.2068 | 0.4269 | 0 | N |
| hsa-miR-892b | 002214 | 1.23 (2.37; 0.10) | 0.0335 | 0.1659 | 0 | N |

| miRNA name | Quant Studio<br><br>Assay ID | Δ (%) (95% CI) | Raw *P* | FDR *P* | >50% | Chosen for validation (Y/N) |
|---|---|---|---|---|---|---|
| hsa-miR-9-3p | 002231 | -4.52 (-1.51; -7.44) | 0.0035 | 0.0740 | 1 | Y |
| hsa-miR-9-5p | 000583 | -0.84 (3.10; -4.63) | 0.6701 | 0.7888 | 0 | N |
| hsa-miR-92a-3p | 000431 | -3.00 (-0.75; -5.19) | 0.0091 | 0.1013 | 1 | Y |
| hsa-miR-92a-1-5p | 002137 | -0.07 (2.10; -2.19) | 0.9491 | 0.9687 | 0 | N |
| hsa-miR-92a-2-5p | 002138 | 1.16 (2.89; -0.53) | 0.1800 | 0.4029 | 0 | N |
| hsa-miR-93-3p | 002139 | -2.13 (0.81; -4.98) | 0.1538 | 0.3658 | 1 | N |
| hsa-miR-93-5p | 001090 | -3.06 (-0.49; -5.57) | 0.0202 | 0.1310 | 0 | N |
| hsa-miR-939-5p | 002182 | 0.56 (2.09; -0.94) | 0.4656 | 0.6408 | 0 | N |
| hsa-miR-941 | 002183 | 0.72 (2.49; -1.02) | 0.4168 | 0.6074 | 0 | N |
| hsa-miR-942-5p | 002187 | -9.86 (-5.57; -13.96) | 0.0000 | 0.0024 | 0 | N |
| hsa-miR-943 | 002188 | 1.24 (2.39; 0.10) | 0.0333 | 0.1659 | 0 | N |
| hsa-miR-944 | 002189 | 1.04 (2.24; -0.15) | 0.0868 | 0.2530 | 0 | N |
| hsa-miR-95-3p | 000433 | -2.71 (0.96; -6.25) | 0.1454 | 0.3553 | 1 | N |
| hsa-miR-96-5p | 000186 | -1.20 (2.12; -4.41) | 0.4737 | 0.6470 | 0 | N |
| hsa-miR-98-5p | 000577 | -1.46 (4.78; -7.32) | 0.6388 | 0.7685 | 0 | N |
| hsa-miR-99a-3p | 002141 | -0.01 (1.86; -1.86) | 0.9893 | 0.9946 | 0 | N |
| hsa-miR-99a-5p | 000435 | 1.76 (5.98; -2.29) | 0.3986 | 0.6074 | 1 | N |
| hsa-miR-99b-3p | 002196 | 1.57 (5.42; -2.15) | 0.4132 | 0.6074 | 0 | N |
| hsa-miR-99b-5p | 000436 | -3.50 (-0.94; -5.99) | 0.0076 | 0.0949 | 1 | Y |

*miRNAs no longer present in the last version of miRBase (v.21). Value “1” reported in column “>50%” indicates miRNAs expressed in at least 50% of subjects. Raw *P* < 0.05 and FDR *P* < 0.10 are highlighted in red.
