# Supplementary material for: Extracellular vesicle-packaged miRNA release after short-term exposure to particulate matter is associated with increased coagulation
Source: Part Fibre Toxicol. 2017 Aug 24;14:32. doi: 10.1186/s12989-017-0214-4 (PMC5594543; doi:10.1186/s12989-017-0214-4)
Supplement: Supplementary file 8 — Validation of the top-40 differentially expressed miRNAs. Association between Day −1–PM10 exposure levels and EV-miRNAs measured by OpenArray. (PDF 554 kb) [file 12989_2017_214_MOESM8_ESM.pdf]

Additional file 8: Supplementary Table S3: Validation of the top-40 differentially expressed miRNAs. Association between Day -1–PM_{10} exposure levels and EV-miRNAs measured by OpenArray.

**miRNA name**	**Assay ID**	**Δ(%) (95% CI)**	**Raw *P***
hsa-let-7c-5p	379	-2.72 (-0.31; -5.08)	0.0270
hsa-let-7d-5p	2283	-1.40 (1.33; -4.05)	0.3101
hsa-let-7g-5p	2282	-1.60 (1.00; -4.14)	0.2243
hsa-miR-106a-5p	2169	-2.59 (-0.11; -5.01)	0.0409
hsa-miR-125b-5p	449	-1.88 (0.61; -4.30)	0.1368
hsa-miR-143-3p	2249	-2.75 (-0.04; -5.39)	0.0467
hsa-miR-148a-3p	470	-0.90 (1.82; -3.55)	0.5109
hsa-miR-148b-3p	471	-1.61 (1.29; -4.42)	0.2729
hsa-miR-152-3p	475	-1.20 (1.48; -3.81)	0.3770
hsa-miR-181a-2-3p	2317	4.15 (12.82; -3.86)	0.3193
hsa-miR-184	485	-0.83 (3.43; -4.91)	0.6973
hsa-miR-185-5p	2271	-2.78 (-0.19; -5.31)	0.0359
hsa-miR-186-3p	2105	-2.29 (0.08; -4.61)	0.0583
hsa-miR-18a-5p	2422	-0.78 (1.98; -3.47)	0.5756
hsa-miR-19b-1-5p	2425	0.25 (3.32; -2.74)	0.8735
hsa-miR-20a-5p	580	-0.76 (2.11; -3.55)	0.5987
hsa-miR-218-5p	521	-4.20 (-1.87; -6.47)	0.0005
hsa-miR-224-5p	2099	-0.58 (2.53; -3.60)	0.7091
hsa-miR-22-5p	2301	0.74 (3.70; -2.13)	0.6161
hsa-miR-24-3p	402	-1.96 (1.04; -4.87)	0.1980
hsa-miR-25-3p	403	-1.36 (1.23; -3.88)	0.2995
hsa-miR-26b-5p	407	-1.26 (1.22; -3.67)	0.3175
hsa-miR-27a-3p	408	-1.93 (0.96; -4.74)	0.1875
hsa-miR-27b-3p	409	-0.55 (2.39; -3.41)	0.7095
hsa-miR-28-5p	411	-2.07 (0.94; -4.98)	0.1753
hsa-miR-301b-3p	2392	-0.61 (2.74; -3.86)	0.7158
hsa-miR-331-5p	2233	-3.07 (-0.26; -5.80)	0.0328
hsa-miR-340-5p	2258	-1.80 (1.38; -4.88)	0.2638
hsa-miR-375	564	-1.52 (2.00; -4.91)	0.3925
hsa-miR-423-5p	2340	-0.89 (1.54; -3.25)	0.4703
hsa-miR-590-3p	2677	-2.07 (0.79; -4.84)	0.1544
hsa-miR-598-3p	1988	-1.88 (0.99; -4.68)	0.1966
hsa-miR-625-3p	2432	1.18 (4.69; -2.21)	0.4987
hsa-miR-642a-5p	1592	-3.51 (-0.17; -6.74)	0.0397
hsa-miR-652-3p	2352	-2.69 (-0.03; -5.29)	0.0478
hsa-miR-744-3p	2325	-2.21 (1.29; -5.59)	0.2133
hsa-miR-766-3p	1986	-1.41 (1.65; -4.38)	0.3619
hsa-miR-92a-3p	436	-1.20 (1.10; -3.44)	0.3030
hsa-miR-99b-5p	2231	-3.14 (-0.56; -5.65)	0.0173
hsa-miR-9-3p	431	-2.10 (0.85; -4.97)	0.1608

Raw $P < 0.05$ highlighted in red.
