# Supplementary material for: Extracellular vesicle-packaged miRNA release after short-term exposure to particulate matter is associated with increased coagulation
Source: Part Fibre Toxicol. 2017 Aug 24;14:32. doi: 10.1186/s12989-017-0214-4 (PMC5594543; doi:10.1186/s12989-017-0214-4)
Supplement: Supplementary file 9 — List of the genes involved in CVDs according to DisGeNET database. For each gene all the related diseases and the putative EV-MiRNAs targeting it are indicated both as list and as number of occurrences. (PDF 1206 kb) [file 12989_2017_214_MOESM9_ESM.pdf]

Additional file 9: Supplementary Table S4: List of the genes involved in CVDs according to DisGeNET database. For each gene all the related diseases and the putative EV-MiRNAs targeting it are indicated both as list and as number of occurrences.

**Entrez ID**	**Gene**	**CVDs (DisGeNET db v4.0)**	**CVD number**	**EV-miRNA targets**	**EV-miRNA number**
7078	*TIMP3*	Asthma	1	hsa-let-7c-5p; hsa-miR-331-3p; hsa-miR-143-3p; hsa-miR-652-3p; hsa-miR-218-5p; hsa-miR-185-5p; hsa-miR-106a-5p; hsa-miR-642a-5p; hsa-miR-99b-5p	9
1075	*CTSC*	Myocardial Ischemia (MI)	1	hsa-miR-331-3p; hsa-miR-143-3p; hsa-let-7c-5p; hsa-miR-642a-5p; hsa-miR-106a-5p; hsa-miR-185-5p; hsa-miR-652-3p; hsa-miR-218-5p	8
5733	*PTGER3*	Thrombosis	1	hsa-miR-642a-5p; hsa-miR-106a-5p; hsa-miR-185-5p; hsa-miR-652-3p; hsa-miR-218-5p; hsa-miR-331-3p; hsa-miR-143-3p; hsa-let-7c-5p	8
6376	*CX3CL1*	Myocardial Ischemia (MI)	1	hsa-let-7c-5p; hsa-miR-331-3p; hsa-miR-143-3p; hsa-miR-642a-5p; hsa-miR-652-3p; hsa-miR-218-5p; hsa-miR-106a-5p; hsa-miR-185-5p	8
894	*CCND2*	Myocardial Ischemia (MI)	1	hsa-miR-642a-5p; hsa-miR-185-5p; hsa-miR-106a-5p; hsa-miR-218-5p; hsa-miR-652-3p; hsa-miR-143-3p; hsa-miR-331-3p; hsa-let-7c-5p	8
79924	*ADM2*	Myocardial Ischemia (MI)	1	hsa-let-7c-5p; hsa-miR-331-3p; hsa-miR-143-3p; hsa-miR-642a-5p; hsa-miR-218-5p; hsa-miR-652-3p; hsa-miR-185-5p; hsa-miR-106a-5p	8
10019	*SH2B3*	Myocardial Infarction; Inflammation	2	hsa-miR-331-3p; hsa-miR-143-3p; hsa-let-7c-5p; hsa-miR-642a-5p; hsa-miR-106a-5p; hsa-miR-185-5p; hsa-miR-218-5p; hsa-miR-652-3p	8
4660	*PPP1R12B*	Coronary heart disease	1	hsa-miR-143-3p; hsa-miR-331-3p; hsa-let-7c-5p; hsa-miR-642a-5p; hsa-miR-106a-5p; hsa-miR-185-5p; hsa-miR-218-5p; hsa-miR-652-3p	8
6533	*SLC6A6*	Myocardial Ischemia (MI)	1	hsa-let-7c-5p; hsa-miR-143-3p; hsa-miR-331-3p; hsa-miR-642a-5p; hsa-miR-652-3p; hsa-miR-218-5p; hsa-miR-185-5p; hsa-miR-106a-5p	8

Entrez ID	Gene	CVDs (DisGeNET db v4.0)	CVD number	EV-miRNA targets	EV-miRNA number
4646	*MYO6*	Hypertensive disease	1	hsa-let-7c-5p; hsa-miR-642a-5p; hsa-miR-143-3p; hsa-miR-652-3p; hsa-miR-218-5p; hsa-miR-185-5p; hsa-miR-106a-5p	7
54897	*CASZ1*	Hypertensive disease	1	hsa-miR-185-5p; hsa-miR-652-3p; hsa-miR-218-5p; hsa-miR-99b-5p; hsa-miR-331-3p; hsa-miR-642a-5p; hsa-let-7c-5p	7
23194	*FBXL7*	Asthma	1	hsa-let-7c-5p; hsa-miR-642a-5p; hsa-miR-143-3p; hsa-miR-218-5p; hsa-miR-652-3p; hsa-miR-185-5p; hsa-miR-106a-5p	7
6095	*RORA*	Asthma	1	hsa-miR-106a-5p; hsa-miR-185-5p; hsa-miR-218-5p; hsa-miR-652-3p; hsa-miR-331-3p; hsa-miR-143-3p; hsa-miR-642a-5p	7
108	*ADCY2*	Asthma	1	hsa-miR-185-5p; hsa-miR-106a-5p; hsa-miR-652-3p; hsa-miR-218-5p; hsa-miR-331-3p; hsa-miR-143-3p; hsa-let-7c-5p	7
128611	*ZNF831*	Hypertensive disease	1	hsa-miR-331-3p; hsa-miR-143-3p; hsa-miR-642a-5p; hsa-miR-106a-5p; hsa-miR-185-5p; hsa-miR-218-5p; hsa-miR-652-3p	7
6558	*SLC12A2*	Cardiovascular Diseases; Hypertensive disease	2	hsa-miR-106a-5p; hsa-miR-185-5p; hsa-miR-218-5p; hsa-miR-99b-5p; hsa-miR-143-3p; hsa-miR-642a-5p; hsa-let-7c-5p	7
596	*BCL2*	Myocardial Infarction; Asthma; Hypertensive disease; Cerebral Hemorrhage	4	hsa-let-7c-5p; hsa-miR-642a-5p; hsa-miR-143-3p; hsa-miR-652-3p; hsa-miR-218-5p; hsa-miR-185-5p; hsa-miR-106a-5p	7
7157	*TP53*	Ischemia; Hypertensive disease	2	hsa-miR-143-3p; hsa-miR-331-3p; hsa-miR-642a-5p; hsa-let-7c-5p; hsa-miR-185-5p; hsa-miR-652-3p; hsa-miR-218-5p	7
8927	*BSN*	Heart Diseases	1	hsa-miR-185-5p; hsa-miR-652-3p; hsa-miR-218-5p; hsa-miR-331-3p; hsa-miR-143-3p; hsa-miR-642a-5p; hsa-let-7c-5p	7
1947	*EFNB1*	Inflammation	1	hsa-let-7c-5p; hsa-miR-642a-5p; hsa-miR-143-3p; hsa-miR-218-5p; hsa-miR-652-3p; hsa-miR-185-5p; hsa-miR-106a-5p	7
1952	*CELSR2*	Coronary heart disease; Cardiovascular Diseases; Myocardial Infarction	3	hsa-miR-218-5p; hsa-miR-106a-5p; hsa-miR-185-5p; hsa-miR-642a-5p; hsa-let-7c-5p; hsa-miR-331-3p; hsa-miR-143-3p	7
57608	*KIAA1462*	Coronary heart disease	1	hsa-miR-642a-5p; hsa-let-7c-5p; hsa-miR-143-3p; hsa-miR-652-3p; hsa-miR-218-5p; hsa-miR-106a-5p; hsa-miR-185-5p	7
477	*ATP1A2*	Hypertensive disease	1	hsa-miR-185-5p; hsa-miR-106a-5p; hsa-miR-652-3p; hsa-miR-218-5p; hsa-miR-143-3p; hsa-miR-99b-5p; hsa-miR-642a-5p	7

Entrez ID	Gene	CVDs (DisGeNET db v4.0)	CVD number	EV-miRNA targets	EV-miRNA number
23274	*CLEC16A*	Asthma	1	hsa-miR-642a-5p; hsa-miR-99b-5p; hsa-miR-143-3p; hsa-miR-331-3p; hsa-miR-218-5p; hsa-miR-106a-5p; hsa-miR-185-5p	7
2029	*ENSA*	Myocardial Ischemia (MI)	1	hsa-miR-106a-5p; hsa-miR-185-5p; hsa-miR-143-3p; hsa-miR-331-3p; hsa-miR-642a-5p; hsa-let-7c-5p	6
5743	*PTGS2*	Hypertensive disease; Thrombosis; Heart failure; Cardiovascular Diseases; Cardiac Arrhythmia; Inflammation	6	hsa-let-7c-5p; hsa-miR-642a-5p; hsa-miR-143-3p; hsa-miR-652-3p; hsa-miR-185-5p; hsa-miR-106a-5p	6
3659	*IRF1*	Myocardial Ischemia (MI)	1	hsa-miR-218-5p; hsa-miR-106a-5p; hsa-miR-642a-5p; hsa-let-7c-5p; hsa-miR-143-3p; hsa-miR-331-3p	6
5465	*PPARA*	Hypertensive disease; Inflammation	2	hsa-miR-218-5p; hsa-miR-185-5p; hsa-miR-106a-5p; hsa-let-7c-5p; hsa-miR-331-3p; hsa-miR-143-3p	6
7145	*TNS1*	Asthma	1	hsa-miR-185-5p; hsa-miR-106a-5p; hsa-miR-218-5p; hsa-miR-331-3p; hsa-miR-143-3p; hsa-miR-642a-5p	6
463	*ZFHX3*	Atrial Fibrillation	1	hsa-miR-652-3p; hsa-miR-218-5p; hsa-miR-106a-5p; hsa-miR-642a-5p; hsa-miR-143-3p; hsa-miR-331-3p	6
5139	*PDE3A*	Thrombosis; Myocardial Infarction	2	hsa-miR-185-5p; hsa-miR-106a-5p; hsa-miR-218-5p; hsa-miR-652-3p; hsa-miR-143-3p; hsa-miR-642a-5p	6
55973	*BCAP29*	Coronary heart disease	1	hsa-miR-218-5p; hsa-miR-185-5p; hsa-miR-106a-5p; hsa-let-7c-5p; hsa-miR-331-3p; hsa-miR-143-3p	6
10021	*HCN4*	Atrial Fibrillation	1	hsa-miR-642a-5p; hsa-miR-331-3p; hsa-miR-143-3p; hsa-miR-218-5p; hsa-miR-185-5p; hsa-miR-106a-5p	6
8490	*RGS5*	Hypertensive disease	1	hsa-miR-642a-5p; hsa-miR-331-3p; hsa-miR-143-3p; hsa-miR-652-3p; hsa-miR-185-5p; hsa-miR-106a-5p	6
2526	*FUT4*	Thrombosis	1	hsa-miR-106a-5p; hsa-miR-185-5p; hsa-miR-331-3p; hsa-miR-143-3p; hsa-miR-642a-5p; hsa-let-7c-5p	6
367	*AR*	Hypertensive disease	1	hsa-let-7c-5p; hsa-miR-642a-5p; hsa-miR-143-3p; hsa-miR-218-5p; hsa-miR-185-5p; hsa-miR-106a-5p	6
10451	*VAV3*	Hypertensive disease; Asthma	2	hsa-miR-106a-5p; hsa-miR-652-3p; hsa-miR-218-5p; hsa-miR-143-3p; hsa-let-7c-5p; hsa-miR-642a-5p	6

Entrez ID	Gene	CVDs (DisGeNET db v4.0)	CVD number	EV-miRNA targets	EV-miRNA number
3099	*HK2*	Myocardial Ischemia (MI)	1	hsa-miR-106a-5p; hsa-miR-185-5p; hsa-miR-218-5p; hsa-miR-143-3p; hsa-miR-642a-5p; hsa-let-7c-5p	6
5054	*SERPINE1*	Hypertensive disease; Thrombosis; Heart failure	3	hsa-miR-185-5p; hsa-miR-106a-5p; hsa-miR-218-5p; hsa-miR-143-3p; hsa-miR-331-3p; hsa-let-7c-5p	6
10928	*RALBP1*	Myocardial Ischemia (MI); Hypertensive disease	2	hsa-miR-218-5p; hsa-miR-106a-5p; hsa-miR-185-5p; hsa-miR-642a-5p; hsa-let-7c-5p; hsa-miR-143-3p	6
5069	*PAPPA*	Myocardial Infarction	1	hsa-miR-106a-5p; hsa-miR-185-5p; hsa-miR-652-3p; hsa-miR-143-3p; hsa-miR-642a-5p; hsa-let-7c-5p	6
6581	*SLC22A3*	Coronary heart disease	1	hsa-miR-218-5p; hsa-miR-185-5p; hsa-miR-106a-5p; hsa-let-7c-5p; hsa-miR-143-3p; hsa-miR-331-3p	6
23327	*NEDD4L*	Hypertensive disease	1	hsa-miR-106a-5p; hsa-miR-185-5p; hsa-miR-642a-5p; hsa-let-7c-5p; hsa-miR-143-3p; hsa-miR-331-3p	6
11127	*KIF3A*	Asthma	1	hsa-let-7c-5p; hsa-miR-331-3p; hsa-miR-143-3p; hsa-miR-218-5p; hsa-miR-185-5p; hsa-miR-106a-5p	6
6387	*CXCL12*	Ischemia	1	hsa-miR-652-3p; hsa-miR-106a-5p; hsa-miR-185-5p; hsa-miR-642a-5p; hsa-let-7c-5p; hsa-miR-143-3p	6
5502	*PPP1R1A*	Cardiac Arrhythmia; Heart failure	2	hsa-let-7c-5p; hsa-miR-642a-5p; hsa-miR-143-3p; hsa-miR-331-3p; hsa-miR-185-5p; hsa-miR-106a-5p	6
595	*CCND1*	Myocardial Ischemia (MI)	1	hsa-miR-218-5p; hsa-miR-185-5p; hsa-miR-106a-5p; hsa-let-7c-5p; hsa-miR-642a-5p; hsa-miR-143-3p	6
488	*ATP2A2*	Heart Diseases; Heart failure	2	hsa-miR-218-5p; hsa-miR-106a-5p; hsa-miR-185-5p; hsa-let-7c-5p; hsa-miR-143-3p; hsa-miR-331-3p	6
22808	*MRAS*	Coronary heart disease	1	hsa-miR-143-3p; hsa-miR-331-3p; hsa-let-7c-5p; hsa-miR-642a-5p; hsa-miR-185-5p; hsa-miR-106a-5p	6
7224	*TRPC5*	Hypertensive disease	1	hsa-miR-106a-5p; hsa-miR-185-5p; hsa-miR-218-5p; hsa-miR-143-3p; hsa-miR-642a-5p; hsa-let-7c-5p	6
7091	*TLE4*	Myocardial Ischemia (MI)	1	hsa-let-7c-5p; hsa-miR-99b-5p; hsa-miR-218-5p; hsa-miR-652-3p; hsa-miR-185-5p; hsa-miR-106a-5p	6
57623	*ZFAT*	Hypertensive disease	1	hsa-let-7c-5p; hsa-miR-642a-5p; hsa-miR-143-3p; hsa-miR-99b-5p; hsa-miR-218-5p; hsa-miR-652-3p	6
64375	*IKZF4*	Asthma	1	hsa-let-7c-5p; hsa-miR-642a-5p; hsa-miR-331-3p; hsa-miR-	6

Entrez ID	Gene	CVDs (DisGeNET db v4.0)	CVD number	EV-miRNA targets	EV-miRNA number
				99b-5p; hsa-miR-185-5p; hsa-miR-106a-5p	
2908	*NR3C1*	Hypertensive disease	1	hsa-miR-642a-5p; hsa-miR-143-3p; hsa-miR-218-5p; hsa-miR-652-3p; hsa-miR-106a-5p; hsa-miR-185-5p	6
150	*ADRA2A*	Hypertensive disease; Heart Diseases	2	hsa-miR-106a-5p; hsa-miR-185-5p; hsa-miR-218-5p; hsa-miR-652-3p; hsa-miR-331-3p; hsa-let-7c-5p	6
1277	*COL1A1*	Hypertensive disease	1	hsa-miR-106a-5p; hsa-miR-185-5p; hsa-miR-218-5p; hsa-miR-143-3p; hsa-miR-642a-5p; hsa-let-7c-5p	6
7132	*TNFRSF1A*	Heart failure	1	hsa-miR-652-3p; hsa-miR-218-5p; hsa-miR-106a-5p; hsa-miR-642a-5p; hsa-miR-331-3p	5
5420	*PODXL*	Thrombosis	1	hsa-miR-185-5p; hsa-miR-331-3p; hsa-miR-143-3p; hsa-miR-99b-5p; hsa-miR-642a-5p	5
153	*ADRB1*	Myocardial Ischemia (MI); Cardiovascular Diseases; Heart failure; Hypertensive disease	4	hsa-let-7c-5p; hsa-miR-642a-5p; hsa-miR-331-3p; hsa-miR-218-5p; hsa-miR-185-5p	5
7037	*TFRC*	Inflammation; Myocardial Ischemia (MI)	2	hsa-miR-143-3p; hsa-miR-642a-5p; hsa-let-7c-5p; hsa-miR-185-5p; hsa-miR-218-5p	5
10050	*SLC17A4*	Cardiovascular Diseases	1	hsa-miR-185-5p; hsa-miR-106a-5p; hsa-miR-218-5p; hsa-miR-331-3p; hsa-let-7c-5p	5
27020	*NPTN*	Hypertensive disease; Myocardial Ischemia (MI)	2	hsa-miR-642a-5p; hsa-miR-218-5p; hsa-miR-652-3p; hsa-miR-106a-5p; hsa-miR-185-5p	5
65266	*WNK4*	Hypertensive disease	1	hsa-miR-218-5p; hsa-miR-185-5p; hsa-miR-642a-5p; hsa-miR-331-3p; hsa-miR-143-3p	5
2982	*GUCY1A3*	Hypertensive disease; Thrombosis	2	hsa-miR-218-5p; hsa-miR-106a-5p; hsa-miR-185-5p; hsa-miR-99b-5p; hsa-miR-331-3p	5
286046	*XKR6*	Asthma	1	hsa-miR-143-3p; hsa-miR-642a-5p; hsa-miR-185-5p; hsa-miR-218-5p; hsa-miR-652-3p	5
153090	*DAB2IP*	Myocardial Infarction	1	hsa-miR-185-5p; hsa-miR-106a-5p; hsa-miR-642a-5p; hsa-miR-143-3p; hsa-miR-331-3p	5
10611	*PDLIM5*	Myocardial Ischemia (MI)	1	hsa-miR-106a-5p; hsa-miR-185-5p; hsa-miR-642a-5p; hsa-let-7c-5p; hsa-miR-143-3p	5

Entrez ID	Gene	CVDs (DisGeNET db v4.0)	CVD number	EV-miRNA targets	EV-miRNA number
3690	*ITGB3*	Myocardial Infarction; Cerebral Hemorrhage	2	hsa-miR-642a-5p; hsa-let-7c-5p; hsa-miR-331-3p; hsa-miR-106a-5p; hsa-miR-185-5p	5
2932	*GSK3B*	Myocardial Infarction; Heart failure	2	hsa-miR-185-5p; hsa-miR-106a-5p; hsa-miR-218-5p; hsa-miR-331-3p; hsa-miR-143-3p	5
775	*CACNA1C*	Hypertensive disease; Cardiac Arrhythmia	2	hsa-miR-185-5p; hsa-miR-218-5p; hsa-miR-331-3p; hsa-miR-143-3p; hsa-miR-642a-5p	5
51752	*ERAP1*	Hypertensive disease	1	hsa-miR-218-5p; hsa-miR-185-5p; hsa-miR-106a-5p; hsa-miR-143-3p; hsa-miR-331-3p	5
334	*APLP2*	Myocardial Ischemia (MI)	1	hsa-miR-99b-5p; hsa-miR-331-3p; hsa-miR-642a-5p; hsa-miR-185-5p; hsa-miR-218-5p	5
91543	*RSAD2*	Myocardial Ischemia (MI)	1	hsa-let-7c-5p; hsa-miR-642a-5p; hsa-miR-143-3p; hsa-miR-185-5p; hsa-miR-106a-5p	5
2643	*GCH1*	Myocardial Ischemia (MI); Hypertensive disease	2	hsa-miR-106a-5p; hsa-miR-218-5p; hsa-miR-652-3p; hsa-let-7c-5p; hsa-miR-642a-5p	5
1728	*NQO1*	Asthma	1	hsa-miR-185-5p; hsa-miR-218-5p; hsa-miR-143-3p; hsa-miR-642a-5p; hsa-let-7c-5p	5
8809	*IL18R1*	Asthma	1	hsa-miR-185-5p; hsa-miR-106a-5p; hsa-miR-652-3p; hsa-miR-331-3p; hsa-let-7c-5p	5
3953	*LEPR*	Hypertensive disease	1	hsa-miR-218-5p; hsa-miR-185-5p; hsa-miR-106a-5p; hsa-let-7c-5p; hsa-miR-143-3p	5
3077	*HFE*	Cardiovascular Diseases; Hypertensive disease	2	hsa-miR-185-5p; hsa-miR-106a-5p; hsa-miR-143-3p; hsa-let-7c-5p; hsa-miR-642a-5p	5
2768	*GNA12*	Myocardial Ischemia (MI)	1	hsa-miR-218-5p; hsa-miR-106a-5p; hsa-miR-185-5p; hsa-miR-642a-5p; hsa-miR-331-3p	5
4088	*SMAD3*	Coronary heart disease; Asthma	2	hsa-miR-143-3p; hsa-miR-642a-5p; hsa-miR-185-5p; hsa-miR-106a-5p; hsa-miR-218-5p	5
490	*ATP2B1*	Coronary heart disease; Hypertensive disease	2	hsa-miR-642a-5p; hsa-let-7c-5p; hsa-miR-106a-5p; hsa-miR-185-5p; hsa-miR-652-3p	5
5739	*PTGIR*	Heart Diseases	1	hsa-miR-185-5p; hsa-miR-331-3p; hsa-miR-143-3p; hsa-miR-642a-5p; hsa-let-7c-5p	5
114818	*KLHL29*	Hypertensive disease	1	hsa-miR-218-5p; hsa-miR-185-5p; hsa-let-7c-5p; hsa-miR-	5

Entrez ID	Gene	CVDs (DisGeNET db v4.0)	CVD number	EV-miRNA targets	EV-miRNA number
				642a-5p; hsa-miR-331-3p	
5663	*PSEN1*	Heart failure	1	hsa-miR-143-3p; hsa-miR-652-3p; hsa-miR-218-5p; hsa-miR-185-5p; hsa-miR-106a-5p	5
2321	*FLT1*	Cerebral Hemorrhage; Coronary heart disease	2	hsa-miR-331-3p; hsa-miR-143-3p; hsa-miR-218-5p; hsa-miR-185-5p; hsa-miR-106a-5p	5
3371	*TNC*	Asthma	1	hsa-miR-218-5p; hsa-miR-106a-5p; hsa-miR-185-5p; hsa-miR-642a-5p; hsa-miR-331-3p	5
10052	*GJC1*	Hypertensive disease	1	hsa-let-7c-5p; hsa-miR-143-3p; hsa-miR-331-3p; hsa-miR-652-3p; hsa-miR-106a-5p	5
5601	*MAPK9*	Ischemia	1	hsa-miR-143-3p; hsa-let-7c-5p; hsa-miR-185-5p; hsa-miR-106a-5p; hsa-miR-218-5p	5
94103	*ORMDL3*	Asthma	1	hsa-miR-218-5p; hsa-miR-106a-5p; hsa-miR-642a-5p; hsa-miR-143-3p; hsa-miR-331-3p	5
3572	*IL6ST*	Myocardial Ischemia (MI)	1	hsa-miR-652-3p; hsa-miR-185-5p; hsa-miR-106a-5p; hsa-miR-642a-5p; hsa-miR-143-3p	5
358	*AQP1*	Hypertensive disease	1	hsa-miR-185-5p; hsa-miR-331-3p; hsa-miR-143-3p; hsa-let-7c-5p; hsa-miR-642a-5p	5
9021	*SOCS3*	Myocardial Ischemia (MI)	1	hsa-let-7c-5p; hsa-miR-642a-5p; hsa-miR-331-3p; hsa-miR-218-5p; hsa-miR-185-5p	5
8989	*TRPA1*	Asthma; Inflammation	2	hsa-miR-218-5p; hsa-miR-185-5p; hsa-miR-106a-5p; hsa-let-7c-5p; hsa-miR-143-3p	5
2784	*GNB3*	Hypertensive disease	1	hsa-miR-652-3p; hsa-miR-185-5p; hsa-let-7c-5p; hsa-miR-642a-5p; hsa-miR-143-3p	5
6869	*TACR1*	Hypertensive disease	1	hsa-miR-106a-5p; hsa-miR-185-5p; hsa-miR-642a-5p; hsa-miR-143-3p; hsa-miR-331-3p	5
6943	*TCF21*	Coronary heart disease	1	hsa-miR-106a-5p; hsa-miR-185-5p; hsa-miR-642a-5p; hsa-let-7c-5p; hsa-miR-143-3p	5
7056	*THBD*	Myocardial Infarction; Thrombosis	2	hsa-miR-106a-5p; hsa-miR-185-5p; hsa-miR-143-3p; hsa-miR-642a-5p; hsa-let-7c-5p	5
5089	*PBX2*	Asthma	1	hsa-miR-218-5p; hsa-miR-185-5p; hsa-let-7c-5p; hsa-miR-642a-5p; hsa-miR-331-3p	5

Entrez ID	Gene	CVDs (DisGeNET db v4.0)	CVD number	EV-miRNA targets	EV-miRNA number
2066	*ERBB4*	Asthma	1	hsa-miR-218-5p; hsa-miR-106a-5p; hsa-miR-642a-5p; hsa-miR-331-3p; hsa-miR-143-3p	5
1737	*DLAT*	Myocardial Ischemia (MI)	1	hsa-miR-143-3p; hsa-let-7c-5p; hsa-miR-642a-5p; hsa-miR-185-5p; hsa-miR-106a-5p	5
4524	*MTHFR*	Atrial Fibrillation; Coronary heart disease; Cardiovascular Diseases	3	hsa-miR-642a-5p; hsa-let-7c-5p; hsa-miR-143-3p; hsa-miR-106a-5p; hsa-miR-185-5p	5
41	*ASIC1*	Inflammation	1	hsa-miR-218-5p; hsa-miR-185-5p; hsa-let-7c-5p; hsa-miR-642a-5p; hsa-miR-331-3p	5
3782	*KCNN3*	Atrial Fibrillation	1	hsa-miR-218-5p; hsa-miR-652-3p; hsa-miR-185-5p; hsa-miR-143-3p; hsa-miR-331-3p	5
2702	*GJA5*	Hypertensive disease	1	hsa-miR-652-3p; hsa-miR-185-5p; hsa-miR-642a-5p; hsa-let-7c-5p; hsa-miR-143-3p	5
3949	*LDLR*	Coronary heart disease; Cardiovascular Diseases	2	hsa-miR-106a-5p; hsa-miR-185-5p; hsa-miR-143-3p; hsa-miR-642a-5p; hsa-let-7c-5p	5
81876	*RAB1B*	Myocardial Ischemia (MI)	1	hsa-miR-185-5p; hsa-miR-218-5p; hsa-miR-331-3p; hsa-miR-642a-5p; hsa-let-7c-5p	5
23316	*CUX2*	Coronary heart disease	1	hsa-let-7c-5p; hsa-miR-218-5p; hsa-miR-652-3p; hsa-miR-106a-5p; hsa-miR-185-5p	5
3479	*IGF1*	Hypertensive disease; Myocardial Ischemia (MI)	2	hsa-let-7c-5p; hsa-miR-143-3p; hsa-miR-331-3p; hsa-miR-218-5p; hsa-miR-185-5p	5
6774	*STAT3*	Inflammation	1	hsa-miR-143-3p; hsa-miR-331-3p; hsa-miR-642a-5p; hsa-let-7c-5p; hsa-miR-106a-5p	5
2099	*ESR1*	Myocardial Infarction	1	hsa-miR-652-3p; hsa-miR-218-5p; hsa-miR-106a-5p; hsa-miR-642a-5p; hsa-miR-331-3p	5
23108	*RAP1GAP2*	Asthma	1	hsa-miR-143-3p; hsa-miR-642a-5p; hsa-miR-185-5p; hsa-miR-106a-5p; hsa-miR-218-5p	5
3952	*LEP*	Inflammation; Hypertensive disease	2	hsa-miR-106a-5p; hsa-miR-185-5p; hsa-miR-218-5p; hsa-miR-331-3p; hsa-miR-642a-5p	5
22919	*MAPRE1*	Myocardial Ischemia (MI)	1	hsa-let-7c-5p; hsa-miR-143-3p; hsa-miR-652-3p; hsa-miR-185-5p; hsa-miR-106a-5p	5

Entrez ID	Gene	CVDs (DisGeNET db v4.0)	CVD number	EV-miRNA targets	EV-miRNA number
5170	*PDPK1*	Heart failure	1	hsa-miR-106a-5p; hsa-miR-185-5p; hsa-miR-331-3p; hsa-miR-143-3p; hsa-miR-642a-5p	5
3570	*IL6R*	Coronary heart disease; Asthma	2	hsa-let-7c-5p; hsa-miR-331-3p; hsa-miR-143-3p; hsa-miR-106a-5p; hsa-miR-185-5p	5
22806	*IKZF3*	Asthma	1	hsa-miR-143-3p; hsa-let-7c-5p; hsa-miR-106a-5p; hsa-miR-185-5p; hsa-miR-652-3p	5
1536	*CYBB*	Heart failure; Hypertensive disease	2	hsa-miR-143-3p; hsa-miR-642a-5p; hsa-let-7c-5p; hsa-miR-106a-5p; hsa-miR-185-5p	5
222256	*CDHR3*	Asthma	1	hsa-miR-106a-5p; hsa-miR-185-5p; hsa-miR-642a-5p; hsa-let-7c-5p; hsa-miR-143-3p	5
7077	*TIMP2*	Myocardial Infarction	1	hsa-miR-106a-5p; hsa-miR-185-5p; hsa-miR-218-5p; hsa-miR-642a-5p	4
283450	*HECTD4*	Coronary heart disease; Hypertensive disease	2	hsa-miR-331-3p; hsa-let-7c-5p; hsa-miR-106a-5p; hsa-miR-218-5p	4
4306	*NR3C2*	Myocardial Infarction; Heart failure	2	hsa-miR-185-5p; hsa-miR-106a-5p; hsa-let-7c-5p; hsa-miR-642a-5p	4
6548	*SLC9A1*	Heart failure	1	hsa-miR-185-5p; hsa-let-7c-5p; hsa-miR-331-3p; hsa-miR-143-3p	4
5868	*RAB5A*	Myocardial Ischemia (MI)	1	hsa-miR-106a-5p; hsa-miR-185-5p; hsa-miR-143-3p; hsa-miR-331-3p	4
4023	*LPL*	Hypertensive disease; Cardiovascular Diseases; Coronary heart disease	3	hsa-miR-218-5p; hsa-miR-185-5p; hsa-miR-642a-5p; hsa-miR-143-3p	4
6546	*SLC8A1*	Myocardial Ischemia (MI)	1	hsa-miR-331-3p; hsa-miR-218-5p; hsa-miR-106a-5p; hsa-miR-185-5p	4
155	*ADRB3*	Heart failure	1	hsa-miR-642a-5p; hsa-let-7c-5p; hsa-miR-218-5p; hsa-miR-106a-5p	4
2549	*GAB1*	Asthma	1	hsa-miR-106a-5p; hsa-miR-218-5p; hsa-miR-143-3p; hsa-miR-642a-5p	4
7421	*VDR*	Hypertensive disease	1	hsa-miR-185-5p; hsa-miR-331-3p; hsa-let-7c-5p; hsa-miR-642a-5p	4

Entrez ID	Gene	CVDs (DisGeNET db v4.0)	CVD number	EV-miRNA targets	EV-miRNA number
6660	*SOX5*	Atrial Fibrillation	1	hsa-miR-642a-5p; hsa-miR-143-3p; hsa-miR-218-5p; hsa-miR-185-5p	4
134430	*WDR36*	Asthma	1	hsa-miR-143-3p; hsa-miR-642a-5p; hsa-miR-106a-5p; hsa-miR-185-5p	4
5396	*PRRX1*	Atrial Fibrillation	1	hsa-miR-652-3p; hsa-miR-106a-5p; hsa-miR-642a-5p; hsa-let-7c-5p	4
83939	*EIF2A*	Myocardial Ischemia (MI)	1	hsa-miR-185-5p; hsa-miR-218-5p; hsa-miR-652-3p; hsa-miR-143-3p	4
492	*ATP2B3*	Hypertensive disease	1	hsa-miR-652-3p; hsa-miR-106a-5p; hsa-miR-642a-5p; hsa-let-7c-5p	4
6678	*SPARC*	Myocardial Ischemia (MI)	1	hsa-miR-652-3p; hsa-miR-185-5p; hsa-miR-106a-5p; hsa-miR-143-3p	4
84909	*C9orf3*	Atrial Fibrillation	1	hsa-miR-106a-5p; hsa-miR-185-5p; hsa-miR-652-3p; hsa-miR-143-3p	4
3360	*HTR4*	Cardiac Arrhythmia; Atrial Fibrillation	2	hsa-let-7c-5p; hsa-miR-642a-5p; hsa-miR-331-3p; hsa-miR-143-3p	4
5142	*PDE4B*	Myocardial Ischemia (MI); Asthma	2	hsa-miR-331-3p; hsa-let-7c-5p; hsa-miR-106a-5p; hsa-miR-652-3p	4
3586	*IL10*	Myocardial Infarction; Inflammation	2	hsa-miR-642a-5p; hsa-let-7c-5p; hsa-miR-106a-5p; hsa-miR-185-5p	4
60495	*HPSE2*	Asthma	1	hsa-miR-218-5p; hsa-miR-185-5p; hsa-miR-642a-5p; hsa-miR-331-3p	4
5495	*PPM1B*	Myocardial Ischemia (MI)	1	hsa-miR-642a-5p; hsa-miR-143-3p; hsa-miR-185-5p; hsa-miR-106a-5p	4
5581	*PRKCE*	Myocardial Infarction; Myocardial Ischemia (MI)	2	hsa-miR-218-5p; hsa-miR-642a-5p; hsa-miR-143-3p; hsa-miR-331-3p	4
1385	*CREB1*	Myocardial Infarction	1	hsa-miR-642a-5p; hsa-miR-143-3p; hsa-miR-218-5p; hsa-miR-106a-5p	4
1889	*ECE1*	Hypertensive disease	1	hsa-miR-331-3p; hsa-miR-642a-5p; hsa-miR-106a-5p; hsa-miR-218-5p	4
829	*CAPZA1*	Hypertensive disease	1	hsa-miR-143-3p; hsa-let-7c-5p; hsa-miR-106a-5p; hsa-miR-	4

Entrez ID	Gene	CVDs (DisGeNET db v4.0)	CVD number	EV-miRNA targets	EV-miRNA number
				185-5p	
1432	*MAPK14*	Myocardial Ischemia (MI)	1	hsa-miR-652-3p; hsa-miR-185-5p; hsa-miR-642a-5p; hsa-miR-143-3p	4
6198	*RPS6KB1*	Hypertensive disease	1	hsa-miR-106a-5p; hsa-miR-218-5p; hsa-miR-331-3p; hsa-miR-642a-5p	4
9948	*WDR1*	Inflammation	1	hsa-miR-331-3p; hsa-miR-642a-5p; hsa-miR-106a-5p; hsa-miR-218-5p	4
5916	*RARG*	Heart Diseases	1	hsa-miR-652-3p; hsa-miR-185-5p; hsa-miR-331-3p; hsa-miR-143-3p	4
6927	*HNF1A*	Coronary heart disease	1	hsa-miR-642a-5p; hsa-miR-331-3p; hsa-miR-218-5p; hsa-miR-106a-5p	4
5609	*MAP2K7*	Heart failure	1	hsa-miR-642a-5p; hsa-let-7c-5p; hsa-miR-331-3p; hsa-miR-185-5p	4
6340	*SCNN1G*	Hypertensive disease	1	hsa-miR-185-5p; hsa-miR-331-3p; hsa-miR-99b-5p; hsa-miR-642a-5p	4
27347	*STK39*	Hypertensive disease	1	hsa-miR-185-5p; hsa-miR-642a-5p; hsa-let-7c-5p; hsa-miR-143-3p	4
55351	*STK32B*	Coronary heart disease	1	hsa-miR-106a-5p; hsa-miR-185-5p; hsa-miR-143-3p; hsa-miR-331-3p	4
1490	*CTGF*	Heart failure; Hypertensive disease	2	hsa-miR-218-5p; hsa-miR-106a-5p; hsa-miR-185-5p; hsa-miR-143-3p	4
9966	*TNFSF15*	Inflammation	1	hsa-miR-652-3p; hsa-miR-331-3p; hsa-miR-143-3p; hsa-miR-642a-5p	4
2697	*GJA1*	Cardiac Arrhythmia; Hypertensive disease	2	hsa-miR-106a-5p; hsa-miR-185-5p; hsa-miR-218-5p; hsa-miR-642a-5p	4
4602	*MYB*	Asthma	1	hsa-miR-185-5p; hsa-miR-106a-5p; hsa-miR-331-3p; hsa-let-7c-5p	4
6530	*SLC6A2*	Hypertensive disease	1	hsa-let-7c-5p; hsa-miR-331-3p; hsa-miR-218-5p; hsa-miR-185-5p	4
3309	*HSPA5*	Myocardial Infarction	1	hsa-miR-642a-5p; hsa-let-7c-5p; hsa-miR-106a-5p; hsa-miR-185-5p	4

Entrez ID	Gene	CVDs (DisGeNET db v4.0)	CVD number	EV-miRNA targets	EV-miRNA number
3685	*ITGAV*	Cerebral Hemorrhage	1	hsa-let-7c-5p; hsa-miR-143-3p; hsa-miR-218-5p; hsa-miR-106a-5p	4
9173	*IL1RL1*	Asthma	1	hsa-miR-143-3p; hsa-miR-185-5p; hsa-miR-106a-5p; hsa-miR-652-3p	4
4734	*NEDD4*	Myocardial Ischemia (MI)	1	hsa-miR-143-3p; hsa-let-7c-5p; hsa-miR-106a-5p; hsa-miR-652-3p	4
4615	*MYD88*	Inflammation	1	hsa-miR-642a-5p; hsa-miR-143-3p; hsa-miR-218-5p; hsa-miR-185-5p	4
8841	*HDAC3*	Heart Diseases	1	hsa-miR-99b-5p; hsa-miR-642a-5p; hsa-miR-185-5p; hsa-miR-106a-5p	4
4363	*ABCC1*	Hypertensive disease; Heart Diseases	2	hsa-let-7c-5p; hsa-miR-331-3p; hsa-miR-143-3p; hsa-miR-185-5p	4
7112	*TMPO*	Myocardial Ischemia (MI)	1	hsa-miR-143-3p; hsa-let-7c-5p; hsa-miR-106a-5p; hsa-miR-218-5p	4
6925	*TCF4*	Heart Diseases	1	hsa-miR-642a-5p; hsa-miR-185-5p; hsa-miR-106a-5p; hsa-miR-218-5p	4
6331	*SCN5A*	Atrial Fibrillation	1	hsa-miR-331-3p; hsa-let-7c-5p; hsa-miR-185-5p; hsa-miR-218-5p	4
6776	*STAT5A*	Myocardial Ischemia (MI)	1	hsa-miR-185-5p; hsa-miR-642a-5p; hsa-miR-331-3p; hsa-miR-143-3p	4
5534	*PPP3R1*	Myocardial Ischemia (MI)	1	hsa-miR-143-3p; hsa-let-7c-5p; hsa-miR-106a-5p; hsa-miR-185-5p	4
84624	*FNDC1*	Coronary heart disease	1	hsa-miR-185-5p; hsa-miR-218-5p; hsa-miR-143-3p; hsa-miR-331-3p	4
3162	*HMOX1*	Heart failure; Cerebral Hemorrhage; Ischemia; Inflammation; Asthma; Thrombosis; Hypertensive disease; Myocardial Ischemia (MI)	8	hsa-miR-642a-5p; hsa-miR-185-5p; hsa-miR-106a-5p; hsa-miR-218-5p	4
54205	*CYCS*	Ischemia	1	hsa-miR-143-3p; hsa-miR-218-5p; hsa-miR-652-3p; hsa-miR-	4

Entrez ID	Gene	CVDs (DisGeNET db v4.0)	CVD number	EV-miRNA targets	EV-miRNA number
				106a-5p	
375056	*MIA3*	Coronary heart disease; Myocardial Infarction	2	hsa-miR-143-3p; hsa-miR-106a-5p; hsa-miR-185-5p; hsa-miR-652-3p	4
221458	*KIF6*	Myocardial Infarction	1	hsa-miR-143-3p; hsa-miR-331-3p; hsa-miR-218-5p; hsa-miR-185-5p	4
5592	*PRKG1*	Asthma	1	hsa-miR-218-5p; hsa-miR-106a-5p; hsa-miR-642a-5p; hsa-miR-143-3p	4
23621	*BACE1*	Cardiovascular Diseases	1	hsa-miR-642a-5p; hsa-miR-143-3p; hsa-miR-331-3p; hsa-miR-185-5p	4
7879	*RAB7A*	Myocardial Ischemia (MI)	1	hsa-miR-331-3p; hsa-miR-143-3p; hsa-miR-218-5p; hsa-miR-185-5p	4
3759	*KCNJ2*	Cardiac Arrhythmia; Atrial Fibrillation	2	hsa-let-7c-5p; hsa-miR-642a-5p; hsa-miR-652-3p; hsa-miR-218-5p	4
151648	*SGOL1*	Cardiac Arrhythmia	1	hsa-miR-106a-5p; hsa-miR-185-5p; hsa-let-7c-5p; hsa-miR-143-3p	4
9370	*ADIPOQ*	Myocardial Ischemia (MI); Inflammation; Hypertensive disease; Heart failure	4	hsa-miR-143-3p; hsa-miR-642a-5p; hsa-let-7c-5p; hsa-miR-185-5p	4
5294	*PIK3CG*	Heart failure	1	hsa-let-7c-5p; hsa-miR-143-3p; hsa-miR-218-5p; hsa-miR-185-5p	4
3560	*IL2RB*	Asthma	1	hsa-let-7c-5p; hsa-miR-642a-5p; hsa-miR-331-3p; hsa-miR-185-5p	4
5471	*PPAT*	Myocardial Ischemia (MI)	1	hsa-miR-106a-5p; hsa-miR-185-5p; hsa-let-7c-5p; hsa-miR-143-3p	4
3764	*KCNJ8*	Myocardial Ischemia (MI)	1	hsa-miR-106a-5p; hsa-miR-185-5p; hsa-miR-218-5p; hsa-miR-143-3p	4
4158	*MC2R*	Hypertensive disease	1	hsa-miR-642a-5p; hsa-let-7c-5p; hsa-miR-185-5p; hsa-miR-218-5p	4
817	*CAMK2D*	Myocardial Ischemia (MI)	1	hsa-miR-106a-5p; hsa-miR-185-5p; hsa-miR-652-3p; hsa-miR-143-3p	4
23491	*CES3*	Myocardial Ischemia (MI)	1	hsa-let-7c-5p; hsa-miR-331-3p; hsa-miR-218-5p; hsa-miR-185-	4

Entrez ID	Gene	CVDs (DisGeNET db v4.0)	CVD number	EV-miRNA targets	EV-miRNA number
				5p	
4973	*OLR1*	Myocardial Infarction; Hypertensive disease; Heart failure	3	hsa-miR-106a-5p; hsa-miR-185-5p; hsa-miR-143-3p; hsa-let-7c-5p	4
1909	*EDNRA*	Hypertensive disease	1	hsa-let-7c-5p; hsa-miR-106a-5p; hsa-miR-185-5p; hsa-miR-218-5p	4
2628	*GATM*	Myocardial Ischemia (MI); Heart failure	2	hsa-miR-331-3p; hsa-miR-143-3p; hsa-let-7c-5p; hsa-miR-185-5p	4
1959	*EGR2*	Myocardial Ischemia (MI)	1	hsa-miR-218-5p; hsa-miR-185-5p; hsa-miR-106a-5p; hsa-miR-642a-5p	4
598	*BCL2L1*	Myocardial Infarction; Cerebral Hemorrhage	2	hsa-miR-642a-5p; hsa-let-7c-5p; hsa-miR-331-3p; hsa-miR-185-5p	4
7052	*TGM2*	Hypertensive disease	1	hsa-miR-143-3p; hsa-miR-331-3p; hsa-miR-185-5p; hsa-miR-106a-5p	4
3176	*HNMT*	Asthma	1	hsa-miR-218-5p; hsa-miR-106a-5p; hsa-miR-143-3p; hsa-miR-331-3p	4
65264	*UBE2Z*	Coronary heart disease	1	hsa-miR-185-5p; hsa-miR-106a-5p; hsa-miR-331-3p; hsa-miR-642a-5p	4
540	*ATP7B*	Inflammation	1	hsa-let-7c-5p; hsa-miR-331-3p; hsa-miR-185-5p; hsa-miR-106a-5p	4
3576	*CXCL8*	Inflammation	1	hsa-let-7c-5p; hsa-miR-143-3p; hsa-miR-106a-5p; hsa-miR-185-5p	4
5834	*PYGB*	Myocardial Ischemia (MI)	1	hsa-miR-99b-5p; hsa-miR-331-3p; hsa-let-7c-5p; hsa-miR-106a-5p	4
2246	*FGF1*	Myocardial Ischemia (MI)	1	hsa-miR-642a-5p; hsa-miR-143-3p; hsa-miR-652-3p	3
10891	*PPARGC1A*	Myocardial Infarction; Heart failure	2	hsa-miR-642a-5p; hsa-let-7c-5p; hsa-miR-218-5p	3
3791	*KDR*	Myocardial Ischemia (MI); Cerebral Hemorrhage	2	hsa-miR-143-3p; hsa-miR-106a-5p; hsa-miR-185-5p	3
59272	*ACE2*	Hypertensive disease	1	hsa-miR-218-5p; hsa-miR-331-3p; hsa-miR-143-3p	3
1326	*MAP3K8*	Myocardial Ischemia (MI)	1	hsa-miR-143-3p; hsa-miR-652-3p; hsa-miR-106a-5p	3

Entrez ID	Gene	CVDs (DisGeNET db v4.0)	CVD number	EV-miRNA targets	EV-miRNA number
3486	*IGFBP3*	Myocardial Ischemia (MI)	1	hsa-miR-185-5p; hsa-miR-218-5p; hsa-miR-143-3p	3
836	*CASP3*	Cerebral Hemorrhage; Myocardial Infarction	2	hsa-let-7c-5p; hsa-miR-652-3p; hsa-miR-106a-5p	3
8870	*IER3*	Hypertensive disease	1	hsa-miR-106a-5p; hsa-miR-143-3p; hsa-let-7c-5p	3
111	*ADCY5*	Hypertensive disease	1	hsa-miR-642a-5p; hsa-miR-143-3p; hsa-miR-331-3p	3
40	*ASIC2*	Inflammation	1	hsa-miR-185-5p; hsa-miR-642a-5p; hsa-miR-143-3p	3
6337	*SCNN1A*	Hypertensive disease	1	hsa-miR-185-5p; hsa-miR-143-3p; hsa-miR-642a-5p	3
5742	*PTGS1*	Heart failure	1	hsa-miR-642a-5p; hsa-miR-331-3p; hsa-miR-106a-5p	3
126393	*HSPB6*	Heart Diseases	1	hsa-miR-218-5p; hsa-miR-106a-5p; hsa-miR-185-5p	3
10959	*TMED2*	Myocardial Ischemia (MI)	1	hsa-miR-143-3p; hsa-miR-218-5p; hsa-miR-106a-5p	3
5728	*PTEN*	Asthma; Hypertensive disease	2	hsa-miR-642a-5p; hsa-miR-143-3p; hsa-miR-106a-5p	3
5175	*PECAM1*	Coronary heart disease	1	hsa-miR-143-3p; hsa-miR-218-5p; hsa-miR-652-3p	3
1906	*EDN1*	Cardiovascular Diseases; Myocardial Ischemia (MI); Hypertensive disease; Atrial Fibrillation; Cardiac Arrhythmia; Inflammation; Asthma; Heart failure	8	hsa-miR-185-5p; hsa-miR-331-3p; hsa-let-7c-5p	3
112616	*CMTM7*	Heart failure	1	hsa-miR-331-3p; hsa-miR-185-5p; hsa-miR-218-5p	3
841	*CASP8*	Cerebral Hemorrhage	1	hsa-miR-143-3p; hsa-let-7c-5p; hsa-miR-106a-5p	3
7422	*VEGFA*	Cerebral Hemorrhage; Heart failure; Heart Diseases; Asthma; Inflammation; Myocardial Ischemia (MI)	6	hsa-miR-185-5p; hsa-miR-106a-5p; hsa-miR-331-3p	3
7168	*TPM1*	Hypertensive disease	1	hsa-miR-143-3p; hsa-miR-218-5p; hsa-miR-106a-5p	3
2634	*GBP2*	Myocardial Ischemia (MI)	1	hsa-miR-143-3p; hsa-miR-106a-5p; hsa-miR-185-5p	3
2778	*GNAS*	Hypertensive disease	1	hsa-miR-143-3p; hsa-miR-218-5p; hsa-miR-652-3p	3
581	*BAX*	Cerebral Hemorrhage; Ischemia; Myocardial	3	hsa-miR-185-5p; hsa-miR-331-3p; hsa-let-7c-5p	3

Entrez ID	Gene	CVDs (DisGeNET db v4.0)	CVD number	EV-miRNA targets	EV-miRNA number
		Infarction			
2150	*F2RL1*	Myocardial Ischemia (MI); Inflammation	2	hsa-miR-185-5p; hsa-miR-106a-5p; hsa-miR-143-3p	3
56996	*SLC12A9*	Hypertensive disease	1	hsa-let-7c-5p; hsa-miR-642a-5p; hsa-miR-331-3p	3
781	*CACNA2D1*	Hypertensive disease	1	hsa-miR-143-3p; hsa-let-7c-5p; hsa-miR-106a-5p	3
554	*AVPR2*	Heart failure	1	hsa-miR-185-5p; hsa-miR-331-3p; hsa-miR-642a-5p	3
4633	*MYL2*	Coronary heart disease	1	hsa-miR-99b-5p; hsa-miR-143-3p; hsa-miR-185-5p	3
1401	*CRP*	Inflammation; Cardiovascular Diseases; Myocardial Ischemia (MI); Heart failure; Thrombosis; Heart Diseases; Hypertensive disease	7	hsa-let-7c-5p; hsa-miR-143-3p; hsa-miR-218-5p	3
8204	*NRIP1*	Myocardial Infarction; Heart failure	2	hsa-miR-143-3p; hsa-miR-642a-5p; hsa-miR-106a-5p	3
8525	*DGKZ*	Myocardial Infarction	1	hsa-let-7c-5p; hsa-miR-642a-5p; hsa-miR-331-3p	3
55876	*GSDMB*	Asthma	1	hsa-let-7c-5p; hsa-miR-106a-5p; hsa-miR-652-3p	3
3115	*HLA-DPB1*	Asthma	1	hsa-miR-331-3p; hsa-miR-143-3p; hsa-miR-106a-5p	3
7292	*TNFSF4*	Myocardial Infarction	1	hsa-miR-143-3p; hsa-miR-185-5p; hsa-miR-106a-5p	3
9159	*PCSK7*	Cardiovascular Diseases	1	hsa-miR-143-3p; hsa-miR-331-3p; hsa-miR-185-5p	3
6559	*SLC12A3*	Hypertensive disease	1	hsa-miR-106a-5p; hsa-let-7c-5p; hsa-miR-143-3p	3
7357	*UGCG*	Myocardial Ischemia (MI)	1	hsa-let-7c-5p; hsa-miR-99b-5p; hsa-miR-106a-5p	3
7296	*TXNRD1*	Myocardial Ischemia (MI)	1	hsa-miR-143-3p; hsa-let-7c-5p; hsa-miR-106a-5p	3
1395	*CRHR2*	Inflammation; Hypertensive disease	2	hsa-miR-185-5p; hsa-miR-331-3p; hsa-miR-642a-5p	3
7804	*LRP8*	Myocardial Infarction	1	hsa-miR-185-5p; hsa-miR-106a-5p; hsa-miR-143-3p	3
6786	*STIM1*	Hypertensive disease	1	hsa-miR-331-3p; hsa-miR-185-5p; hsa-miR-106a-5p	3
412	*STS*	Inflammation	1	hsa-miR-185-5p; hsa-miR-143-3p; hsa-let-7c-5p	3
6097	*RORC*	Inflammation	1	hsa-miR-642a-5p; hsa-let-7c-5p; hsa-miR-106a-5p	3
5166	*PDK4*	Myocardial Ischemia (MI)	1	hsa-miR-106a-5p; hsa-miR-143-3p; hsa-miR-642a-5p	3
7099	*TLR4*	Inflammation	1	hsa-miR-642a-5p; hsa-let-7c-5p; hsa-miR-143-3p	3

Entrez ID	Gene	CVDs (DisGeNET db v4.0)	CVD number	EV-miRNA targets	EV-miRNA number
2155	*F7*	Cerebral Hemorrhage	1	hsa-miR-185-5p; hsa-miR-642a-5p; hsa-miR-331-3p	3
186	*AGTR2*	Hypertensive disease	1	hsa-miR-106a-5p; hsa-miR-143-3p; hsa-let-7c-5p	3
2247	*FGF2*	Inflammation; Myocardial Ischemia (MI)	2	hsa-miR-652-3p; hsa-miR-106a-5p; hsa-miR-642a-5p	3
8639	*AOC3*	Hypertensive disease	1	hsa-miR-185-5p; hsa-miR-642a-5p; hsa-miR-331-3p	3
5444	*PON1*	Cardiovascular Diseases; Coronary heart disease	2	hsa-miR-218-5p; hsa-miR-331-3p; hsa-let-7c-5p	3
4968	*OGG1*	Heart Diseases	1	hsa-miR-106a-5p; hsa-miR-185-5p; hsa-let-7c-5p	3
1000	*CDH2*	Myocardial Ischemia (MI)	1	hsa-miR-218-5p; hsa-miR-106a-5p; hsa-miR-642a-5p	3
948	*CD36*	Hypertensive disease; Coronary heart disease; Myocardial Ischemia (MI)	3	hsa-miR-143-3p; hsa-miR-106a-5p; hsa-miR-218-5p	3
29119	*CTNNA3*	Asthma	1	hsa-miR-143-3p; hsa-miR-642a-5p; hsa-miR-185-5p	3
1241	*LTB4R*	Inflammation	1	hsa-miR-642a-5p; hsa-miR-331-3p; hsa-miR-106a-5p	3
1634	*DCN*	Myocardial Ischemia (MI)	1	hsa-miR-106a-5p; hsa-miR-218-5p; hsa-let-7c-5p	3
3552	*IL1A*	Myocardial Ischemia (MI); Inflammation	2	hsa-miR-106a-5p; hsa-miR-218-5p; hsa-miR-642a-5p	3
1281	*COL3A1*	Hypertensive disease	1	hsa-let-7c-5p; hsa-miR-331-3p; hsa-miR-106a-5p	3
4313	*MMP2*	Inflammation; Myocardial Infarction; Cerebral Hemorrhage; Hypertensive disease	4	hsa-miR-143-3p; hsa-miR-185-5p; hsa-miR-106a-5p	3
2690	*GHR*	Myocardial Ischemia (MI)	1	hsa-miR-652-3p; hsa-miR-143-3p; hsa-let-7c-5p	3
156	*ADRBK1*	Hypertensive disease	1	hsa-miR-331-3p; hsa-miR-642a-5p; hsa-miR-218-5p	3
11243	*PMF1*	Cerebral Hemorrhage	1	hsa-miR-185-5p; hsa-miR-331-3p; hsa-let-7c-5p	3
3779	*KCNMB1*	Hypertensive disease	1	hsa-miR-652-3p; hsa-miR-218-5p; hsa-miR-331-3p	3
2158	*F9*	Thrombosis	1	hsa-miR-331-3p; hsa-miR-106a-5p; hsa-miR-185-5p	3
3146	*HMGB1*	Inflammation; Myocardial Ischemia (MI)	2	hsa-miR-106a-5p; hsa-miR-218-5p; hsa-miR-143-3p	3
84439	*HHIPL1*	Coronary heart disease	1	hsa-miR-185-5p; hsa-miR-331-3p; hsa-let-7c-5p	3

Entrez ID	Gene	CVDs (DisGeNET db v4.0)	CVD number	EV-miRNA targets	EV-miRNA number
6341	*SCO1*	Heart Diseases	1	hsa-miR-652-3p; hsa-miR-106a-5p; hsa-miR-185-5p	3
3158	*HMGCS2*	Myocardial Ischemia (MI)	1	hsa-miR-185-5p; hsa-miR-143-3p; hsa-miR-331-3p	3
2162	*F13A1*	Myocardial Infarction	1	hsa-miR-143-3p; hsa-miR-218-5p; hsa-miR-185-5p	3
23293	*SMG6*	Coronary heart disease	1	hsa-let-7c-5p; hsa-miR-331-3p; hsa-miR-185-5p	3
4254	*KITLG*	Myocardial Ischemia (MI)	1	hsa-miR-185-5p; hsa-miR-143-3p; hsa-miR-642a-5p	3
8553	*BHLHE40*	Myocardial Ischemia (MI)	1	hsa-miR-106a-5p; hsa-miR-642a-5p; hsa-let-7c-5p	3
4881	*NPR1*	Heart failure; Hypertensive disease	2	hsa-miR-218-5p; hsa-miR-185-5p; hsa-miR-331-3p	3
196	*AHR*	Inflammation; Hypertensive disease	2	hsa-miR-143-3p; hsa-let-7c-5p; hsa-miR-185-5p	3
821	*CANX*	Myocardial Ischemia (MI)	1	hsa-miR-642a-5p; hsa-miR-185-5p; hsa-miR-106a-5p	3
624	*BDKRB2*	Hypertensive disease; Thrombosis; Inflammation	3	hsa-miR-185-5p; hsa-miR-218-5p; hsa-miR-642a-5p	3
5209	*PFKFB3*	Myocardial Ischemia (MI)	1	hsa-miR-642a-5p; hsa-miR-106a-5p; hsa-miR-185-5p	3
858	*CAV2*	Atrial Fibrillation	1	hsa-miR-106a-5p; hsa-miR-218-5p; hsa-miR-143-3p	3
9451	*EIF2AK3*	Myocardial Ischemia (MI)	1	hsa-miR-143-3p; hsa-miR-106a-5p; hsa-miR-218-5p	3
143872	*ARHGAP42*	Hypertensive disease	1	hsa-miR-218-5p; hsa-miR-652-3p; hsa-miR-185-5p	3
11221	*DUSP10*	Inflammation	1	hsa-miR-652-3p; hsa-miR-185-5p; hsa-miR-106a-5p	3
3030	*HADHA*	Myocardial Ischemia (MI)	1	hsa-miR-143-3p; hsa-miR-331-3p; hsa-miR-185-5p	3
30009	*TBX21*	Asthma; Inflammation	2	hsa-miR-106a-5p; hsa-miR-143-3p; hsa-miR-642a-5p	3
83992	*CTTNBP2*	Myocardial Ischemia (MI)	1	hsa-miR-185-5p; hsa-miR-218-5p; hsa-miR-331-3p	3
51330	*TNFRSF12A*	Myocardial Infarction	1	hsa-let-7c-5p; hsa-miR-642a-5p; hsa-miR-331-3p	3
8613	*PPAP2B*	Coronary heart disease	1	hsa-miR-185-5p; hsa-miR-652-3p; hsa-miR-218-5p	3
65125	*WNK1*	Hypertensive disease	1	hsa-miR-652-3p; hsa-miR-185-5p; hsa-miR-106a-5p	3
2588	*GALNS*	Inflammation	1	hsa-miR-642a-5p; hsa-miR-331-3p; hsa-miR-106a-5p	3
64116	*SLC39A8*	Hypertensive disease	1	hsa-miR-106a-5p; hsa-miR-218-5p; hsa-miR-331-3p	3
57104	*PNPLA2*	Heart Diseases	1	hsa-miR-218-5p; hsa-miR-185-5p	2
284	*ANGPT1*	Inflammation	1	hsa-miR-185-5p; hsa-miR-331-3p	2
3717	*JAK2*	Myocardial Ischemia (MI)	1	hsa-miR-106a-5p; hsa-miR-185-5p	2

Entrez ID	Gene	CVDs (DisGeNET db v4.0)	CVD number	EV-miRNA targets	EV-miRNA number
2149	*F2R*	Inflammation	1	hsa-miR-106a-5p; hsa-miR-143-3p	2
117	*ADCYAP1R1*	Asthma	1	hsa-miR-143-3p; hsa-miR-642a-5p	2
3084	*NRG1*	Heart failure	1	hsa-miR-143-3p; hsa-let-7c-5p	2
3827	*KNG1*	Cardiac Arrhythmia	1	hsa-miR-185-5p; hsa-miR-143-3p	2
3306	*HSPA2*	Myocardial Ischemia (MI)	1	hsa-miR-106a-5p; hsa-miR-185-5p	2
84876	*ORAI1*	Hypertensive disease	1	hsa-miR-642a-5p; hsa-miR-106a-5p	2
5144	*PDE4D*	Asthma	1	hsa-miR-185-5p; hsa-miR-642a-5p	2
7301	*TYRO3*	Thrombosis	1	hsa-let-7c-5p; hsa-miR-642a-5p	2
7498	*XDH*	Heart failure; Heart Diseases; Ischemia	3	hsa-miR-185-5p; hsa-miR-106a-5p	2
916	*CD3E*	Inflammation	1	hsa-miR-143-3p; hsa-miR-185-5p	2
4478	*MSN*	Myocardial Ischemia (MI)	1	hsa-miR-185-5p; hsa-let-7c-5p	2
3082	*HGF*	Thrombosis; Myocardial Ischemia (MI)	2	hsa-let-7c-5p; hsa-miR-185-5p	2
5734	*PTGER4*	Inflammation	1	hsa-miR-185-5p; hsa-miR-106a-5p	2
23294	*ANKS1A*	Coronary heart disease	1	hsa-miR-106a-5p; hsa-miR-331-3p	2
50507	*NOX4*	Heart failure	1	hsa-miR-106a-5p; hsa-miR-99b-5p	2
1958	*EGR1*	Inflammation; Ischemia	2	hsa-miR-642a-5p; hsa-miR-143-3p	2
4049	*LTA*	Myocardial Infarction	1	hsa-miR-185-5p; hsa-miR-331-3p	2
9373	*PLAA*	Inflammation	1	hsa-miR-185-5p; hsa-miR-642a-5p	2
5028	*P2RY1*	Thrombosis	1	hsa-miR-106a-5p; hsa-miR-185-5p	2
2475	*MTOR*	Hypertensive disease	1	hsa-let-7c-5p; hsa-miR-99b-5p	2
2678	*GGT1*	Myocardial Infarction	1	hsa-miR-218-5p; hsa-miR-642a-5p	2
10516	*FBLN5*	Heart failure	1	hsa-let-7c-5p; hsa-miR-185-5p	2
1431	*CS*	Heart failure	1	hsa-miR-185-5p; hsa-let-7c-5p	2
3097	*HIVEP2*	Myocardial Ischemia (MI)	1	hsa-miR-218-5p; hsa-miR-642a-5p	2
3313	*HSPA9*	Myocardial Ischemia (MI)	1	hsa-miR-642a-5p; hsa-let-7c-5p	2
140	*ADORA3*	Ischemia; Myocardial Infarction	2	hsa-miR-642a-5p; hsa-miR-185-5p	2

Entrez ID	Gene	CVDs (DisGeNET db v4.0)	CVD number	EV-miRNA targets	EV-miRNA number
623	*BDKRB1*	Inflammation	1	hsa-miR-185-5p; hsa-miR-642a-5p	2
6272	*SORT1*	Cardiovascular Diseases	1	hsa-miR-185-5p; hsa-miR-106a-5p	2
3119	*HLA-DQB1*	Asthma	1	hsa-miR-218-5p; hsa-miR-143-3p	2
3117	*HLA-DQA1*	Asthma	1	hsa-let-7c-5p; hsa-miR-185-5p	2
2243	*FGA*	Thrombosis	1	hsa-miR-143-3p; hsa-miR-185-5p	2
1636	*ACE*	Cerebral Hemorrhage; Heart failure; Cardiac Arrhythmia; Myocardial Infarction; Coronary heart disease; Hypertensive disease; Atrial Fibrillation; Cardiovascular Diseases	8	hsa-miR-331-3p; hsa-miR-185-5p	2
2730	*GCLM*	Myocardial Infarction	1	hsa-miR-143-3p; hsa-miR-106a-5p	2
161725	*OTUD7A*	Heart failure	1	hsa-miR-652-3p; hsa-miR-143-3p	2
2152	*F3*	Myocardial Ischemia (MI); Thrombosis	2	hsa-let-7c-5p; hsa-miR-106a-5p	2
203	*AK1*	Myocardial Ischemia (MI)	1	hsa-miR-143-3p; hsa-miR-106a-5p	2
54805	*CNNM2*	Cardiac Arrhythmia; Coronary heart disease	2	hsa-miR-106a-5p; hsa-miR-642a-5p	2
5328	*PLAU*	Myocardial Infarction; Coronary heart disease; Asthma; Ischemia; Thrombosis; Cerebral Hemorrhage	6	hsa-miR-143-3p; hsa-miR-106a-5p	2
6262	*RYR2*	Myocardial Ischemia (MI)	1	hsa-let-7c-5p; hsa-miR-185-5p	2
3627	*CXCL10*	Myocardial Ischemia (MI)	1	hsa-miR-185-5p; hsa-let-7c-5p	2
3741	*KCNA5*	Atrial Fibrillation	1	hsa-miR-185-5p; hsa-miR-642a-5p	2
3688	*ITGB1*	Heart failure	1	hsa-miR-642a-5p; hsa-miR-106a-5p	2
85480	*TSLP*	Asthma	1	hsa-miR-106a-5p; hsa-miR-143-3p	2
1052	*CEBPD*	Myocardial Ischemia (MI)	1	hsa-miR-106a-5p; hsa-let-7c-5p	2

Entrez ID	Gene	CVDs (DisGeNET db v4.0)	CVD number	EV-miRNA targets	EV-miRNA number
9547	*CXCL14*	Asthma	1	hsa-miR-106a-5p; hsa-miR-185-5p	2
2710	*GK*	Myocardial Ischemia (MI)	1	hsa-miR-143-3p; hsa-miR-106a-5p	2
3569	*IL6*	Heart failure; Myocardial Infarction; Myocardial Ischemia (MI); Ischemia; Inflammation	5	hsa-let-7c-5p; hsa-miR-652-3p	2
5580	*PRKCD*	Hypertensive disease	1	hsa-miR-642a-5p; hsa-miR-218-5p	2
8882	*ZPR1*	Coronary heart disease	1	hsa-miR-331-3p; hsa-miR-106a-5p	2
5624	*PROC*	Thrombosis; Hypertensive disease	2	hsa-miR-218-5p; hsa-miR-642a-5p	2
9637	*FEZ2*	Myocardial Ischemia (MI)	1	hsa-miR-652-3p; hsa-miR-106a-5p	2
5786	*PTPRA*	Myocardial Ischemia (MI)	1	hsa-miR-218-5p; hsa-miR-185-5p	2
6794	*STK11*	Hypertensive disease	1	hsa-miR-331-3p; hsa-miR-106a-5p	2
4343	*MOV10*	Hypertensive disease	1	hsa-miR-652-3p; hsa-miR-642a-5p	2
590	*BCHE*	Cardiovascular Diseases	1	hsa-miR-185-5p; hsa-miR-218-5p	2
9943	*OXSR1*	Hypertensive disease	1	hsa-miR-218-5p; hsa-miR-185-5p	2
5970	*RELA*	Hypertensive disease	1	hsa-miR-185-5p; hsa-miR-331-3p	2
3596	*IL13*	Asthma; Inflammation	2	hsa-miR-642a-5p; hsa-let-7c-5p	2
7124	*TNF*	Inflammation; Asthma; Myocardial Infarction; Myocardial Ischemia (MI); Heart failure; Thrombosis; Hypertensive disease	7	hsa-miR-331-3p; hsa-miR-185-5p	2
3458	*IFNG*	Heart failure; Inflammation	2	hsa-miR-331-3p; hsa-miR-143-3p	2
389	*RHOC*	Myocardial Ischemia (MI)	1	hsa-miR-106a-5p; hsa-miR-185-5p	2
53345	*TM6SF2*	Myocardial Infarction	1	hsa-miR-642a-5p; hsa-miR-143-3p	2
1674	*DES*	Atrial Fibrillation	1	hsa-miR-331-3p; hsa-miR-642a-5p	2
10461	*MERTK*	Thrombosis	1	hsa-let-7c-5p; hsa-miR-106a-5p	2
811	*CALR*	Myocardial Ischemia (MI)	1	hsa-miR-185-5p; hsa-miR-143-3p	2
55829	*VIMP*	Inflammation	1	hsa-miR-642a-5p; hsa-miR-106a-5p	2

Entrez ID	Gene	CVDs (DisGeNET db v4.0)	CVD number	EV-miRNA targets	EV-miRNA number
11255	*HRH3*	Hypertensive disease	1	hsa-miR-642a-5p; hsa-miR-331-3p	2
5627	*PROS1*	Thrombosis	1	hsa-miR-143-3p; hsa-miR-642a-5p	2
5468	*PPARG*	Hypertensive disease; Ischemia; Inflammation	3	hsa-miR-642a-5p; hsa-miR-185-5p	2
149628	*PYHIN1*	Asthma	1	hsa-miR-106a-5p; hsa-miR-218-5p	2
834	*CASP1*	Inflammation	1	hsa-miR-218-5p; hsa-miR-185-5p	2
2740	*GLP1R*	Hypertensive disease	1	hsa-miR-185-5p; hsa-miR-143-3p	2
201475	*RAB12*	Myocardial Ischemia (MI)	1	hsa-miR-185-5p; hsa-miR-106a-5p	2
1524	*CX3CR1*	Coronary heart disease	1	hsa-miR-185-5p; hsa-miR-642a-5p	2
8315	*BRAP*	Myocardial Infarction	1	hsa-miR-143-3p; hsa-miR-642a-5p	2
213	*ALB*	Hypertensive disease; Heart Diseases; Heart failure; Cardiovascular Diseases	4	hsa-miR-143-3p; hsa-miR-185-5p	2
7097	*TLR2*	Inflammation	1	hsa-miR-218-5p; hsa-miR-143-3p	2
1153	*CIRBP*	Myocardial Ischemia (MI)	1	hsa-miR-106a-5p; hsa-miR-642a-5p	2
4986	*OPRK1*	Cardiac Arrhythmia	1	hsa-miR-652-3p; hsa-miR-143-3p	2
23418	*CRB1*	Asthma	1	hsa-miR-185-5p; hsa-let-7c-5p	2
9314	*KLF4*	Myocardial Ischemia (MI)	1	hsa-miR-642a-5p; hsa-miR-218-5p	2
146862	*UNC45B*	Heart Diseases	1	hsa-miR-185-5p; hsa-miR-143-3p	2
1843	*DUSP1*	Myocardial Ischemia (MI)	1	hsa-let-7c-5p; hsa-miR-218-5p	2
1390	*CREM*	Myocardial Infarction	1	hsa-let-7c-5p; hsa-miR-106a-5p	2
2322	*FLT3*	Thrombosis	1	hsa-miR-331-3p; hsa-miR-106a-5p	2
8773	*SNAP23*	Myocardial Ischemia (MI)	1	hsa-let-7c-5p; hsa-miR-106a-5p	2
90226	*UCN2*	Heart failure	1	hsa-miR-185-5p; hsa-let-7c-5p	2
5683	*PSMA2*	Heart Diseases	1	hsa-miR-642a-5p; hsa-miR-106a-5p	2
9536	*PTGES*	Inflammation	1	hsa-miR-185-5p; hsa-miR-331-3p	2
6401	*SELE*	Atrial Fibrillation; Myocardial Ischemia (MI); Cardiovascular Diseases	3	hsa-miR-185-5p; hsa-miR-642a-5p	2

Entrez ID	Gene	CVDs (DisGeNET db v4.0)	CVD number	EV-miRNA targets	EV-miRNA number
169026	*SLC30A8*	Asthma	1	hsa-miR-143-3p; hsa-miR-185-5p	2
207	*AKT1*	Inflammation	1	hsa-miR-185-5p; hsa-miR-143-3p	2
10111	*RAD50*	Asthma	1	hsa-miR-652-3p; hsa-miR-185-5p	2
51196	*PLCE1*	Hypertensive disease	1	hsa-miR-106a-5p; hsa-miR-331-3p	2
5340	*PLG*	Hypertensive disease	1	hsa-miR-331-3p; hsa-let-7c-5p	2
23411	*SIRT1*	Heart Diseases	1	hsa-miR-106a-5p; hsa-miR-143-3p	2
140733	*MACROD2*	Hypertensive disease	1	hsa-miR-642a-5p; hsa-miR-106a-5p	2
5236	*PGM1*	Myocardial Ischemia (MI)	1	hsa-miR-652-3p; hsa-miR-642a-5p	2
51088	*KLHL5*	Asthma	1	hsa-miR-106a-5p; hsa-miR-218-5p	2
3605	*IL17A*	Inflammation	1	hsa-miR-185-5p; hsa-miR-106a-5p	2
3291	*HSD11B2*	Hypertensive disease; Asthma; Myocardial Infarction	3	hsa-miR-218-5p; hsa-miR-185-5p	2
9294	*S1PR2*	Heart Diseases	1	hsa-miR-331-3p; hsa-miR-106a-5p	2
55219	*TMEM57*	Coronary heart disease	1	hsa-miR-106a-5p; hsa-miR-331-3p	2
6584	*SLC22A5*	Inflammation; Asthma	2	hsa-miR-218-5p; hsa-miR-106a-5p	2
2626	*GATA4*	Myocardial Infarction	1	hsa-miR-185-5p; hsa-miR-331-3p	2
4803	*NGF*	Inflammation	1	hsa-miR-185-5p; hsa-let-7c-5p	2
5327	*PLAT*	Heart failure; Cerebral Hemorrhage; Thrombosis; Hypertensive disease; Myocardial Infarction	5	hsa-miR-185-5p; hsa-miR-642a-5p	2
7538	*ZFP36*	Myocardial Ischemia (MI); Inflammation	2	hsa-miR-106a-5p; hsa-miR-185-5p	2
4314	*MMP3*	Cerebral Hemorrhage	1	hsa-miR-185-5p; hsa-miR-106a-5p	2
2056	*EPO*	Inflammation; Myocardial Infarction; Heart Diseases; Thrombosis; Hypertensive disease	5	hsa-miR-331-3p; hsa-miR-143-3p	2
221692	*PHACTR1*	Myocardial Infarction;	2	hsa-miR-185-5p; hsa-miR-143-3p	2

Entrez ID	Gene	CVDs (DisGeNET db v4.0)	CVD number	EV-miRNA targets	EV-miRNA number
		Coronary heart disease			
6402	*SELL*	Myocardial Infarction	1	hsa-miR-185-5p; hsa-miR-143-3p	2
1398	*CRK*	Myocardial Ischemia (MI)	1	hsa-miR-106a-5p; hsa-let-7c-5p	2
5879	*RAC1*	Heart failure	1	hsa-miR-99b-5p; hsa-miR-652-3p	2
154	*ADRB2*	Asthma; Myocardial Ischemia (MI)	2	hsa-let-7c-5p; hsa-miR-218-5p	2
1356	*CP*	Ischemia	1	hsa-let-7c-5p; hsa-miR-642a-5p	2
5329	*PLAUR*	Inflammation	1	hsa-miR-143-3p; hsa-miR-106a-5p	2
472	*ATM*	Myocardial Infarction	1	hsa-miR-143-3p; hsa-miR-106a-5p	2
118	*ADD1*	Hypertensive disease	1	hsa-miR-106a-5p; hsa-miR-143-3p	2
10747	*MASP2*	Inflammation	1	hsa-miR-185-5p; hsa-miR-143-3p	2
6935	*ZEB1*	Myocardial Ischemia (MI)	1	hsa-miR-143-3p; hsa-let-7c-5p	2
387129	*NPSR1*	Asthma	1	hsa-miR-185-5p; hsa-miR-218-5p	2
1437	*CSF2*	Inflammation; Heart failure	2	hsa-miR-642a-5p	1
7412	*VCAM1*	Myocardial Ischemia (MI); Cardiovascular Diseases; Hypertensive disease	3	hsa-let-7c-5p	1
3553	*IL1B*	Heart failure; Inflammation; Asthma; Myocardial Ischemia (MI); Myocardial Infarction	5	hsa-miR-185-5p	1
968	*CD68*	Ischemia	1	hsa-miR-106a-5p	1
7917	*BAG6*	Hypertensive disease	1	hsa-miR-331-3p	1
2660	*MSTN*	Heart failure	1	hsa-miR-143-3p	1
90865	*IL33*	Asthma	1	hsa-miR-331-3p	1
5445	*PON2*	Cardiovascular Diseases	1	hsa-miR-106a-5p	1
3674	*ITGA2B*	Cerebral Hemorrhage	1	hsa-miR-99b-5p	1
5687	*PSMA6*	Myocardial Infarction	1	hsa-miR-185-5p	1
2171	*FABP5*	Myocardial Ischemia (MI)	1	hsa-miR-185-5p	1
4353	*MPO*	Cardiovascular Diseases; Inflammation	2	hsa-miR-218-5p	1

Entrez ID	Gene	CVDs (DisGeNET db v4.0)	CVD number	EV-miRNA targets	EV-miRNA number
54738	*FEV*	Myocardial Ischemia (MI)	1	hsa-miR-106a-5p	1
6356	*CCL11*	Asthma; Inflammation	2	hsa-miR-185-5p	1
152	*ADRA2C*	Heart failure	1	hsa-miR-331-3p	1
10665	*C6orf10*	Coronary heart disease; Asthma	2	hsa-let-7c-5p	1
2160	*F11*	Hypertensive disease	1	hsa-miR-331-3p	1
3397	*ID1*	Myocardial Ischemia (MI)	1	hsa-miR-331-3p	1
7076	*TIMP1*	Heart Diseases; Inflammation	2	hsa-miR-185-5p	1
2729	*GCLC*	Myocardial Ischemia (MI); Myocardial Infarction; Hypertensive disease	3	hsa-miR-106a-5p	1
3357	*HTR2B*	Heart failure; Hypertensive disease	2	hsa-miR-143-3p	1
134	*ADORA1*	Myocardial Infarction; Ischemia	2	hsa-miR-331-3p	1
4855	*NOTCH4*	Asthma	1	hsa-miR-185-5p	1
10059	*DNM1L*	Myocardial Ischemia (MI)	1	hsa-miR-106a-5p	1
114548	*NLRP3*	Inflammation	1	hsa-miR-106a-5p	1
210	*ALAD*	Hypertensive disease	1	hsa-miR-642a-5p	1
486	*FXYD2*	Hypertensive disease	1	hsa-miR-218-5p	1
567	*B2M*	Heart Diseases	1	hsa-miR-106a-5p	1
5741	*PTH*	Heart failure; Hypertensive disease	2	hsa-miR-106a-5p	1
6258	*RXRG*	Myocardial Ischemia (MI)	1	hsa-miR-106a-5p	1
3593	*IL12B*	Asthma	1	hsa-miR-652-3p	1
142	*PARP1*	Asthma; Inflammation	2	hsa-miR-106a-5p	1
1244	*ABCC2*	Heart Diseases	1	hsa-let-7c-5p	1
3726	*JUNB*	Myocardial Ischemia (MI)	1	hsa-miR-331-3p	1
4329	*ALDH6A1*	Myocardial Ischemia (MI)	1	hsa-let-7c-5p	1
783	*CACNB2*	Hypertensive disease	1	hsa-let-7c-5p	1

Entrez ID	Gene	CVDs (DisGeNET db v4.0)	CVD number	EV-miRNA targets	EV-miRNA number
54982	*CLN6*	Inflammation	1	hsa-miR-143-3p	1
3399	*ID3*	Myocardial Ischemia (MI)	1	hsa-miR-185-5p	1
2863	*GPR39*	Hypertensive disease	1	hsa-miR-143-3p	1
7478	*WNT8A*	Atrial Fibrillation	1	hsa-miR-642a-5p	1
2646	*GCKR*	Cardiovascular Diseases	1	hsa-miR-185-5p	1
6648	*SOD2*	Hypertensive disease; Heart failure; Myocardial Infarction; Myocardial Ischemia (MI); Ischemia	5	hsa-miR-106a-5p	1
2701	*GJA4*	Hypertensive disease	1	hsa-miR-185-5p	1
383	*ARG1*	Asthma	1	hsa-miR-218-5p	1
847	*CAT*	Hypertensive disease; Heart failure; Myocardial Infarction; Asthma	4	hsa-miR-331-3p	1
4689	*NCF4*	Heart Diseases	1	hsa-miR-642a-5p	1
5997	*RGS2*	Hypertensive disease	1	hsa-miR-106a-5p	1
462	*SERPINC1*	Cerebral Hemorrhage; Thrombosis	2	hsa-miR-143-3p	1
10965	*ACOT2*	Myocardial Ischemia (MI)	1	hsa-miR-185-5p	1
6275	*S100A4*	Ischemia	1	hsa-miR-106a-5p	1
2100	*ESR2*	Atrial Fibrillation; Cardiac Arrhythmia; Coronary heart disease	3	hsa-let-7c-5p	1
7351	*UCP2*	Hypertensive disease	1	hsa-miR-185-5p	1
25890	*ABI3BP*	Asthma	1	hsa-miR-218-5p	1
3758	*KCNJ1*	Hypertensive disease	1	hsa-miR-185-5p	1
3567	*IL5*	Asthma	1	hsa-miR-642a-5p	1
6649	*SOD3*	Heart failure; Hypertensive disease	2	hsa-miR-185-5p	1

Entrez ID	Gene	CVDs (DisGeNET db v4.0)	CVD number	EV-miRNA targets	EV-miRNA number
2920	*CXCL2*	Myocardial Ischemia (MI); Inflammation; Hypertensive disease; Heart failure	4	hsa-miR-185-5p	1
4319	*MMP10*	Asthma	1	hsa-miR-99b-5p	1
406	*ARNTL*	Myocardial Ischemia (MI)	1	hsa-miR-143-3p	1
23228	*PLCL2*	Myocardial Infarction	1	hsa-miR-218-5p	1
1577	*CYP3A5*	Thrombosis	1	hsa-miR-185-5p	1
27035	*NOX1*	Heart failure	1	hsa-miR-106a-5p	1
2006	*ELN*	Hypertensive disease; Myocardial Ischemia (MI)	2	hsa-miR-331-3p	1
240	*ALOX5*	Asthma	1	hsa-miR-143-3p	1
8678	*BECN1*	Myocardial Infarction	1	hsa-miR-106a-5p	1
871	*SERPINH1*	Ischemia	1	hsa-miR-642a-5p	1
217	*ALDH2*	Coronary heart disease; Asthma	2	hsa-let-7c-5p	1
2946	*GSTM2*	Myocardial Ischemia (MI)	1	hsa-miR-185-5p	1
100527963	*PMF1-BGLAP*	Cerebral Hemorrhage	1	hsa-miR-185-5p	1
3098	*HK1*	Myocardial Ischemia (MI)	1	hsa-miR-185-5p	1
2953	*GSTT2*	Hypertensive disease	1	hsa-miR-185-5p	1
3757	*KCNH2*	Cardiac Arrhythmia	1	hsa-miR-218-5p	1
4987	*OPRL1*	Cardiac Arrhythmia	1	hsa-let-7c-5p	1
1813	*DRD2*	Hypertensive disease	1	hsa-miR-185-5p	1
1270	*CNTF*	Cerebral Hemorrhage	1	hsa-let-7c-5p	1
3032	*HADHB*	Heart Diseases	1	hsa-miR-652-3p	1
1158	*CKM*	Myocardial Infarction; Ischemia	2	hsa-miR-185-5p	1
10452	*TOMM40*	Cardiovascular Diseases	1	hsa-miR-331-3p	1
6352	*CCL5*	Asthma	1	hsa-miR-106a-5p	1
2876	*GPX1*	Cardiovascular Diseases; Hypertensive disease	2	hsa-miR-185-5p	1

Entrez ID	Gene	CVDs (DisGeNET db v4.0)	CVD number	EV-miRNA targets	EV-miRNA number
3620	*IDO1*	Inflammation	1	hsa-miR-106a-5p	1
9445	*ITM2B*	Hypertensive disease	1	hsa-miR-143-3p	1
3113	*HLA-DPA1*	Asthma	1	hsa-let-7c-5p	1
2950	*GSTP1*	Asthma; Myocardial Ischemia (MI)	2	hsa-miR-185-5p	1
64240	*ABCG5*	Coronary heart disease	1	hsa-miR-143-3p	1
91851	*CHRDL1*	Coronary heart disease	1	hsa-miR-185-5p	1
2353	*FOS*	Hypertensive disease	1	hsa-let-7c-5p	1
2212	*FCGR2A*	Thrombosis	1	hsa-miR-185-5p	1
5034	*P4HB*	Myocardial Ischemia (MI)	1	hsa-miR-185-5p	1
136991	*ASZ1*	Coronary heart disease	1	hsa-miR-218-5p	1
2159	*F10*	Thrombosis	1	hsa-miR-331-3p	1
2053	*EPHX2*	Heart failure	1	hsa-miR-185-5p	1
338	*APOB*	Cardiovascular Diseases; Coronary heart disease	2	hsa-miR-218-5p	1
25902	*MTHFD1L*	Coronary heart disease	1	hsa-miR-106a-5p	1
3459	*IFNGR1*	Myocardial Ischemia (MI)	1	hsa-miR-218-5p	1
7369	*UMOD*	Hypertensive disease	1	hsa-miR-143-3p	1
6817	*SULT1A1*	Myocardial Ischemia (MI)	1	hsa-miR-642a-5p	1
3065	*HDAC1*	Heart Diseases	1	hsa-miR-185-5p	1
478	*ATP1A3*	Heart failure	1	hsa-miR-185-5p	1
3091	*HIF1A*	Heart failure; Hypertensive disease	2	hsa-miR-106a-5p	1
6285	*S100B*	Cerebral Hemorrhage; Myocardial Infarction	2	hsa-miR-642a-5p	1
5037	*PEBP1*	Myocardial Ischemia (MI)	1	hsa-miR-143-3p	1
7222	*TRPC3*	Hypertensive disease	1	hsa-miR-218-5p	1
5515	*PPP2CA*	Asthma; Myocardial Ischemia (MI); Heart Diseases	3	hsa-miR-106a-5p	1
4982	*TNFRSF11B*	Inflammation	1	hsa-miR-143-3p	1

Entrez ID	Gene	CVDs (DisGeNET db v4.0)	CVD number	EV-miRNA targets	EV-miRNA number
3880	*KRT19*	Asthma	1	hsa-miR-642a-5p	1
6338	*SCNN1B*	Hypertensive disease	1	hsa-miR-185-5p	1
857	*CAV1*	Atrial Fibrillation; Hypertensive disease	2	hsa-miR-106a-5p	1
284110	*GSDMA*	Asthma	1	hsa-let-7c-5p	1
7018	*TF*	Inflammation	1	hsa-let-7c-5p	1
80724	*ACAD10*	Coronary heart disease	1	hsa-let-7c-5p	1
119032	*C10orf32*	Hypertensive disease	1	hsa-miR-106a-5p	1
952	*CD38*	Myocardial Ischemia (MI)	1	hsa-miR-106a-5p	1
7356	*SCGB1A1*	Inflammation; Asthma	2	hsa-miR-143-3p	1
166	*AES*	Myocardial Ischemia (MI)	1	hsa-miR-218-5p	1
6348	*CCL3*	Inflammation; Myocardial Ischemia (MI)	2	hsa-let-7c-5p	1
10544	*PROCR*	Inflammation	1	hsa-miR-106a-5p	1
1017	*CDK2*	Asthma	1	hsa-miR-642a-5p	1
1848	*DUSP6*	Myocardial Ischemia (MI)	1	hsa-miR-106a-5p	1
4589	*MUC7*	Asthma	1	hsa-miR-185-5p	1
2335	*FN1*	Hypertensive disease	1	hsa-miR-143-3p	1
9588	*PRDX6*	Myocardial Ischemia (MI)	1	hsa-miR-652-3p	1
177	*AGER*	Inflammation	1	hsa-miR-185-5p	1
11173	*ADAMTS7*	Coronary heart disease	1	hsa-let-7c-5p	1
2242	*FES*	Hypertensive disease	1	hsa-let-7c-5p	1
287	*ANK2*	Cardiac Arrhythmia	1	hsa-miR-106a-5p	1
60	*ACTB*	Myocardial Ischemia (MI)	1	hsa-miR-331-3p	1
50805	*IRX4*	Cardiovascular Diseases	1	hsa-miR-642a-5p	1
384	*ARG2*	Asthma	1	hsa-let-7c-5p	1
7039	*TGFA*	Inflammation	1	hsa-miR-652-3p	1
6863	*TAC1*	Inflammation	1	hsa-miR-218-5p	1
3135	*HLA-G*	Asthma	1	hsa-miR-143-3p	1

Entrez ID	Gene	CVDs (DisGeNET db v4.0)	CVD number	EV-miRNA targets	EV-miRNA number
84722	*PSRC1*	Coronary heart disease	1	hsa-miR-642a-5p	1
4057	*LTF*	Hypertensive disease; Inflammation	2	hsa-miR-185-5p	1
5350	*PLN*	Myocardial Infarction	1	hsa-miR-185-5p	1
3383	*ICAM1*	Hypertensive disease; Inflammation; Asthma; Myocardial Infarction; Myocardial Ischemia (MI); Cardiovascular Diseases	6	hsa-miR-642a-5p	1
6597	*SMARCA4*	Myocardial Infarction; Coronary heart disease	2	hsa-miR-99b-5p	1
632	*BGLAP*	Asthma	1	hsa-miR-185-5p	1
4879	*NPPB*	Inflammation; Cardiovascular Diseases; Heart failure; Cerebral Hemorrhage; Atrial Fibrillation; Heart Diseases; Hypertensive disease	7	hsa-miR-218-5p	1
7442	*TRPV1*	Inflammation	1	hsa-miR-642a-5p	1
7137	*TNNI3*	Heart Diseases; Myocardial Infarction	2	hsa-miR-185-5p	1
23224	*SYNE2*	Atrial Fibrillation	1	hsa-miR-106a-5p	1
8638	*OASL*	Cardiovascular Diseases	1	hsa-miR-331-3p	1
1786	*DNMT1*	Asthma	1	hsa-miR-185-5p	1
1280	*COL2A1*	Inflammation	1	hsa-miR-106a-5p	1
6701	*SPRR2B*	Asthma	1	hsa-miR-106a-5p	1
4878	*NPPA*	Heart failure; Hypertensive disease; Myocardial Ischemia (MI); Myocardial Infarction	4	hsa-miR-143-3p	1
5660	*PSAP*	Asthma	1		
246	*ALOX15*	Hypertensive disease	1		

Entrez ID	Gene	CVDs (DisGeNET db v4.0)	CVD number	EV-miRNA targets	EV-miRNA number
5020	*OXT*	Ischemia; Inflammation; Hypertensive disease	3		
644165	*BCRP3*	Thrombosis	1		
522	*ATP5J*	Heart Diseases	1		
6252	*RTN1*	Myocardial Ischemia (MI)	1		
51083	*GAL*	Hypertensive disease; Inflammation	2		
6876	*TAGLN*	Cardiovascular Diseases	1		
2944	*GSTM1*	Asthma	1		
4691	*NCL*	Myocardial Ischemia (MI)	1		
1071	*CETP*	Hypertensive disease; Cardiovascular Diseases	2		
653361	*NCF1*	Hypertensive disease	1		
4502	*MT2A*	Heart Diseases	1		
7139	*TNNT2*	Heart failure; Heart Diseases; Cardiovascular Diseases; Myocardial Ischemia (MI)	4		
1482	*NKX2-5*	Atrial Fibrillation	1		
4190	*MDH1*	Hypertensive disease	1		
1535	*CYBA*	Hypertensive disease; Heart Diseases	2		
6508	*SLC4A3*	Heart Diseases	1		
388165	*UBE2Q2P1*	Coronary heart disease	1		
5880	*RAC2*	Heart Diseases	1		
4223	*MEOX2*	Myocardial Ischemia (MI)	1		
226	*ALDOA*	Myocardial Ischemia (MI)	1		
102724766	*LOC102724766*	Cardiovascular Diseases	1		
1908	*EDN3*	Hypertensive disease	1		
10462	*CLEC10A*	Myocardial Ischemia (MI)	1		
3972	*LHB*	Hypertensive disease	1		

Entrez ID	Gene	CVDs (DisGeNET db v4.0)	CVD number	EV-miRNA targets	EV-miRNA number
407040	*MIR34A*	Heart Diseases; Inflammation	2		
114131	*UCN3*	Inflammation	1		
551	*AVP*	Cardiac Arrhythmia; Ischemia; Hypertensive disease; Heart failure	4		
51530	*ZC3HC1*	Coronary heart disease	1		
7054	*TH*	Heart Diseases	1		
5320	*PLA2G2A*	Myocardial Ischemia (MI)	1		
6403	*SELP*	Myocardial Ischemia (MI); Hypertensive disease; Thrombosis	3		
374	*AREG*	Asthma; Myocardial Ischemia (MI)	2		
60674	*GAS5*	Myocardial Ischemia (MI)	1		
2938	*GSTA1*	Myocardial Ischemia (MI)	1		
221357	*GSTA5*	Hypertensive disease	1		
2625	*GATA3*	Inflammation	1		
5111	*PCNA*	Ischemia	1		
2879	*GPX4*	Heart failure	1		
487	*ATP2A1*	Heart failure	1		
100188836	*CHDS8*	Coronary heart disease	1		
387584	*CHDS2*	Coronary heart disease	1		
5213	*PFKM*	Myocardial Ischemia (MI)	1		
325	*APCS*	Heart failure	1		
80350	*LPAL2*	Coronary heart disease	1		
4599	*MX1*	Myocardial Ischemia (MI)	1		
7941	*PLA2G7*	Coronary heart disease; Asthma	2		
301	*ANXA1*	Hypertensive disease	1		
26	*AOC1*	Hypertensive disease	1		

Entrez ID	Gene	CVDs (DisGeNET db v4.0)	CVD number	EV-miRNA targets	EV-miRNA number
100233226	*MCI2*	Myocardial Infarction	1		
475	*ATOX1*	Hypertensive disease	1		
345	*APOC3*	Coronary heart disease; Cardiovascular Diseases	2		
7349	*UCN*	Inflammation; Hypertensive disease	2		
101928940	*LOC101928940*	Asthma	1		
3939	*LDHA*	Myocardial Infarction	1		
117156	*SCGB3A2*	Asthma	1		
185	*AGTR1*	Heart failure; Hypertensive disease	2		
1375	*CPT1B*	Myocardial Ischemia (MI)	1		
101928947	*LOC101928947*	Asthma	1		
914	*CD2*	Thrombosis	1		
4843	*NOS2*	Heart failure; Hypertensive disease; Asthma; Inflammation; Myocardial Infarction	5		
3123	*HLA-DRB1*	Asthma	1		
2952	*GSTT1*	Hypertensive disease	1		
6037	*RNASE3*	Asthma	1		
2641	*GCG*	Heart failure; Hypertensive disease; Heart Diseases	3		
28984	*RGCC*	Myocardial Ischemia (MI)	1		
100048912	*CDKN2B-AS1*	Myocardial Infarction; Coronary heart disease	2		
3957	*LGALS2*	Myocardial Infarction	1		
387585	*CHDS4*	Coronary heart disease	1		
11141	*IL1RAPL1*	Cardiovascular Diseases	1		
2796	*GNRH1*	Hypertensive disease	1		

Entrez ID	Gene	CVDs (DisGeNET db v4.0)	CVD number	EV-miRNA targets	EV-miRNA number
3164	*NR4A1*	Myocardial Ischemia (MI)	1		
2161	*F12*	Hypertensive disease	1		
3163	*HMOX2*	Cerebral Hemorrhage	1		
64805	*P2RY12*	Myocardial Infarction; Thrombosis	2		
5693	*PSMB5*	Myocardial Ischemia (MI)	1		
7450	*VWF*	Cardiovascular Diseases; Heart failure; Atrial Fibrillation; Thrombosis; Hypertensive disease	5		
1440	*CSF3*	Heart Diseases; Heart failure	2		
6647	*SOD1*	Ischemia; Asthma; Inflammation; Myocardial Infarction; Heart failure; Hypertensive disease	6		
100130503	*LOC100130503*	Asthma	1		
7518	*XRCC4*	Hypertensive disease	1		
627	*BDNF*	Inflammation	1		
713	*C1QB*	Myocardial Ischemia (MI)	1		
2147	*F2*	Myocardial Infarction; Thrombosis	2		
3440	*IFNA2*	Myocardial Infarction	1		
8942	*KYNU*	Inflammation	1		
7148	*TNXB*	Hypertensive disease	1		
257160	*RNF214*	Cardiovascular Diseases	1		
2153	*F5*	Myocardial Infarction; Thrombosis	2		
348	*APOE*	Hypertensive disease; Coronary heart disease; Cardiovascular Diseases;	4		

Entrez ID	Gene	CVDs (DisGeNET db v4.0)	CVD number	EV-miRNA targets	EV-miRNA number
		Myocardial Infarction			
79001	*VKORC1*	Coronary heart disease	1		
1649	*DDIT3*	Myocardial Infarction	1		
476	*ATP1A1*	Myocardial Ischemia (MI); Hypertensive disease	2		
6139	*RPL17*	Myocardial Ischemia (MI)	1		
3665	*IRF7*	Inflammation	1		
387573	*CHDS3*	Coronary heart disease	1		
3934	*LCN2*	Inflammation	1		
9311	*ASIC3*	Inflammation	1		
5473	*PPBP*	Hypertensive disease	1		
51131	*PHF11*	Asthma	1		
7386	*UQCRFS1*	Myocardial Ischemia (MI)	1		
100188877	*CHDS9*	Coronary heart disease	1		
1329	*COX5B*	Myocardial Ischemia (MI)	1		
56955	*MEPE*	Cardiovascular Diseases	1		
7096	*TLR1*	Asthma	1		
9518	*GDF15*	Myocardial Ischemia (MI); Heart failure	2		
2833	*CXCR3*	Ischemia; Inflammation	2		
3565	*IL4*	Asthma	1		
5972	*REN*	Myocardial Infarction; Ischemia; Hypertensive disease; Heart failure	4		
7846	*TUBA1A*	Myocardial Ischemia (MI)	1		
440823	*MIAT*	Myocardial Infarction	1		
389036	*ACT*	Hypertensive disease	1		
4625	*MYH7*	Myocardial Infarction	1		

Entrez ID	Gene	CVDs (DisGeNET db v4.0)	CVD number	EV-miRNA targets	EV-miRNA number
117579	*RLN3*	Myocardial Ischemia (MI)	1		
3303	*HSPA1A*	Myocardial Ischemia (MI)	1		
5617	*PRL*	Heart failure	1		
9351	*SLC9A3R2*	Hypertensive disease	1		
4318	*MMP9*	Asthma; Inflammation; Myocardial Infarction; Cerebral Hemorrhage	4		
5443	*POMC*	Cerebral Hemorrhage; Heart failure; Hypertensive disease; Myocardial Ischemia (MI)	4		
885	*CCK*	Inflammation	1		
3240	*HP*	Hypertensive disease; Cardiovascular Diseases; Myocardial Infarction	3		
4282	*MIF*	Inflammation	1		
4846	*NOS3*	Heart failure; Atrial Fibrillation; Hypertensive disease; Ischemia; Myocardial Infarction; Myocardial Ischemia (MI); Cardiovascular Diseases	7		
9992	*KCNE2*	Atrial Fibrillation	1		
100528007	*C10orf32-ASMT*	Hypertensive disease	1		
4018	*LPA*	Cardiovascular Diseases; Coronary heart disease	2		
3630	*INS*	Heart failure; Hypertensive disease	2		
4852	*NPY*	Cardiovascular Diseases; Asthma	2		
1187	*CLCNKA*	Hypertensive disease	1		

**Entrez ID**	**Gene**	**CVDs (DisGeNET db v4.0)**	**CVD number**	**EV-miRNA targets**	**EV-miRNA number**
5160	*PDHA1*	Myocardial Ischemia (MI)	1		
2621	*GAS6*	Thrombosis	1		
6890	*TAP1*	Myocardial Ischemia (MI)	1		
59	*ACTA2*	Ischemia; Myocardial Infarction	2		
1557	*CYP2C19*	Thrombosis; Cardiovascular Diseases	2		
5244	*ABCB4*	Inflammation	1		
4792	*NFKBIA*	Myocardial Ischemia (MI)	1		
100507308	*TARID*	Coronary heart disease	1		
2992	*GYG1*	Myocardial Ischemia (MI); Cardiac Arrhythmia	2		
6336	*SCN10A*	Atrial Fibrillation	1		
8701	*DNAH11*	Coronary heart disease	1		
6351	*CCL4*	Myocardial Ischemia (MI); Inflammation	2		
63036	*CELA2A*	Hypertensive disease	1		
10911	*UTS2*	Hypertensive disease	1		
3315	*HSPB1*	Heart failure	1		
338334	*CHDS1*	Coronary heart disease	1		
335	*APOA1*	Hypertensive disease; Inflammation	2		
7200	*TRH*	Hypertensive disease; Cardiac Arrhythmia	2		
101929231	*LOC101929231*	Asthma	1		
796	*CALCA*	Inflammation; Hypertensive disease	2		
148738	*HFE2*	Coronary heart disease	1		
341	*APOC1*	Cardiovascular Diseases	1		
3816	*KLK1*	Myocardial Infarction;	2		

Entrez ID	Gene	CVDs (DisGeNET db v4.0)	CVD number	EV-miRNA targets	EV-miRNA number
		Hypertensive disease			
55759	*WDR12*	Coronary heart disease; Myocardial Infarction	2		
875	*CBS*	Cardiovascular Diseases; Hypertensive disease	2		
2811	*GP1BA*	Hypertensive disease; Thrombosis	2		
1471	*CST3*	Cardiovascular Diseases	1		
6490	*PMEL*	Asthma	1		
6347	*CCL2*	Hypertensive disease; Asthma; Inflammation; Cardiovascular Diseases; Myocardial Ischemia (MI)	5		
149773	*APCDD1L-AS1*	Cardiovascular Diseases	1		
282617	*IFNL3*	Asthma	1		
3290	*HSD11B1*	Hypertensive disease	1		
51738	*GHRL*	Heart failure	1		
414764	*HCG23*	Asthma	1		
183	*AGT*	Myocardial Ischemia (MI); Cardiovascular Diseases; Heart Diseases; Thrombosis; Hypertensive disease; Cardiac Arrhythmia; Myocardial Infarction; Inflammation; Heart failure	9		
6696	*SPP1*	Cerebral Hemorrhage; Heart Diseases	2		
3988	*LIPA*	Coronary heart disease	1		
1543	*CYP1A1*	Hypertensive disease	1		
5196	*PF4*	Thrombosis	1		

**Entrez ID**	**Gene**	**CVDs (DisGeNET db v4.0)**	**CVD number**	**EV-miRNA targets**	**EV-miRNA number**
11277	*TREX1*	Inflammation	1		
328	*APEX1*	Heart Diseases	1		
1215	*CMA1*	Hypertensive disease	1		
929	*CD14*	Asthma	1		
348932	*SLC6A18*	Hypertensive disease	1		
3557	*IL1RN*	Asthma; Inflammation; Myocardial Infarction	3		
1152	*CKB*	Myocardial Infarction	1		
7040	*TGFB1*	Asthma; Inflammation; Myocardial Infarction; Hypertensive disease	4		
1767	*DNAH5*	Asthma	1		
54106	*TLR9*	Inflammation	1		
2688	*GH1*	Cardiovascular Diseases	1		
